# CDK4/6 Inhibition Induces CD8^+^ T Cell Antitumor Immunity via MIF‐Induced Functional Orchestration of Tumor‐Associated Macrophages

**DOI:** 10.1002/advs.202511330

**Published:** 2025-10-13

**Authors:** Lin He, Yuzhong Peng, Lat‐lun Leong, Jingbo Zhou, Dongyang Tang, Weilu Wang, Xiaoran Wu, Josh haipeng Lei, Yongqin Ye, Yangyang Feng, Yunfeng Qiao, Xiangpeng Chu, Di Mu, Qi Zhao, Tzuming Liu, Yan Chen, Paul Kwonghang Tam, Chu‐Xia Deng

**Affiliations:** ^1^ Cancer Center Faculty of Health Sciences University of Macau Macau SAR 999078 China; ^2^ Center for Precision Medicine Research and Training Faculty of Health Sciences University of Macau Macau SAR 999078 China; ^3^ MOE Frontier Science Center for Precision Oncology University of Macau Macau SAR 999078 China; ^4^ Medical Sciences Division Macau University of Science and Technology Macau SAR 999078 China; ^5^ Precision Regenerative Medicine Research Centre Macau University of Science and Technology Macau SAR 999078 China; ^6^ University Hospital Macau University of Science and Technology Macau SAR 999078 China; ^7^ Guangdong Key Laboratory of New Technology in Rice Breeding Rice Research Institute Guangdong Academy of Agricultural Sciences Guangzhou 510640 China

**Keywords:** CDK4/6 inhibitor, CD8^+^ T cell, cell–cell communication, tumor immune microenvironment, tumor‐associated macrophage

## Abstract

Cyclin‐dependent kinases 4 and 6 (CDK4/6) regulate cell cycle progression from the G_1_ to S phase. Recently, CDK4/6 inhibition (CDK4/6i) is demonstrated to enhance antitumor immunity, as evidenced by increased tumor infiltration of CD8^+^ T cells; however, the mechanism underlying this phenomenon remains unclear. This study reveals that CDK4/6i enhances intratumoral CD8^+^ T cell infiltration in breast tumors through functional reprogramming of tumor‐associated macrophages (TAMs), facilitating indirect interactions between tumor and CD8^+^ T cells. Mechanistically, CDK4/6i enhances the accumulation and activation of M1 TAMs and promotes the M2 to M1 polarization via augmented interaction of the macrophage migration inhibitory factor (MIF)‐CD44/CD74 axis between tumor cells and macrophages. CDK4/6i drives tumor cells to secrete MIF by activating the HIF‐1α pathway. CDK4/6i‐trained M1 TAMs increase the population of CD8^+^ T cells and activate them through the MHC‐I antigen presentation machinery. Inhibition of MIF or loss of *Mif* in tumor cells reverses the immunostimulatory effects of CDK4/6i on macrophages and subsequent CD8^+^ T cell antitumor immunity. Therefore, CDK4/6i‐trained M1 TAM supernatant therapy surmounts the immunosuppressive tumor microenvironment and induces a tumor response to low‐dose PD‐1 immune checkpoint blockade therapy in breast cancers.

## Graphical abstract

CDK4/6 inhibition promotes CD8^+^ T cell expansion through tumor‐macrophage crosstalk by activating HIF‐1α and enhancing MIF‐CD44/CD74 signaling. This reprograms TAMs to boost MHC‐I antigen presentation, and CDK4/6 inhibitor‐trained M1 TAM supernatant therapy synergizes with low‐dose PD‐1 blockade to restore antitumor immunity.

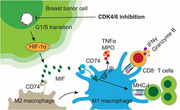

## 1 Introduction

Cell cycle dysregulation is a hallmark of carcinogenesis.^[^ ^1^, ^2^ ^]^ Cyclin‐dependent kinases 4 and 6 (CDK4/6) control cell cycle progression from the G_1_ phase to the S phase. The CDK4/6 activity is exemplified by hyperphosphorylation of retinoblastoma protein (Rb) and the resultant activation of the central E2F transcription factor, which drives cell cycle progression.^[^ ^3^ ^]^ CDK4/6 inhibition restrains this pathway and induces tumor cell cycle arrest at the G_1_ phase.^[^ ^4^ ^]^ Four CDK4/6 inhibitors (CDK4/6i)**—**palbociclib, ribociclib, abemaciclib, and trilaciclib**—**have been approved by the U.S. Food and Drug Administration (FDA) for cancer therapeutics. Palbociclib, ribociclib, and abemaciclib are administered for the treatment of advanced luminal human epidermal growth factor receptor 2 (HER2)‐negative breast cancers, whereas trilaciclib is specifically used to prevent chemotherapy‐induced damage to normal bone marrow cells in patients with small cell lung cancer.^[^ ^3^, ^5^ ^]^ Notably, CDK4/6i not only displays tumoricidal activity but also regulates immune cells within the tumor ecosystem.^[^ ^5^, ^6^, ^7^, ^8^, ^9^ ^]^

The tumor immune microenvironment (TIME) plays a crucial role in controlling cancer activity. Escape from immune surveillance is another hallmark of cancer.^[^ ^1^, ^2^ ^]^ Programmed cell death protein 1 (PD‐1) is a coinhibitory receptor on CD8^+^ T cells. PD‐1 immune checkpoint blockade (ICB) has emerged as a standard‐of‐care treatment for various advanced solid tumors; however it has an objective response rate of only 14.2%–33%.^[^ ^10^, ^11^, ^12^, ^13^ ^]^ Notably, the overwhelming majority of patients who benefit from this treatment experience lasting tumor regression and a significant prolongation of overall survival.^[^ ^14^, ^15^, ^16^ ^]^ Therefore, combining therapies that potentiate CD8^+^ T cell activation with PD‐1 ICB therapy may maximize the benefits for cancer patients. Alterations in the CDK4/6 pathway are related to acquired resistance to ICB.^[^ ^17^ ^]^ CDK4/6i enhances antitumor immunity via several mechanisms: 1) increased tumor infiltration of CD8^+^ T cells^[^ ^8^, ^18^, ^19^, ^20^ ^]^; 2) activation of CD8^+^ T cells^[^ ^3^, ^5^, ^8^, ^9^, ^18^, ^21^ ^]^; 3) enhanced proliferation kinetics of CD8^+^ T cells^[^ ^5^, ^22^ ^]^; and 4) remodeling of the TIME.^[^ ^19^, ^23^ ^]^ The synergistic effect of the CDK4/6i and PD‐1 ICB combination has been demonstrated in many preclinical cancer models, resulting in tumor shrinkage and significantly enhanced overall survival.^[^ ^9^, ^19^, ^20^, ^24^, ^25^, ^26^ ^]^ However, the precise mechanisms by which CDK4/6i invokes the proliferation and activation of CD8^+^ T cells remain unclear.

Macrophages exhibit significant plasticity, which is essential for responding to pathogenic cues and maintaining tissue homeostasis.^[^ ^27^ ^]^ Within the tumor microenvironment (TME), tumor‐associated macrophages (TAMs) interact with multiple cell types, including tumor cells, T cells, and fibroblasts, ultimately promoting tumor growth and immune escape. However, in the initial stages, TAMs play two crucial roles in dampening tumor development—eliminating malignant cells via phagocytosis and amplifying inflammatory signals to initiate systemic immune responses. Damage‐associated molecular patterns (DAMPs) produced by damaged cells are detected by pattern‐recognition receptors (PRRs) on macrophages.^[^ ^28^ ^]^ The binding of PRRs to DAMPs activates inflammatory responses and recruits adaptive immune cells. Conversely, the rapid replication and apoptosis of tumor cells continuously release DAMPs, triggering chronic inflammation and promoting tumor progression.^[^ ^29^ ^]^ Notably, tumor cells exploit the proinflammatory functions of macrophages by producing macrophage polarization‐related hematopoietic growth factors, such as granulocyte‐macrophage colony‐stimulating factors (GM‐CSFs/CSF‐2) and macrophage colony‐stimulating factors (M‐CSFs/CSF‐1).^[^ ^30^ ^]^ TAMs elicit CD8^+^ T cell antitumor immunity via two main functions—the release of neoantigens and cytokines and presentation of antigens on the macrophage surface for recognition by the T cell receptor on CD8^+^ T cells.

In this study, we utilized breast tumor mouse models, as well as murine and human breast tumor slices, to investigate the underlying mechanisms. We found that CDK4/6i enhanced the proliferation of intratumoral macrophages and CD8^+^ T cells through a tumor cell‐macrophage‐CD8^+^ T cell loop. CDK4/6i prompted tumor cells to release macrophage migration inhibitory factor (MIF), which induced the functional reprogramming of TAMs. The MIF‐induced M1 TAMs activated CD8^+^ T cell‐dependent antitumor immunity via the MHC‐I antigen presentation machinery. A similar enhancement of CD8^+^ T cell antitumor immunity was observed with CDK4/6i‐trained M1 TAM supernatant therapy. Our findings reveal that this supernatant pretreatment enhances a tumor response to low‐dose PD‐1 ICB therapy in breast tumors, presenting a novel therapeutic approach to boost cancer immunotherapy.

## 2 Results

### 2.1 CDK4/6i Increases Intratumoral Macrophage and CD8^+^ T Cell Populations and the M1/M2 Ratio

**Figure 1:**
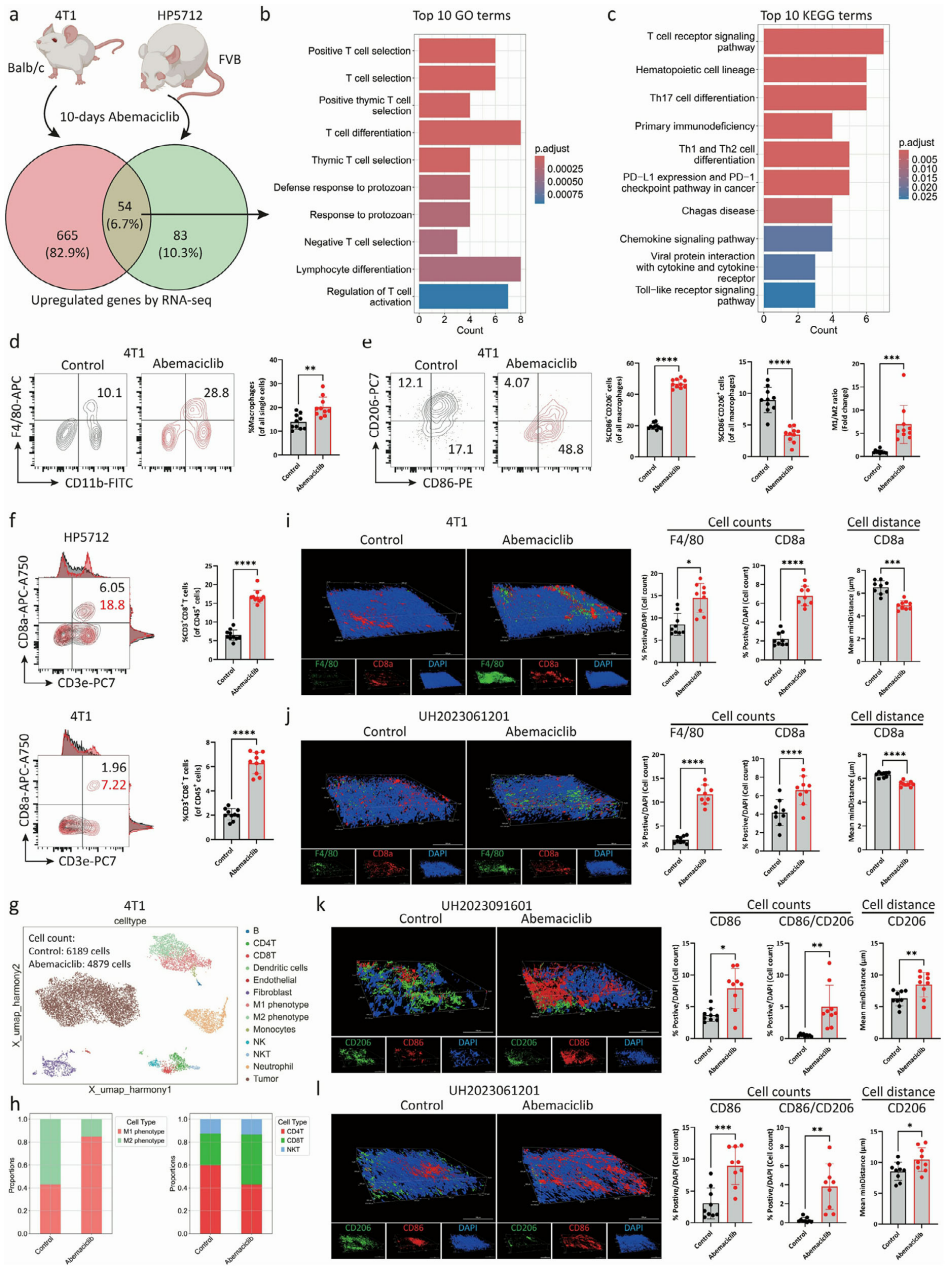
CDK4/6 inhibition increases the intratumoral macrophage and CD8^+^ T cell populations. a–c). Schematic for Venn diagram a), top 10 gene ontology (GO) terms b), and top 10 Kyoto encyclopedia of genes and genomes (KEGG) terms c) enriched for common abemaciclib‐upregulated genes in Balb/c mice orthotopically implanted with 4T1 cells and FVB mice orthotopically implanted with HP5712 cells (*n* = 3, for each). d,e) Intratumoral macrophages d), M1 and M2 phenotypes, and fold changes in the M1/M2 ratio e) in Balb/c mice orthotopically injected 4T1 cells after 10 days of control or abemaciclib treatment (*n* = 10, for each). f) Representative FACS plots and quantification of intratumoral CD8^+^ T cells in immunocompetent mice orthotopically injected HP5712 (upper) and 4T1 (lower) cells after 10 days of control or abemaciclib treatment (*n* = 10, for each). g) Uniform manifold approximation and projection of cell type annotation for Balb/c mice orthotopically injected 4T1 cells. h) Quantification of macrophage subtypes and T cell subtypes from Balb/c mice orthotopically injected 4T1 cells after control or abemaciclib treatment. i) Representative 3D‐immunofluorescence (3D‐IF) images, quantification of F4/80‐positive (green) and CD8a‐positive (red) cells, and analysis of the mean minimal distance of CD8a‐positive cells in 4T1 tumors after control or abemaciclib in vivo treatment (3 areas × 3 samples, for each); scale bar = 100 µm. j) Representative 3D‐IF images, quantification of F4/80‐positive (green) and CD8a‐positive (red) cells, and analysis of the mean minimal distance of CD8a‐positive cells in human TNBC tumor slices after 5 days of control or abemaciclib *ex vivo* treatment (3 areas × 3 samples, for each); scale bar = 100 µm. k,l) Representative 3D‐IF images of CD86 (red) and CD206 (green) expression, quantification of CD86‐positive cells and CD86/CD206 ratio, and analysis of the mean minimal distance of CD206‐positive cells in human luminal breast cancers k) and TNBC l) after 5 days of control or abemaciclib *ex vivo* treatment (3 areas × 3 samples, for each); scale bar = 100 µm. *p*‐values are calculated using unpaired two‐tailed *t*‐test (d–f, i–l). Data presented as mean ± SD. ^*^ *p* < 0.05; ^**^ *p* < 0.01; ^****^ *p* < 0.0001.

Two murine breast cancer cell lines, HP5712 and 4T1, were used to study the responses to the CDK4/6 inhibitors abemaciclib and palbociclib (Figure S1A, Supporting Information). Both inhibitors induced dose‐dependent cytotoxicity and tumor cell arrest at the G_1_ phase (Figure S1A–C, Supporting Information). We then investigated the effect of abemaciclib on orthotopically implanted tumors by injecting HP5712 cells into the mammary gland of FVB mice, which resulted in a significantly reduced tumor volume (Figure S1D–E, Supporting Information). Notably, RNA‐seq analysis revealed 54 upregulated genes that served as common regulators in immunocompetent mice implanted with 4T1 and HP5712 tumors (**Figure** **1**a). The GO analysis of these upregulated genes showed that the top 10 candidate pathways were involved in T cell selection, T cell differentiation, T cell activation, and lymphocyte differentiation (Figure 1b). Consistently, the Kyoto encyclopedia of genes and genomes (KEGG) analysis indicated that these genes were primarily enriched in T cell activation and macrophage reprogramming pathways (Figure 1c). Flow cytometric analysis showed that abemaciclib significantly increased the population of intratumoral lymphocytes, macrophages, M1 macrophages (i.e., CD86^+^CD206^−^), and CD8^+^ T cells, while decreasing the population of M2 macrophages (i.e., CD206^+^CD86^−^), thereby increasing the M1/M2 ratio in HP5712 and 4T1 tumors (Figure 1d–f; Figure S1F–G, Supporting Information). However, in spleens from Balb/c mice with 4T1 tumors, the GSEA and GO terms of abemaciclib‐upregulated genes were only enriched for canonical pathways, such as G_1_/S cell cycle regulation and mitotic G_1_/S transition (Figure S1H,I, Supporting Information). Flow cytometry analysis indicated that abemaciclib markedly decreased the population of both M1 and M2 macrophages and did not alter the total populations of macrophages and CD8^+^ T cells, or the M1/M2 ratio (Figure S1J–K, Supporting Information). These findings indicated that CDK4/6i increases the accumulation of intratumoral macrophages and CD8^+^ T cells and converts TAMs into an immunostimulatory state.

To further support our findings, single‐cell RNA sequencing (scRNA‐seq) was performed on Balb/c mice implanted with 4T1 tumors with or without abemaciclib treatment. The scRNA‐seq GO analyses of abemaciclib‐upregulated genes in tumor cells, M1 macrophages, and CD8^+^ T cells revealed involvement in inflammatory response and cell–cell adhesion (Figure S2A–E, Supporting Information). Notably, the scRNA‐seq data revealed an increased M1/M2 ratio, a higher proportion of CD8^+^ T cells, and a lower proportion of CD4^+^ T cells following abemaciclib treatment (Figure 1g–h).

Next, we utilized a 3D tumor slice culture (3D‐TSC) platform^[^ ^31^, ^32^ ^]^ to determine whether CDK4/6i could increase the number of intratumoral macrophages and CD8^+^ T cells *ex vivo* (Figure S3A, Supporting Information). 3D immunofluorescence (3D‐IF) microscopy revealed a significant increase in macrophage and CD8^+^ T cell accumulation, accompanied by a significant decrease in the neighborhood distance between CD8^+^ T cells in 4T1 tumor slices following in vivo treatment with abemaciclib (Figure 1i; Figure S3B, Supporting Information). *Ex vivo* treatment with abemaciclib also increased the number of total lymphocytes, CD3^+^ T cells, and CD8^+^ T cells, but did not change the CD4^+^ T cell population (Figure S3C,D, Supporting Information). Previous evidence suggests that CD28, in conjunction with the T cell antigen receptor, can initiate the proliferation and activation of T cells.^[^ ^33^ ^]^ *Ex vivo* treatment with abemaciclib significantly increased CD28 expression on the surface of CD8^+^ T cells in tumor slices, supporting the expansion of CD8^+^ T cells by CDK4/6i (Figure S3E, Supporting Information). Notably, *ex vivo* treatment with abemaciclib in human luminal HER2‐negative and triple‐negative breast cancer (TNBC) tumor slices resulted in increased accumulation of macrophages, CD8^+^ T cells, and M1 macrophages; an increased CD86/CD206 ratio; a decreased neighborhood distance among CD8^+^ T cells; and an increased neighborhood distance of M2 macrophages (Figure 1j–l; Figure S3F–G, Supporting Information).

To determine the dynamic changes in macrophages following CDK4/6i treatment, we used albino B6 (C2J)/LysM‐GFP mice^[^ ^34^ ^]^ orthotopically implanted with the mesenchymal‐like murine breast adenocarcinoma Py8119 tumors. The Py8119 tumor slices were harvested for *ex vivo* treatment with abemaciclib (Figure S4A, Supporting Information). The GFP intensity, representing macrophages, became more pronounced after 3 days of treatment and continued to increase until day 5, even after drug removal, indicating an immune‐like macrophage memory. On day 8, the GFP intensity remained significantly higher in the abemaciclib treatment group compared with that in the control drug‐free group (Figure S4A,B, Supporting Information). Next, we investigated whether the increase in macrophages induced by CDK4/6i was tumor‐dependent. RAW264.7 cells and polarized RAW264.7 M1 macrophages were viably labeled with CFSE and treated with abemaciclib for 2, 5, and 7 days. The CFSE fluorescence in the abemaciclib group was significantly higher than that in the control group, indicating reduced proliferation of macrophages and M1 phenotypes following CDK4/6i treatment (Figure S4C,D, Supporting Information). Using tumor‐free albino B6 (C2J)/LysM‐GFP mice‐derived kidney and liver slices, the abemaciclib group was not found to exhibit any increase in the GFP intensity compared with that in the control group, even after 5 days of treatment (Figure S4E,F, Supporting Information). Therefore, CDK4/6i induced an immune‐like macrophage memory specific to the TME.

### 2.2 CDK4/6i Enhances Intratumoral Macrophage and CD8^+^ T Cell Accumulation Through the Tumor Cell–Macrophage–CD8^+^ T Cell Loop

**Figure 2:**
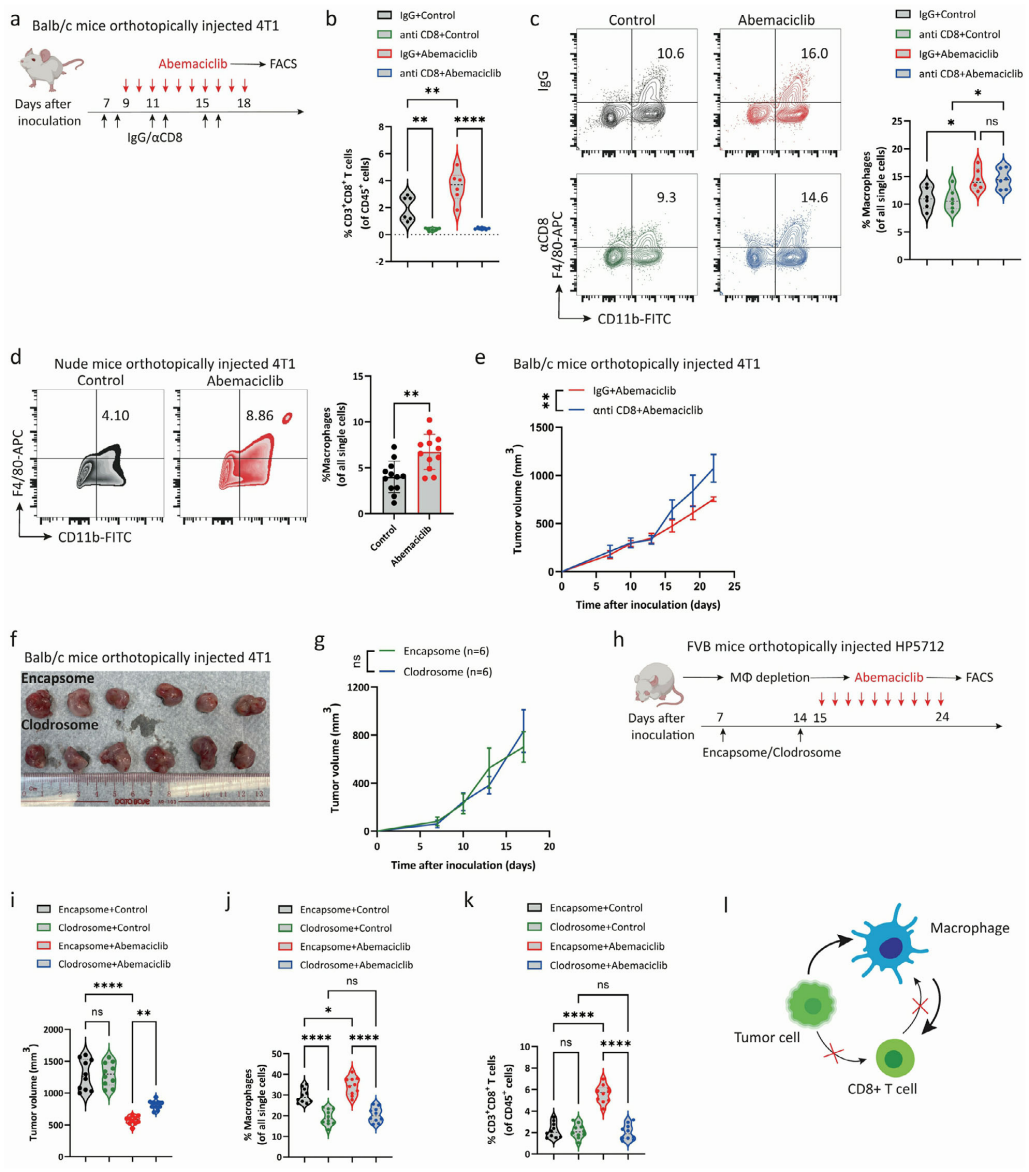
CDK4/6 inhibition expands the intratumoral CD8^+^ T‐cell population via the tumor cell–macrophage–CD8^+^ T cell loop. a–c) Workflow a), and intratumoral CD8^+^ T cell b) and macrophage c) populations from Balb/c mice orthotopically injected 4T1 cells after IgG or anti‐CD8 depletion plus control or abemaciclib treatment (*n* = 6, for each). d) Intratumoral macrophage population from nude mice orthotopically injected 4T1 cells after 10 days of control or abemaciclib treatment (*n* = 12, for each). e) Changes in tumor volume in Balb/c mice orthotopically injected 4T1 cells after IgG plus abemaciclib or anti‐CD8 plus abemaciclib in vivo treatment (*n* = 6, for each). f,g) Representative images of tumors f) and changes in tumor volume g) in FVB mice orthotopically injected HP5712 cells after encapsome or clodrosome treatment (*n* = 6, for each). h–k) Workflow h), comparison of tumor volume i), and intratumoral macrophage j) and CD8^+^ T cell k) populations in FVB mice orthotopically injected HP5712 cells after encapsome or clodrosome pretreatment plus control or abemaciclib treatment (*n* = 10, for each). l) Schematic of the tumor cell, macrophage, and CD8^+^ T cell interaction loop after abemaciclib treatment. *P*‐values are calculated using one‐way ANOVA corrected for multiple comparisons b,c, i–k), or unpaired two‐tailed *t*‐test d,e,g). Data presented as mean ± SD. ns, *p* > 0.05; ^*^ *p* < 0.05; ^**^ *p* < 0.01; ^****^ *p* < 0.0001.

We investigated whether CDK4/6i increased the population of intratumoral macrophages and CD8^+^ T cells individually or through their immunoregulatory interactions. After CD8^+^ T cell depletion in Balb/c mice orthotopically injected 4T1 cells, macrophage populations were still expanded by abemaciclib treatment in tumors but not in the spleen (**Figure** **2**a–c; Figure S5A,B, Supporting Information). Similarly, abemaciclib treatment remarkably increased the macrophage population in immunodeficient mice implanted with 4T1 tumors (Figure 2d). In a previous study, CD8^+^ T cells were reported to be important for the in vivo tumor‐killing effect of CDK4/6i^6^. Indeed, we observed a significant reduction in tumor volume control with abemaciclib treatment following CD8^+^ T cell depletion in immunocompetent mice implanted with 4T1 tumors (Figure 2e). Next, we treated spleen‐isolated naïve CD8^+^ T cells from FVB mice with an anti‐CD8 antibody (10 µg mL^−1^) and found that CD8a expression was completely lost in these cells (Figure S5C, Supporting Information). The CD8^+^ T cell populations were also markedly depleted by 10 µg mL^−1^ of anti‐CD8 antibody after 2 days of treatment of the 4T1 tumor slices (Figure S5D, Supporting Information). The antitumor activity of abemaciclib in immunocompetent mice implanted with HP5712 and 4T1 tumor slices was significantly reduced after the removal of CD8^+^ T cells (Figure S5E, Supporting Information). Additionally, macrophages were expanded by abemaciclib in both types of tumor slices following CD8^+^ T cell depletion (Figure S5F, Supporting Information). These data indicated that CD8^+^ T cells are key components for the tumor‐killing effects but not for the increased intratumoral accumulation of macrophages induced by CDK4/6i.

Next, we investigated the variation in the number of intratumoral CD8^+^ T cells following CDK4/6i treatment after macrophage removal by clodrosome, which has been demonstrated to effectively deplete macrophages in mouse models in vivo;^[^ ^35^ ^]^ the treatment did not reduce the tumor volume of 4T1 tumors (Figure 2f,g). Of note, the reduction in volume of HP5712 tumors by abemaciclib was significantly reversed by macrophage removal (Figure 2h,i), highlighting the key role played by macrophages in breast cancers subjected to CDK4/6i treatment. After macrophage depletion, abemaciclib failed to increase the intratumoral macrophage and CD8^+^ T cell populations (Figure 2j,k). Furthermore, we found that clodrosome (0.25 mg/mL) effectively killed macrophages with low cytotoxicity to HP5712 and 4T1 cells (Figure S5G, Supporting Information). Consistent with in vivo findings, abemaciclib treatment did not expand CD8^+^ T cells in tumor slices after macrophage depletion (Figure S5H, Supporting Information). Collectively, these findings indicated that CDK4/6i treatment increases the population of intratumoral CD8^+^ T cells via pre‐stimulation of TAMs (Figure 2l).

### 2.3 CDK4/6i Promotes the Proliferation and Activation of TAMs, and the M2 to M1 Polarization

As CDK4/6i induces cell cycle arrest at the G_1_/S transition, treating macrophages with abemaciclib alone should decrease their proliferation potential. We treated FVB mice bone marrow‐derived M1 (BM‐M1) and M2 (BM‐M2) macrophages with abemaciclib and observed a significant reduction in macrophage expansion (Figure S6A–C, Supporting Information). We harvested supernatants from abemaciclib‐treated tumor cells to stimulate FVB mouse BM‐M1 and BM‐M2 macrophages and could still find attenuated differentiation of macrophages (Figure 6D–E; Figure S6A, Supporting Information). These data indicated that the promotion of macrophage expansion by CDK4/6i treatment may be dependent on the TME.

**Figure 3:**
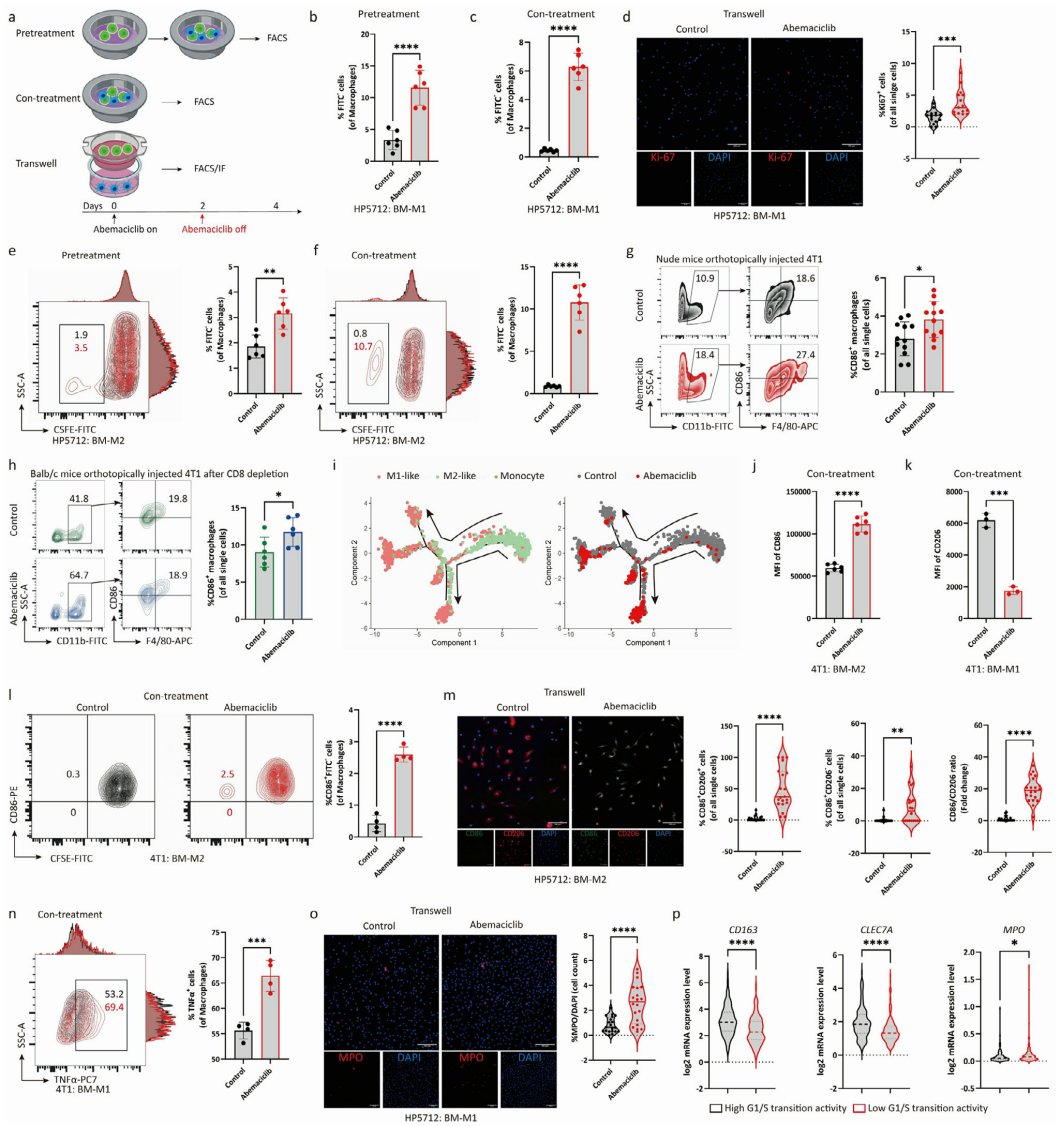
CDK4/6 inhibition enhances the immunostimulatory functionality of TAMs. a) Three different coculture models for detecting macrophage proliferation and activation potential. b,c) Quantification of the proliferation potential of FVB mouse bone marrow‐derived M1 (BM‐M1) macrophages cocultured with HP5712 after control or abemaciclib treatment in the pretreatment (b; *n* = 6, for each) or con‐treatment (c; *n* = 6, for each) model. d) Representative images and quantification of Ki‐67 expression (red) in FVB mouse BM‐M1 macrophages cocultured with HP5712 after control or abemaciclib treatment in the Transwell model (5 areas × 3 samples, for each); scale bar = 100 µm. e,f) Representative FACS plots and quantification of the proliferation potential of FVB mouse BM‐M2 macrophages cocultured with HP5712 after control or abemaciclib treatment in the pretreatment (e; *n* = 6, for each) or con‐treatment (f; *n* = 6, for each) model. g) Intratumoral CD86^+^ macrophage populations from nude mice orthotopically injected 4T1 cells after 10 days of control or abemaciclib treatment (*n* = 12, for each). h) Intratumoral CD86^+^ macrophages from Balb/c mice orthotopically injected 4T1 cells after anti‐CD8 depletion plus control or anti‐CD8 depletion plus abemaciclib treatment (*n* = 6, for each). i) Pseudotime analysis of intratumoral M1 macrophages, M2 macrophages, and monocytes from Balb/c mice orthotopically injected 4T1 cells after 10 days of control or abemaciclib treatment. j) Quantification of CD86 expression on Balb/c mouse BM‐M2 macrophage cocultured with 4T1 after control or Abemaciclib treatment in the con‐treatment model (*n* = 6, for each). k) Quantification of CD206 expression on Balb/c mouse BM‐M1 macrophage cocultured with 4T1 after control or abemaciclib treatment in the con‐treatment model (*n* = 3, for each). l) Representative FACS plots and quantification of proliferated M1 macrophages polarized from Balb/c mouse BM‐M2 macrophages cocultured with 4T1 after control or abemaciclib treatment in the con‐treatment model (*n* = 4, for each). m) Representative images for CD86 (green) and CD206 (red) expression and quantification of CD86^+^CD206^+^ and CD86^+^CD206^−^ cells, and CD86/CD206 ratio in FVB mouse BM‐M2 macrophage cocultured with HP5712 after control or abemaciclib treatment in the Transwell model (5 areas × 4 samples, for each); scale bar = 100 µm. n) Representative FACS plots and quantification of TNF‐α expression on Balb/c mouse BM‐M1 macrophage cocultured with 4T1 in the con‐treatment model (*n* = 4, for each). o) Representative images and quantification of MPO expression (red) on FVB mouse BM‐M1 macrophage cocultured with HP5712 after control or abemaciclib treatment in the Transwell model (5 areas × 4 samples, for each); scale bar = 100 µm. p) The Cancer Genome Atlas analysis of biomarkers for M2 TAMs (*CD163* and *CLEC7A*) and M1 TAM activation (*MPO*) between high G1/S transition activity and low G1/S transition activity cohorts. *P*‐values are calculated using unpaired two‐tailed *t*‐test b–h, j–p). Data presented as mean ± SD. ^*^ *p* < 0.05; ^**^ *p* < 0.01; ^****^ *p* < 0.0001.

To determine whether CDK4/6i functionally reprograms TAMs through direct contact or by soluble factors between tumor cells and macrophages, we designed three different types of in vitro coculture models: pretreatment, con‐treatment, and Transwell models (**Figure** **3**a). Abemaciclib significantly enhanced the proliferation of both FVB mouse BM‐M1 and BM‐M2 macrophages in all the coculture models (Figure 3b–f). To determine whether this was an “on‐target” effect, we used another CDK4/6i, palbociclib, to treat Balb/c mouse BM‐M1 macrophages and FVB mouse BM‐M2 macrophages in the con‐treatment model. Palbociclib also increased the proliferation of both BM‐M1 and BM‐M2 macrophages, corroborating our findings (Figure S6F–G, Supporting Information).

It was, however, intriguing that CDK4/6i treatment also increased the proliferation of M2 macrophages in these coculture models. We hypothesized that CDK4/6i might first polarize M2 to M1 macrophages prior to expansion. Abemaciclib significantly enhanced intratumoral CD86^+^ macrophages in both nude mice orthotopically implanted with 4T1 tumors and Balb/c mice orthotopically implanted with 4T1 tumors followed by CD8^+^ T cell depletion (Figure 3g,h). The scRNA‐seq of 4T1 tumors indicated that abemaciclib treatment triggered the polarization of M2 to M1 macrophages (Figure 3i). Additionally, abemaciclib treatment significantly increased CD86 expression on Balb/c mouse BM‐M2 macrophages but decreased CD206 expression on Balb/c mouse BM‐M1 macrophages in the con‐treatment model (Figure 3j,k). Notably, the enhanced CD86 expression on FVB mouse BM‐M2 macrophages treated with abemaciclib was gradually reduced with the increased ratio of M2 macrophages in the con‐treatment model (Figure S6H, Supporting Information), indicating polarization in a ratio‐dependent manner. Abemaciclib‐proliferated Balb/c mouse BM‐M2 macrophages in the con‐treatment model belonged to the CD86^+^CFSE^−^ quadrant, that is, they were expanded M1 macrophages (Figure 3l), implying that CDK4/6i‐induced expansion of M2 macrophages required polarization to M1 macrophages. To corroborate our findings, we used HP5712 and FVB mouse BM‐M2 macrophages in the Transwell model and found that abemaciclib dramatically enhanced the percentage of CD86^+^CD206^+^ macrophages, CD86^+^CD206^−^ macrophages, and the CD86/CD206 ratio (Figure 3m). These data indicated that CDK4/6i does not directly induce M2 macrophage proliferation but rather polarized M2 to M1 macrophages toward further expansion.

Next, we investigated the activation status of M1 TAMs following CDK4/6i treatment. Consistently, abemaciclib significantly increased the TNFα levels in both FVB mice and Balb/c mouse BM‐M1 macrophages in the con‐treatment model (Figure 3n; Figure S6I, Supporting Information). The expression of MPO, a marker of macrophage activation,^[^ ^36^ ^]^ was also elevated in FVB mouse BM‐M1 macrophages treated with abemaciclib in the Transwell model (Figure 3o). TCGA analysis of breast cancers revealed significantly lower expression of biomarkers for M2 TAMs (*CD163* and *CLEC7A*) but higher *MPO* expression in patients with low G_1_/S transition activity (i.e., inhibited CDK4/6 activity) compared with that in patients with high G_1_/S transition activity (i.e., enhanced CDK4/6 activity) (Figure 3p). Collectively, these findings indicated that CDK4/6i‐treated tumor cells trigger an immunostimulatory functional reprogramming of TAMs via both cell–cell interactions and tumor‐derived soluble proteins.

### 2.4 CDK4/6i‐Trained M1 TAMs Trigger CD8^+^ T Cell Antitumor Immunity

**Figure 4:**
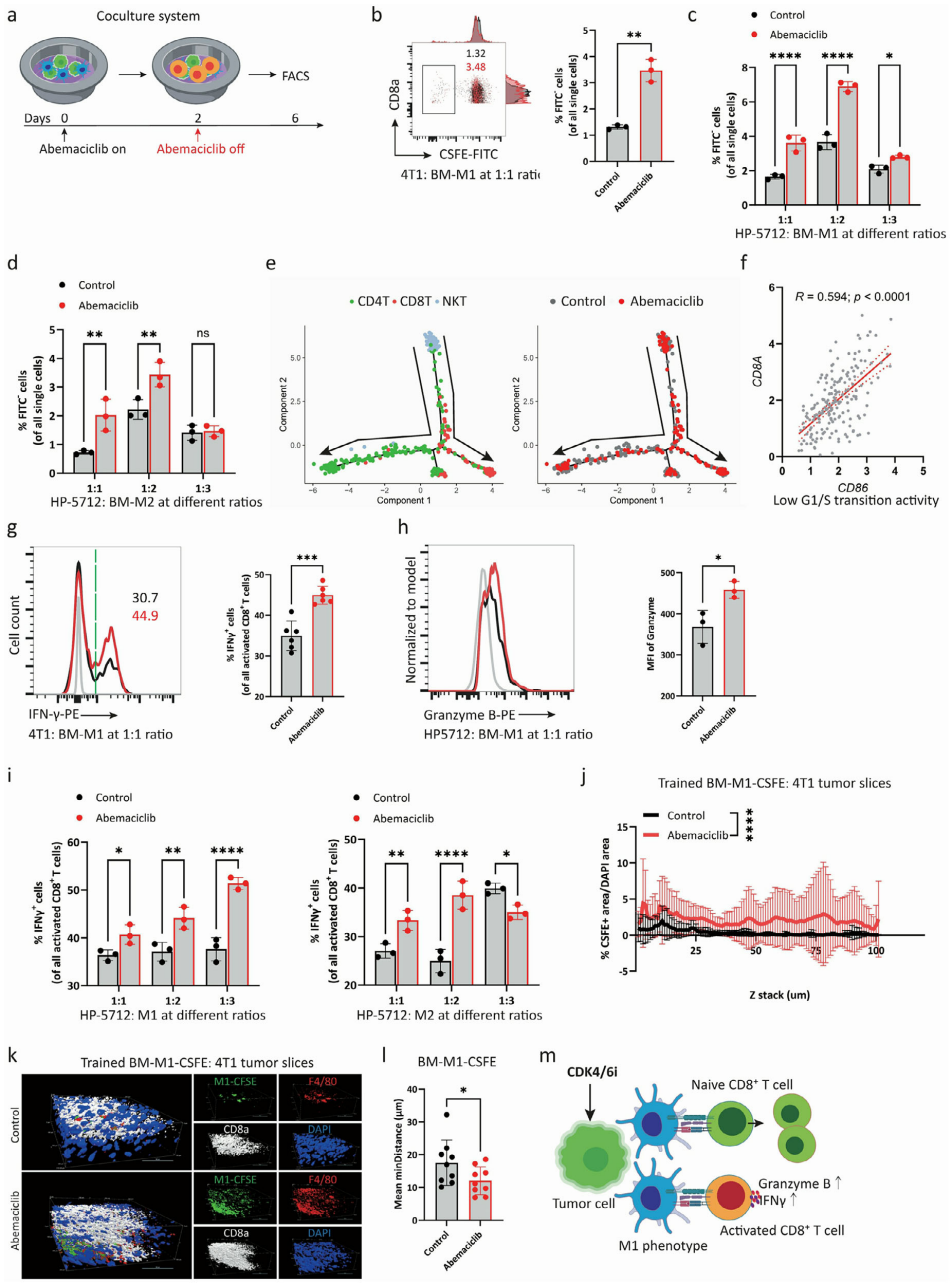
CDK4/6 inhibition‐trained M1 TAMs trigger CD8^+^ T cell antitumor immunity. a) Coculture system to detect the proliferation and activation of CD8^+^ T cells. b) Representative FACS plots and quantification of the proliferation potential of Balb/c mouse‐derived naïve CD8^+^ T cells in the coculture system of 4T1:Balb/c mouse BM‐M1 macrophages at a 1:1 ratio after control or abemaciclib treatment (*n* = 3, for each). c,d) Quantification of the proliferation potential of FVB mouse‐derived naïve CD8^+^ T cells in the coculture system of HP‐5712:FVB mouse BM‐M1 macrophages (c; *n* = 3, for each) or BM‐M2 macrophage (d; *n* = 3, for each) at 1:1 to 1:3 ratios after control or abemaciclib treatment. e) Pseudotime analysis of CD4^+^ T, CD8^+^ T, and NKT cells from Balb/c mice orthotopically injected 4T1 cells after 10 days of control or abemaciclib treatment. f) The Cancer Genome Atlas analysis of correlation between *CD8A* and *CD86* in the low G1/S transition activity cohort. g) Representative FACS histograms and quantification of IFNγ expression on the surface of Balb/c mice‐derived activated CD8^+^ T cells in the coculture system of 4T1:Balb/c mouse BM‐M1 macrophage at a 1:1 ratio after control or abemaciclib treatment (*n* = 6, for each). h) Representative FACS histograms and quantification of Granzyme B expression on FVB mouse‐derived activated CD8^+^ T cells in the coculture system of HP‐5712:FVB mouse BM‐M1 macrophage at a 1:1 ratio after control or abemaciclib treatment (*n* = 3, for each). i) Quantification of IFNγ expression on the surface of FVB mouse‐derived activated CD8^+^ T cells in the coculture system of HP‐5712:FVB mouse BM‐M1 macrophage (left; *n* = 3, for each) or HP‐5712:FVB mouse BM‐M2 macrophage (right; *n* = 3, for each) at 1:1 to 1:3 ratios after control or abemaciclib treatment. j) Penetration activity of control‐ or abemaciclib‐trained Balb/c mouse BM‐M1 macrophages stained with CFSE (BM‐M1‐CFSE) in Balb/c mice orthotopically injected 4T1 tumor slices after 8 days of coculture (3 areas × 3 samples, for each). k) Representative 3D‐immunofluorescence (3D‐IF) images of the interaction between control‐ or abemaciclib‐trained Balb/c mouse BM‐M1‐CFSE (green) and CD8^+^ T cells (white) in Balb/c mice orthotopically injected 4T1 tumor slices; scale bar = 100 µm. l) Quantification of the mean minimal distance between control‐ or abemaciclib‐trained Balb/c mouse BM‐M1‐CFSE cells in Balb/c mice orthotopically injected with 4T1 tumor slices. m. Schematic of tumor cells cocultured with M1 macrophages to enhance naïve CD8^+^ T‐cell proliferation and activated‐CD8^+^ T cell status. *P*‐values are calculated using unpaired two‐tailed *t*‐test b,g,h,j,l), or two‐way ANOVA corrected for multiple comparisons c,d,i). Data presented as mean ± SD. ^*^ *p* < 0.05; ^**^ *p* < 0.01; ^***^ *p* < 0.001; ^****^ *p* < 0.0001.

As the terminal fate of CDK4/6i‐treated M2 TAMs was polarization into M1 phenotypes, we hypothesized that M1 macrophages play a key role in mediating the tumor cell–macrophage–CD8^+^ T cell loop. We sought to determine whether tumor cells cocultured with M1 macrophages treated with CDK4/6i could increase the proliferation and activation of CD8^+^ T cells. We designed an in vitro coculture system wherein tumor cells and macrophages were cocultured together for 2 days with CDK4/6i treatment. Given the demonstrated immune‐like macrophage memory induced by CDK4/6i, we removed CDK4/6i on day 2 and directly added CD8^+^ T cells to the plate for an additional 4 days of coculture (**Figure** **4**a). Flow cytometry analysis indicated that abemaciclib significantly increased the proliferation of naïve CD8^+^ T cells derived from Balb/c mice (Figure 4b). In particular, we found that the proliferation potential of FVB mice‐derived naïve CD8^+^ T cells was enhanced by CDK4/6i in a coculture system with HP5712 cells and BM‐M1 macrophages at a 1:1 to 1:2 ratio; the potential was, however, attenuated at a 1:3 ratio but still exhibited significant changes (Figure 4c). Notably, abemaciclib treatment also enhanced the proliferation of naïve CD8^+^ T cells derived from FVB mice in a coculture system with HP5712 cells and BM‐M2 macrophages at 1:1 and 1:2 ratios, but not at a 1:3 ratio (Figure 4d). In vivo, scRNA‐seq data of 4T1 tumors revealed that T cells in the abemaciclib cohort were more likely to develop into CD8^+^ T cells, whereas those in the control group developed into CD4^+^ T cells (Figure 4e). TCGA analysis indicated a positive correlation between *CD8A* and *CD86* (*R* = 0.594) in patients with breast cancer exhibiting a low G_1_/S transition activity (Figure 4f). These data indicated that CDK4/6i‐induced CD8^+^ T cell proliferation requires the participation of M1 TAMs.

Next, we investigated the activation status of naïve and activated CD8^+^ T cells in the coculture system following CDK4/6i treatment. Abemaciclib‐enhanced IFNγ and granzyme B levels in FVB‐ and Balb/c mice‐derived activated CD8^+^ T cells were gradually amplified in the coculture system with tumor cells and BM‐M1 macrophages at ratios ranging from 1:1 to 1:3 (Figure 4g–i). In comparison, the IFNγ level in FVB mice‐derived activated CD8^+^ T cells was significantly increased by abemaciclib in the coculture system with tumor cells and BM‐M2 macrophages at 1:1 and 1:2 ratios, but significantly decreased at a 1:3 ratio (Figure 4i). Collectively, these findings indicated that M1 TAMs, but not M2 TAMs, play a major role in mediating the proliferation and activation of intratumoral CD8^+^ T cells following CDK4/6i treatment.

To determine whether CDK4/6i‐trained M1 TAM‐amplified CD8^+^ T cell antitumor immunity requires cell–cell contact or soluble factors, we designed a supernatant system and a Transwell‐1 model (Figure S7A,I, Supporting Information). In the supernatant system, abemaciclib treatment significantly attenuated the proliferation of FVB mice‐derived naïve CD8^+^ T cells (Figure S7B, Supporting Information). Moreover, no difference in IFNγ levels was observed in FVB and Balb/c mice‐derived naïve CD8^+^ T cells between the abemaciclib and control groups, regardless of the ratio of tumor cells to BM‐M1 macrophages (Figure S7C,D, Supporting Information). Additionally, abemaciclib treatment did not alter the levels of IFNγ and granzyme B in FVB mice‐derived activated CD8^+^ T cells, regardless of the phenotype or proportion of macrophages (Figure S7E–H, Supporting Information). Similarly, abemaciclib failed to significantly increase the proliferation of Balb/c mice‐derived naïve CD8^+^ T cells and IFNγ levels in Balb/c mice‐derived activated CD8^+^ T cells in the Transwell‐1 model (Figure S7J,K, Supporting Information). These findings indicated that CDK4/6i‐trained M1 TAM‐triggered CD8^+^ T cell antitumor immunity requires cell–cell interactions.

We investigated the effect of CDK4/6i‐trained M1 TAMs on the tumor immune ecosystem. For this, we designed an *ex vivo* coculture model using macrophages and tumor slices, wherein 5 × 10^4^ macrophages were evenly mixed in the super gel (Figure S8A, Supporting Information). The immune cell repertoire on the surface of 4T1 tumor slices was altered by RAW264.7 coculture, as indicated by higher F4/80 and CD8a expression in the coculture group (Figure S8B, Supporting Information). To further determine whether macrophages could penetrate the tumor slices and affect the interior immune cells, we cocultured RAW264.7‐GFP cells with the tumor slices. Indeed, flow cytometry analysis revealed the penetration of macrophages into HP5712 tumors (Figure S8C, Supporting Information). Notably, RAW264.7‐GFP M1 macrophages exhibited significantly higher penetration than did M2 macrophages (Figure S8D, Supporting Information). The enhanced penetration of RAW264.7‐GFP M1 macrophages into HP5712 and 4T1 tumors was confirmed via 3D‐IF analysis. Notably, the penetrating RAW264.7‐GFP M1 macrophages resulted in higher CD8a levels in both the tumor types than the levels observed for the M2 phenotype (Figure S8E,F, Supporting Information).

We trained Balb/c mouse BM‐M1 macrophages with or without 2 days of abemaciclib treatment in the Transwell model and harvested the M1 macrophages for CFSE labeling to coculture with 4T1 tumor slices for 8 days (Figure S8G, Supporting Information). Abemaciclib‐trained M1 macrophages displayed a significantly higher penetration capacity in tumors than did control‐trained M1 macrophages (Figure 4j; Figure S8H, Supporting Information). The abemaciclib‐trained M1 macrophages also resulted in markedly increased number of CD8^+^ T cells and granzyme B expression in tumors (Figure S8H, Supporting Information). Notably, a robust cell–cell interaction between the penetrated M1 macrophages and CD8^+^ T cells, along with a dramatic decrease in the neighborhood distance among the penetrated M1 macrophages, was observed in the abemaciclib treatment group (Figure 4k). These data indicated that CDK4/6i‐trained M1 macrophages in the TME provoked CD8^+^ T cell antitumor immunity, plausibly requiring the antigen presentation machinery (Figure 4l).

We further investigated whether CDK4/6i‐triggered TAM reprogramming and CD8^+^ T cell antitumor immunity were associated with the G_1_/S transition activity. We used a CDK4/6i‐resistant murine cell line, HP5712‐A009, derived from HP5712 cells. The upregulated genes in the HP5712‐A009 cell line compared with the HP5712 cell line were enriched in pathways related to the “cell cycle,” “apoptosis,” “positive regulation of cell cycle phase transition,” and particularly the “G_1_/S transition of the mitotic cell cycle” (Figure S9A,B, Supporting Information). This cell line exhibited higher pRb‐E2F axis activity and thereby demonstrated a faster G_1_/S transition (i.e., a lower proportion of G_1_ phase cells and a higher proportion of S phase cells) than did HP5712 during the log phase (Figure S9C,D, Supporting Information). The HP5712‐A009 cell line also showed higher cell viability than HP5712 after treatment with abemaciclib gradient concentrations (Figure S9E, Supporting Information). Abemaciclib still enhanced the proliferation potential of FVB mouse BM‐M1 macrophages but did not increase TNFα levels in BM‐M1 macrophages and decreased CD86 expression in BM‐M2 macrophages when cocultured with HP5712‐A009 cells (Figure S9F–H, Supporting Information). Notably, the proliferation of FVB mice‐derived naïve CD8^+^ T cells was decreased, and IFNγ expression in activated CD8^+^ T cells was not increased in the coculture system of HP5712‐A009 cells and BM‐M1 macrophages at a 1:1 ratio after abemaciclib treatment (Figure S9I,J, Supporting Information). These data indicate that the functional programming of TAMs and CD8^+^ T cell antitumor immunity by CDK4/6i treatment is related to G_1_/S transition activity in tumor cells.

### 2.5 CDK4/6i Functionally Reprograms TAMs By Activating the HIF‐1α Pathway in Tumor Cells and Enhancing the Resultant Interaction of the MIF‐CD44/CD74 Axis Between Them

**Figure 5:**
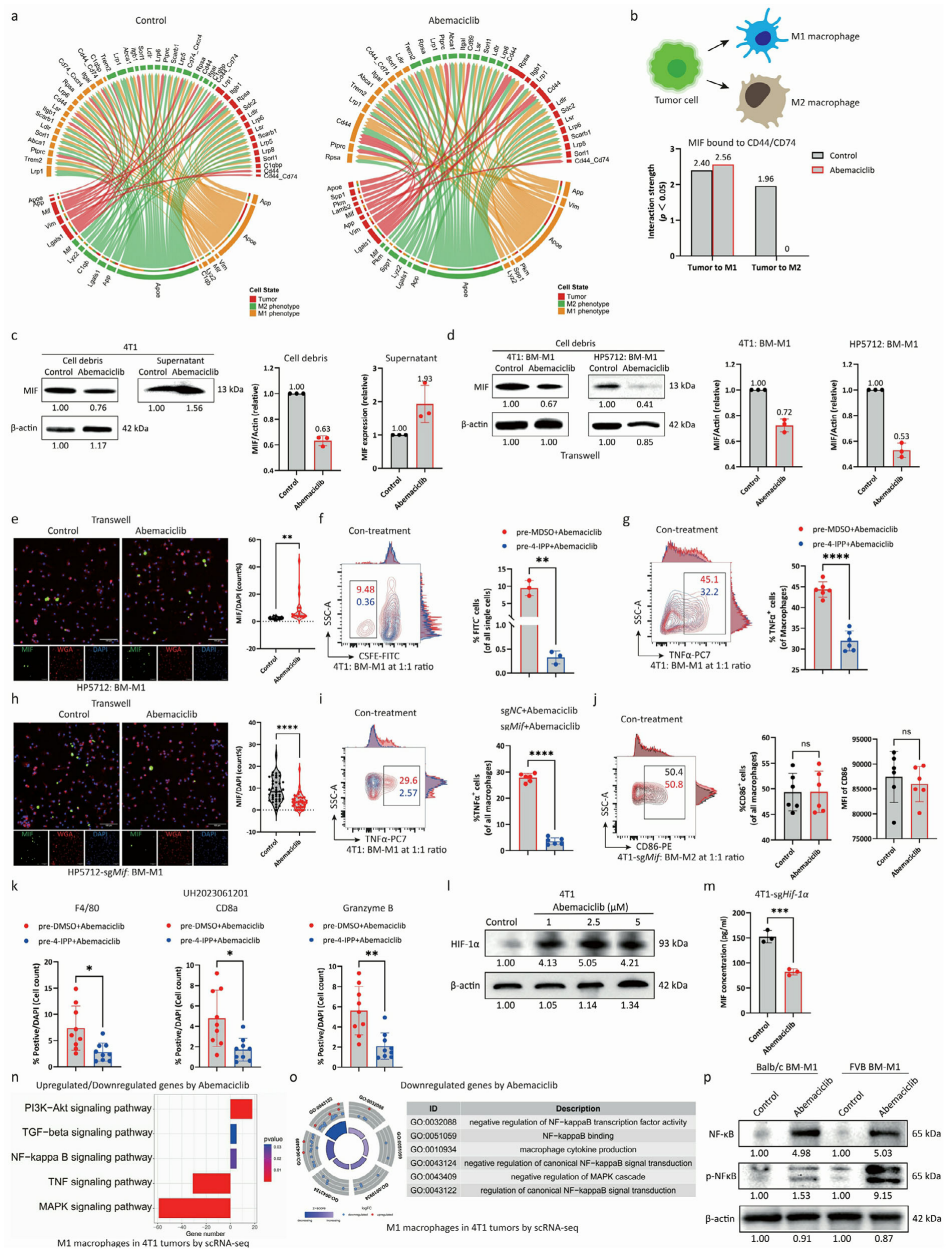
CDK4/6 inhibition triggers MIF‐induced functional reprogramming of TAMs. a,b) Cell–cell communication between tumor cells and intratumoral M1 and M2 macrophages a) and interaction strength of MIF bound to CD44/CD74 complex from tumor cells to M1 and M2 macrophages b) in Balb/c mice orthotopically implanted with 4T1 tumors after control or abemaciclib treatment. c) Representative immunoblots and fold changes in MIF expression in control‐ or abemaciclib‐treated 4T1 cell debris and supernatant. d) Representative immunoblots and fold changes in MIF expression in control‐ or abemaciclib‐treated tumor cell debris from 4T1 or HP5712:BM‐M1 macrophages in the Transwell model. e) Representative images and quantification of MIF expression (green) in control‐ or abemaciclib‐treated FVB mouse BM‐M1 macrophages from HP5712:BM‐M1 in the Transwell model (5 areas × 4 samples, for each); scale bar = 100 µm. f) Representative FACS plots and quantification of the proliferation potential of Balb/c mouse BM‐M1 macrophages cocultured with 4T1 after pre‐DMSO plus abemaciclib or pre‐4‐IPP plus abemaciclib treatment in the con‐treatment model (*n* = 3, for each). g) Representative FACS plots and quantification of TNFα expression on Balb/c mouse BM‐M1 macrophage cocultured with 4T1 after pre‐DMSO plus abemaciclib or pre‐4‐IPP plus abemaciclib treatment in the con‐treatment model (*n* = 6, for each). h) Representative images and quantification of MIF expression (green) in control‐ or abemaciclib‐treated FVB mouse BM‐M1 macrophages from HP5712‐sg*Mif*:BM‐M1 in the Transwell model (10 areas × 4 samples, for each); scale bar = 100 µm. i) Representative FACS plots and quantification of TNFα expression on Balb/c mouse BM‐M1 macrophages cocultured with 4T1‐sg*Nc* or 4T1‐sg*Mif* after abemaciclib treatment in the con‐treatment model (*n* = 6, for each). j) Representative FACS plots and quantification of CD86 expression on Balb/c mouse BM‐M2 macrophages cocultured with 4T1‐sg*Mif* after control or abemaciclib treatment in the con‐treatment model (*n* = 6, for each). k) 3D‐immunofluorescence (3D‐IF) quantification of F4/80‐positive, CD8a‐positive, and granzyme B‐positive cells in human TNBC tumor slices after pre‐DMSO plus abemaciclib or pre‐4‐IPP plus abemaciclib treatment (3 areas × 3 samples, for each). l) Representative immunoblots and quantification of HIF‐1α expression in control‐ or gradient concentrations of abemaciclib‐treated 4T1 cell debris. m) Concentration of secreted MIF in the supernatant from 4T1‐sg*Hif‐1α* cells after control or abemaciclib treatment (*n* = 3, for each). n) Kyoto encyclopedia of genes and genomes (KEGG) terms for abemaciclib‐dysregulated genes in M1 macrophages within 4T1 tumors compared with control, as determined via scRNA‐seq. o) Gene ontology (GO) terms for abemaciclib‐downregulated genes in M1 macrophages within 4T1 tumors compared with control, as determined via scRNA‐seq. p) Representative immunoblots and quantification of NF‐κB and phosphorylated NF‐κB expression in BM‐M1 macrophages from tumor cell:BM‐M1 in the Transwell model. P‐values are calculated using unpaired two‐tailed *t*‐test e–k, m). Data presented as mean ± SD. ns, *p* > 0.05; ^*^ *p* < 0.05; ^**^ *p* < 0.01; ^***^ *p* < 0.001; ^****^ *p* < 0.0001.

To understand how tumor cells regulate TAMs following CDK4/6i treatment, we analyzed cell–cell communication (CCC) between tumor cells and macrophage subpopulations after control or abemaciclib treatment. This CCC analysis revealed that the interaction strength of the MIF‐CD44/CD74 axis between tumor cells and M1 macrophages increased, whereas the interaction between tumor cells and M2 macrophages was completely abolished after abemaciclib treatment (**Figure** **5**a–b). This suggests that this axis may be an important mediator in the functional reprogramming of TAMs following CDK4/6i treatment.

In vitro, abemaciclib reduced the expression of MIF by 40% in HP5712 and 4T1 tumor cells but dramatically increased MIF secretion in HP5712 and 4T1 tumor cell‐derived supernatants (Figure 5c; Figure S10A,B, Supporting Information). In the Transwell model, MIF levels in 4T1 and HP5712 tumor cells were still reduced by 30% and 50%, respectively (Figure 5d). Notably, the MIF uptake by FVB mouse BM‐M1 macrophages cocultured with HP5712 cells was significantly increased after abemaciclib treatment (Figure 5e). These data indicate that CDK4/6i treatment promotes MIF release by tumor cells and MIF uptake by TAMs.

To investigate whether the reprogramming of TAMs by CDK4/6i requires tumor cell‐secreted MIF induction, we pretreated tumor cells with 10 µM 4‐IPP, an MIF inhibitor, according to its IC_10_ value for tumor cells and FVB mouse BM‐M1 macrophages (Figure S10C, Supporting Information). In the con‐treatment model, the proliferation potential of FVB and Balb/c mouse BM‐M1 macrophages was significantly reduced by abemaciclib after MIF inhibition in HP5712 and 4T1 tumor cells (Figure 5f; Figure S10D, Supporting Information). Abemaciclib‐amplified TNFα expression in BM‐M1 macrophages was markedly attenuated after MIF inhibition in tumor cells (Figure 5g; Figure S10E, Supporting Information). Furthermore, MIF inhibition in HP5712 tumor cells resulted in significantly lower CD86 expression in FVB mouse BM‐M2 macrophages after abemaciclib treatment (Figure S10F, Supporting Information).

To further highlight the importance of MIF induction in CDK4/6i mediated reprogramming of TAMs, we used sgRNA to knock out *Mif* in tumor cells (Figure S10G, Supporting Information). Abemaciclib significantly reduced MIF expression in FVB mouse BM‐M1 macrophages when cocultured with HP5712‐sg*Mif* cells (Figure 5h). Also, abemaciclib treatment dramatically decreased the expression of MIF in FVB mouse BM‐M1 macrophages cocultured with HP5712‐A009 cells (Figure S9K, Supporting Information). These data indicated that the CDK4/6i‐increased MIF uptake by macrophages in the TME was from the tumor cells and dependent on their G_1_/S transition activity. *Mif* deletion in 4T1 tumor cells significantly reduced abemaciclib‐enhanced TNFα expression in Balb/c mouse BM‐M1 macrophages (Figure 5i). Similarly, abemaciclib treatment significantly decreased TNFα expression in FVB mouse BM‐M1 macrophages cocultured with HP5712‐sg*Mif* tumor cells (Figure S10H, Supporting Information). Moreover, abemaciclib did not alter CD86 expression in BM‐M2 macrophages cocultured with tumor cells after *Mif* deletion (Figure 5j; Figure S10I, Supporting Information). In the Transwell model, the enhanced MPO expression in FVB mouse BM‐M1 macrophages induced by abemaciclib treatment was significantly reduced in the HP5712‐sg*Mif* group (Figure S10J, Supporting Information). Similarly, abemaciclib‐amplified CD86 expression in FVB or Balb/c mouse BM‐M2 macrophages, was significantly attenuated in the HP5712‐sg*Mif* or 4T1‐sg*Mif* group (Figure S10K,L, Supporting Information). Notably, pretreatment with 4‐IPP also significantly reduced abemaciclib‐induced increase in macrophage and CD8^+^ T cell populations, as well as granzyme B expression, in human TNBC and luminal HER2‐negative breast tumor slices (Figure 5k; Figure S10M,N, Supporting Information).

Next, we investigated how CDK4/6i promotes tumor cells to secrete the MIF cytokine. KEGG analysis of abemaciclib‐upregulated genes positively correlated with *Mif* expression in 4T1 tumor cells revealed the HIF‐1 pathway to be the top signaling pathway (Figure S11A, Supporting Information). Abemaciclib treatment markedly increased HIF‐1α expression in tumor cells, but not in a dose‐dependent manner (Figure 5l; Figure S11B, Supporting Information). We then used sgRNAs to knock out *Hif‐1α* in 4T1 tumor cells (Figure S11C, Supporting Information) and intriguingly found that abemaciclib significantly decreased MIF release by tumor cells (Figure 5m). These results indicate that CDK4/6i‐enhanced MIF secretion by tumor cells relies on the HIF‐1α pathway.

Because CD74 acts as a natural receptor for MIF on the surface of macrophages within the CD44/CD74 complex,^[^ ^37^ ^]^ we used sg*Cd74* on macrophages to block MIF‐CD44/CD74 signal transduction (Figure S11D,E, Supporting Information). Abemaciclib failed to increase TNFα expression in FVB mouse BM‐M1 macrophages and CD86 expression in FVB mouse BM‐M2 macrophages when HP5712 tumor cells were cocultured with BM‐M2‐sg*Cd74* macrophages (Figure S11F,H, Supporting Information). Similarly, abemaciclib‐amplified TNFα expression in Balb/c mouse BM‐M1 macrophages and CD86 expression in Balb/c mouse BM‐M2 macrophages were significantly reduced in the macrophage‐sg*Cd74* group (Figure S11G,I, Supporting Information). Notably, TCGA analysis showed no difference in survival between patients with *CD74*‐high and *CD7*4‐low breast cancer exhibiting high G_1_/S transition activity. However, in patients with low G_1_/S transition activity, the *CD74*‐high cohort showed significantly better survival outcomes than the *CD74*‐low cohort (Figure S11J–K, Supporting Information). Collectively, these findings highlight the crucial role of the tumor cell‐released MIF cytokine in the functional reprogramming of TAMs by CDK4/6i treatment.

MIF invigorates the NF‐κB signaling pathway,^[^ ^38^, ^39^ ^]^ which is closely related to the production of TNF.^[^ ^40^, ^41^ ^]^ We performed KEGG and GO enrichment analyses on abemaciclib‐dysregulated genes in M1 macrophages from the 4T1 tumor scRNA‐seq data. The KEGG pathway analysis revealed that these genes were enriched in the NF‐κB signaling pathway, NF‐κB signaling activation‐associated pathways, such as the PI3K‐Akt and MAPK signaling pathways, and the TNF signaling pathway (Figure 5n). The GO analysis revealed that abemaciclib‐downregulated genes were enriched in processes related to the negative regulation of NF‐κB transcription factor activity and the negative regulation of MAPK cascade (Figure 5o). Immunoblotting results further demonstrated increased NF‐κB expression and activation in BM‐M1 macrophages following abemaciclib treatment (Figure 5p). Therefore, MIF‐induced activation of the NF‐κB signaling pathway in M1 TAMs likely promotes TNFα production.

### 2.6 MIF‐CD44/CD74 Signal Blockade Mitigates CDK4/6i‐Induced TAM MHC‐I Antigen Presentation and CD8^+^ T Cell Antitumor Immunity

**Figure 6:**
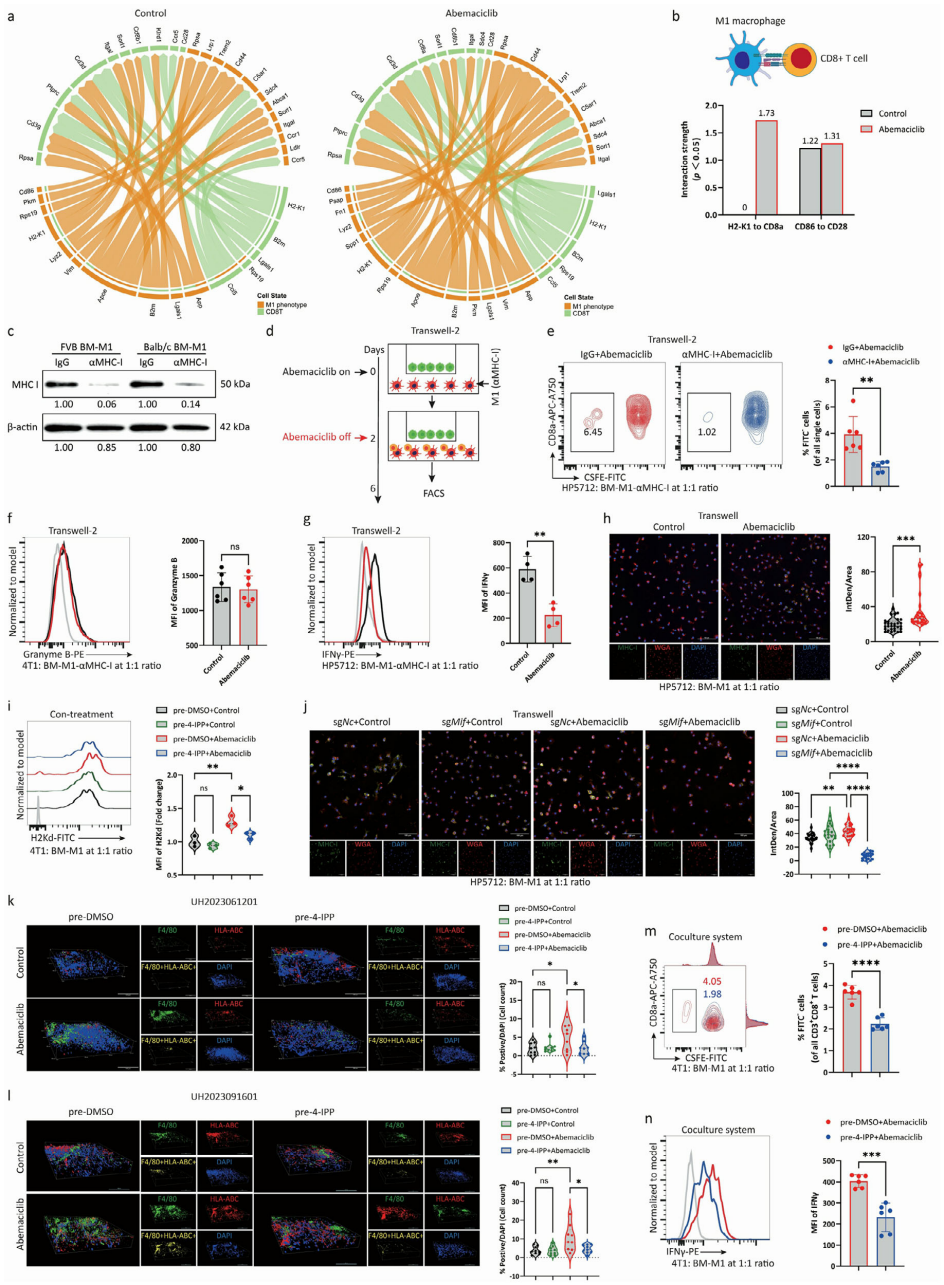
CDK4/6 inhibition activates CD8^+^ T cell antitumor immunity depending upon MIF‐induced functional reprogramming of TAMs. a,b) Cell–cell communication between M1 macrophages and CD8^+^ T cells a) and interaction strength of H2‐K1 bound to CD8a and CD86 bound to CD28 from M1 macrophages to CD8^+^ T cells b) in Balb/c mice orthotopically implanted with 4T1 tumors after control or abemaciclib treatment. c) Representative immunoblots for MHC‐I expression in FVB and Balb/c mouse BM‐M1 macrophages after IgG or anti‐MHC‐I treatment. d) The Transwell‐2 model to detect the proliferation and activation of CD8^+^ T cells. e) Representative FACS plots and quantification of the proliferation potential of FVB mouse‐derived naïve CD8^+^ T cells in the Transwell‐2 model of HP5712:FVB mouse BM‐M1 macrophages at a 1:1 ratio after pre‐IgG plus abemaciclib or pre‐anti‐MHC‐I plus abemaciclib treatment (*n* = 6, for each). f) Representative FACS histograms and quantification of granzyme B expression on the surface of Balb/c mouse‐derived activated CD8^+^ T cells in the Transwell‐2 model of 4T1:Balb/c mouse BM‐M1‐αMHC‐I macrophages at a 1:1 ratio after control or abemaciclib treatment (*n* = 6, for each). g) Representative FACS histograms and quantification of IFNγ expression on the surface of FVB mouse‐derived activated CD8^+^ T cells in the Transwell‐2 model of HP5712:FVB mouse BM‐M1 macrophages at a 1:1 ratio after pre‐IgG plus abemaciclib or pre‐anti‐MHC‐I plus abemaciclib treatment (*n* = 4, for each). h) Representative images and quantification of MHC‐I expression (green) in control‐ or abemaciclib‐treated FVB mouse BM‐M1 macrophages from HP5712:BM‐M1 in the Transwell model (10 areas × 3 samples, for each); scale bar = 100 µm. i) Representative FACS histograms and quantification of H2Kd expression on the surface of Balb/c mouse BM‐M1 macrophages cocultured with 4T1 after pre‐DMSO or pre‐4‐IPP plus control or abemaciclib treatment in the con‐treatment model (*n* = 3, for each). j) Representative images and quantification of MHC‐I expression (green) on FVB mouse BM‐M1 macrophages cocultured with HP5712‐sg*Nc* or HP5712‐sg*Mif* after control or abemaciclib treatment in the Transwell model (10 areas × 4 samples, for each); scale bar = 100 µm. k,l) Representative 3D‐immunofluorescence (3D‐IF) images and quantification of F4/80 (green) and HLA‐ABC (red) colonizing cells (yellow) in human TNBC (k; 3 areas × 3 samples, for each) and luminal breast tumor slices (l; 3 areas × 3 samples, for each) after pre‐DMSO or pre‐4‐IPP plus control or abemaciclib *ex vivo* treatment; scale bar = 100 µm. m) Representative FACS plots and quantification of the proliferation potential of Balb/c mouse‐derived naïve CD8^+^ T cells in the coculture system of 4T1:Balb/c mouse BM‐M1 macrophages at a 1:1 ratio after pre‐DMSO plus abemaciclib or pre‐4‐IPP plus abemaciclib treatment (*n* = 6, for each). n) Representative FACS histograms and quantification of IFNγ expression on the surface of Balb/c mouse‐derived activated CD8^+^ T cells in the coculture system of 4T1:Balb/c mouse BM‐M1 macrophages at a 1:1 ratio after pre‐DMSO plus abemaciclib or pre‐4‐IPP plus abemaciclib treatment (*n* = 6, for each). P‐values are calculated using unpaired two‐tailed *t*‐test e–h, m,n), or two‐way ANOVA corrected for multiple comparisons i–l). Data presented as mean ± SD. ns, *p* > 0.05; ^*^ *p* < 0.05; ^**^ *p* < 0.01; ^***^ *p* < 0.001; ^****^ *p* < 0.0001.

To determine whether CDK4/6i‐trained M1 TAMs activate CD8^+^ T cell antitumor immunity via antigen presentation, we performed CCC analysis between intratumoral M1 macrophages and CD8^+^ T cells with or without abemaciclib treatment. As expected, we found several major histocompatibility complex‐I (MHC‐I) molecules from M1 macrophages bound to CD8^+^ T cell receptors (**Figure** **6**a). In particular, the interaction strength of H2‐K1 bound to CD8a strongly increased from 0 to 1.73, and the interaction strength of CD86 bound to CD28 was also augmented in the abemaciclib group (Figure 6b). We then used an anti‐MHC‐I antibody to deplete MHC‐I expression in FVB and Balb/c mouse BM‐M1 macrophages (Figure 6c). To investigate the proliferation and activation status of CD8^+^ T cells following CDK4/6i treatment after MHC‐I depletion in TAMs, we designed the Transwell‐2 model (Figure 6d). We found that the proliferation potential of FVB mice‐derived naïve CD8^+^ T cells was significantly decreased in the MHC‐I depletion group following abemaciclib treatment (Figure 6e). After MHC‐I depletion in BM‐M1 macrophages, abemaciclib dramatically decreased the expression of IFNγ in FVB mice‐derived activated CD8^+^ T cells but failed to change granzyme B expression in Balb/c mice‐derived activated CD8^+^ T cells (Figure 6f–g). These findings indicate that MHC‐I antigen presentation is a central mediator between M1 macrophages and CD8^+^ T cells following CDK4/6i treatment.

To understand whether tumor cell‐derived MIF induction in M1 TAMs controls MHC‐I antigen presentation between M1 phenotypes and CD8^+^ T cells after CDK4/6i treatment, we directly treated Balb/c and FVB mouse BM‐M1 macrophages with abemaciclib. We did not find any changes in H2Kd in Balb/c mouse BM‐M1 macrophages or in MHC‐I expression in FVB mouse BM‐M1 macrophages (Figure S12A,B, Supporting Information). However, abemaciclib significantly enhanced MHC‐I expression in FVB mouse BM‐M1 macrophages cocultured with HP5712 cells in the Transwell model (Figure 6h). Next, we pretreated 4T1 tumor cells with 4‐IPP in the con‐treatment model and found that abemaciclib‐amplified H2Kd expression in Balb/c mouse BM‐M1 macrophages was significantly reduced by MIF inhibition (Figure 6i). Similarly, 4‐IPP pretreatment of 4T1 tumor cells dramatically reduced abemaciclib‐amplified H2Kd expression in Balb/c mouse BM‐M1 macrophages in the Transwell model (Figure S12C, Supporting Information).

When *Mif* was knocked out in HP5712 cells, abemaciclib remarkably attenuated MHC‐I expression in FVB mouse BM‐M1 macrophages in the Transwell model; the abemaciclib‐enhanced MHC‐I expression in these macrophages was significantly decreased following *Mif* deletion in tumor cells (Figure 6j). Notably, abemaciclib‐amplified HLA‐ABC expression on TAMs in human TNBC and luminal HER2‐negative breast tumors was dramatically reduced by 4‐IPP pretreatment (Figure 6k,l). Significantly lower H2Kd expression was also observed in Balb/c mouse BM‐M1‐sg*Cd74* macrophages cocultured with 4T1 tumor cells after abemaciclib treatment (Figure S12D, Supporting Information), indicating that the MIF‐CD74 axis activity between tumor cells and M1 macrophages dominates CDK4/6i‐enhanced MHC‐I antigen presentation between M1 macrophages and CD8^+^ T cells. Additionally, abemaciclib strongly reduced the expression of MHC‐I in FVB mouse BM‐M1 macrophages cocultured with HP5712‐A009 cells in the Transwell model (Figure S9L, Supporting Information), highlighting the important role played by the G_1_/S transition activity in tumor cells by CDK4/6i‐amplified MHC‐I expression in TAMs.

Based on the abovementioned findings, we hypothesized that blocking MIF induction in TAMs might interfere with CDK4/6i‐enhanced proliferation and activation of CD8^+^ T cells. Indeed, 4‐IPP pretreatment of HP5712 and 4T1 tumor cells significantly ablated the abemaciclib‐amplified proliferation of FVB and Balb/c mice‐derived naïve CD8^+^ T cells and the abemaciclib‐amplified IFNγ expression in activated CD8^+^ T cells (Figure 6m,n; Figure S12E,F, Supporting Information). When HP5712 cells were cocultured with FVB mouse BM‐M1‐sg*Cd74* macrophages, abemaciclib did not increase the proliferation potential of FVB mice‐derived naïve CD8^+^ T cells or IFNγ expression in activated CD8^+^ T cells (Figure S12G,H, Supporting Information). These data indicate that MIF‐induced M1 TAMs are key mediators of CDK4/6i‐triggered CD8^+^ T cell antitumor immunity.

### 2.7 CDK4/6i‐Trained M1 TAM Supernatant Provokes the Tumor Response of Low‐Dose PD‐1 ICB Therapy

**Figure 7:**
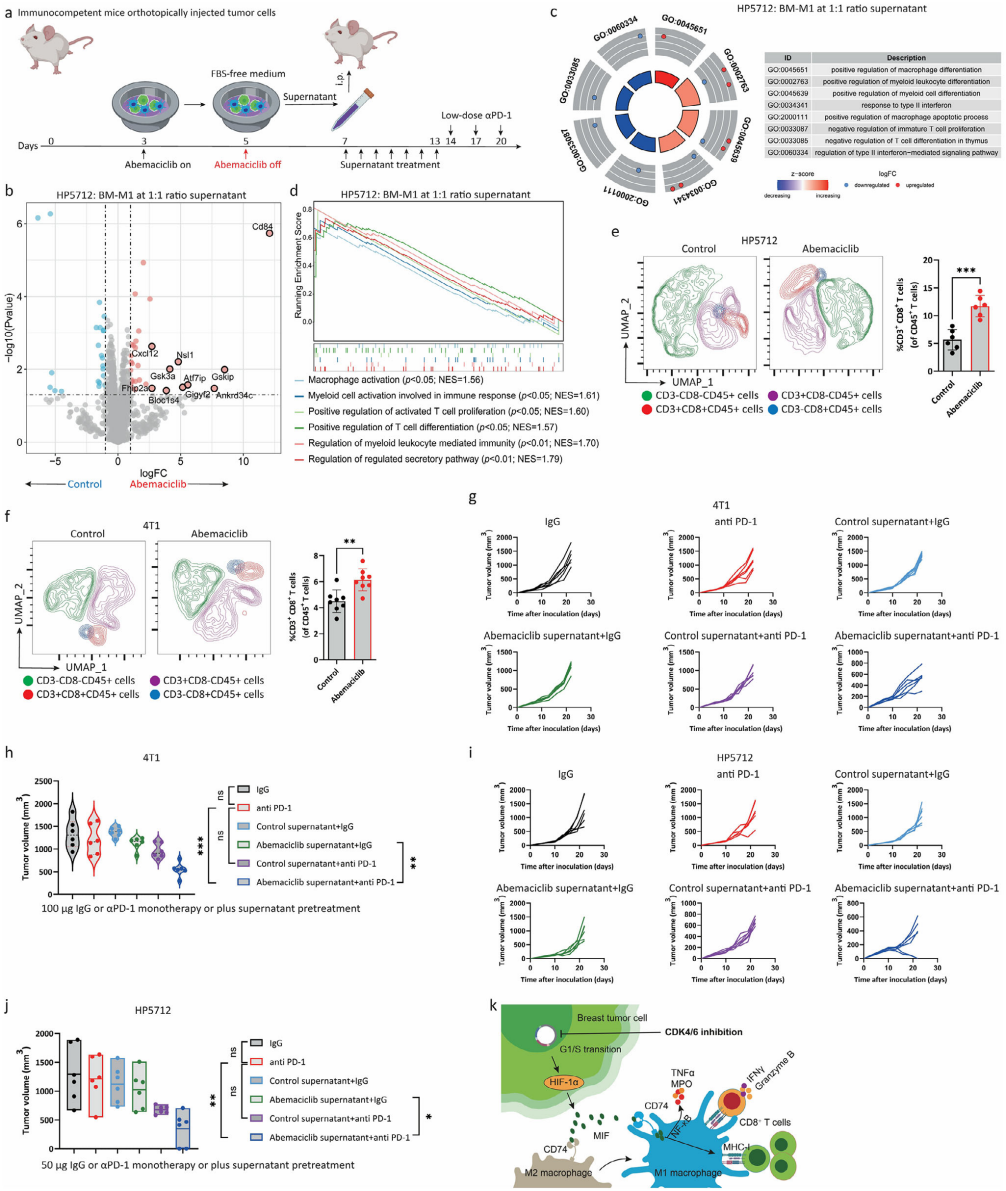
CDK4/6 inhibition‐trained M1 TAM supernatant pretreatment potentiates the antitumor activity of low‐dose PD‐1 ICB therapy. a) Workflow for CDK4/6 inhibition‐trained M1 TAM supernatant pretreatment combined with low‐dose PD‐1 ICB therapy in vivo. b) Volcano plot depicting the full protein composition in CDK4/6 inhibition‐trained M1 TAM supernatant. c,d) Gene ontology (GO) terms for abemaciclib significantly upregulated and downregulated proteomics b) and gene set enrichment analysis (GSEA) terms for abemaciclib significantly upregulated proteomics c) in supernatants from HP‐5712:FVB mouse BM‐M1 macrophages at a 1:1 ratio (*n* = 3, for each). e,f) Representative uniform manifold approximation and projections (UMAPs) and quantification of intratumoral CD8^+^ T cells among all lymphocytes in immunocompetent mice orthotopically implanted with HP5712 tumors (d; *n* = 6, for each) or 4T1 tumors (e; *n* = 6, for each) after control‐ or abemaciclib‐trained M1 TAM supernatant treatment. g,h) Changes in tumor volume f) and tumor volume comparison g) for Balb/c mice orthotopically implanted with 4T1 tumors after low‐dose IgG or PD‐1 ICB monotherapy or therapy combined with control supernatant or abemaciclib supernatant pretreatment (*n* = 6, for each). i,j) Changes in tumor volume h) and tumor volume comparison i) for FVB mice orthotopically implanted with HP5712 tumors after low‐dose IgG or PD‐1 ICB monotherapy or therapy combined with control supernatant or abemaciclib supernatant pretreatment (*n* = 6, for each). k) Schematic of the mechanisms. P‐values are calculated using Unpaired two‐tailed *t*‐test d,e), or one‐way ANOVA corrected for multiple comparisons g,i). Data presented as mean ± SD. ns, *p* > 0.05; ^*^ *p* < 0.05; ^**^ *p* < 0.01; ^***^ *p* < 0.001; ^****^ *p* < 0.0001.

A phase Ib clinical trial, with co‐administration of abemaciclib and pembrolizumab for patients with hormone receptor‐positive, HER2‐negative metastatic breast cancer, revealed an antitumor immunity but more frequent grade ≥3 interstitial lung disease/pneumonitis and severe transaminase elevation than abemaciclib or pembrolizumab monotherapy.^[^ ^42^ ^]^ Consequently, the evaluation of this combination was terminated for these patients. To mitigate the high frequency of adverse events associated with this combination therapy, we used CDK4/6i inhibitor‐trained M1 TAM supernatant pretreatment to activate the tumor response to low‐dose PD‐1 ICB therapy in murine breast tumor xenografts (**Figure** **7**a). Abemaciclib supernatant therapy resulted in an enhanced tumor volume reduction in immunocompetent mice implanted with HP5712 and 4T1 tumors compared with the control supernatant therapy (Figure S13A, Supporting Information).

To corroborate the antitumor immunity of the CDK4/6i treatment supernatant therapy, we performed a proteomic analysis for pretreatment with supernatant from HP5712 cells and FVB mouse BM‐M1 macrophages at a 1:1 ratio after control or abemaciclib treatment. Abemaciclib upregulated several proteins, including CD84, NSL1, GSKIP, ANKRD34C, ATF7IP, CXCL12, GSK3A, FHIP2A, BLOC1S4, and GIGYF2 (Figure 7b). The GO terms for these proteins were enriched in pathways related to “positive regulation of macrophage differentiation,” “positive regulation of myeloid cell differentiation,” and “response to type II interferon,” whereas abemaciclib‐downregulated proteins were enriched for “positive regulation of macrophage apoptotic process,” “negative regulation of immature T cell proliferation,” and “negative regulation of T cell differentiation in the thymus” (Figure 7c). The abemaciclib‐upregulated proteins were enriched for similar GSEA terms, including “macrophage activation,” “positive regulation of activated T cell proliferation,” “positive regulation of T cell differentiation,” “regulation of myeloid leukocyte‐mediated immunity,” and “regulation of regulated secretory pathway” (Figure 7d). Additionally, after one week of continuous abemaciclib‐trained M1 TAM supernatant therapy, CD8^+^ T cells in HP5712 and 4T1 tumors were significantly increased on day 8 (Figure 7e,f). Therefore, CDK4/6i‐trained M1 TAM supernatant therapy resulted in an amplified CD8^+^ T cell antitumor immunity.

Next, we aimed to determine whether CDK4/6i‐trained M1 TAM supernatant therapy could enhance the antitumor activity of low‐dose PD‐1 ICB therapy in breast cancers. In Balb/c mice orthotopically implanted with 4T1 tumors, low‐dose PD‐1 ICB therapy did not elicit a tumor response, and control‐trained M1 TAM supernatant pretreatment failed to spur it as well. In contrast, abemaciclib‐trained M1 TAM supernatant pretreatment significantly augmented the antitumor activity of low‐dose PD‐1 ICB therapy. The combination therapy of abemaciclib‐trained M1 TAM supernatant and PD‐1 ICB also displayed markedly stronger tumor reduction than the abemaciclib‐trained M1 TAM supernatant monotherapy (Figure 7g,h). This combination strategy potently increased the tumor‐killing effects compared with PD‐1 ICB or abemaciclib‐trained M1 TAM supernatant monotherapy in Balb/c mice implanted with 4T1 tumor slices (Figure S13B,C, Supporting Information). Moreover, low‐dose PD‐1 ICB monotherapy showed no tumor reduction in FVB mice orthotopically implanted with HP5712 tumors, and its combination with control‐trained M1 TAM supernatant pretreatment did not achieve significantly better control of tumor volume than achieved with PD‐1 ICB alone. In contrast, the combination of abemaciclib‐trained M1 TAM supernatant pretreatment and PD‐1 ICB therapy exhibited a dramatic reduction in tumor volume compared with either PD‐1 ICB alone or abemaciclib‐trained M1 TAM supernatant monotherapy (Figure 7i,j). Notably, two out of the six mice in this combination group were found to be tumor‐free (Figure 7i–j).

To support the application of CDK4/6i‐trained M1 TAM supernatant therapy in breast cancer, we further implanted HP5712 tumor cells in FVB mice prior to harvesting BM‐M1 macrophages. Abemaciclib‐trained M1 TAM supernatant therapy using tumor‐bearing FVB mouse BM‐M1 macrophages not only increased the population of CD8^+^ T cells but also amplified IFNγ expression in all CD8^+^ T cells in HP5712 tumors (Figure S13D,E, Supporting Information). Moreover, abemaciclib‐trained M1 TAM supernatant therapy significantly reduced the tumor volume (Figure S13F, Supporting Information).

Next, we evaluated the translation of the antitumor activity benefits of CDK4/6i‐trained M1 TAM supernatant and PD‐1 ICB combination therapy to human breast cancers. We first investigated the optimal concentrations of pembrolizumab on human TNBC and luminal HER2‐ breast tumor samples using 3D‐TSC models. Human TNBC and luminal HER2‐ breast cancer tumors began to respond to pembrolizumab at 50 and 12.5 µg mL^−1^, respectively (Figure S13G, Supporting Information). Therefore, we applied 25 and 6.25 µg/mL pembrolizumab in combination with supernatant pretreatment for human TNBC and Luminal HER2‐ breast tumor slices, respectively. To broaden the sources of supernatant beyond patient‐specific donors, we utilized MDA‐MB‐231 cells cocultured with THP‐1 M1 macrophages as a model for mimicking CDK4/6i‐trained M1 TAMs. Low‐dose pembrolizumab alone did not enhance tumor‐killing in either type of human breast tumor slices compared with IgG1 alone. In contrast, the combination of pembrolizumab with abemaciclib‐trained M1 TAM supernatant pretreatment, but not control‐trained M1 TAM supernatant pretreatment, exhibited significantly higher tumor‐killing effects than low‐dose pembrolizumab monotherapy (Figure S13H,I, Supporting Information). Collectively, CDK4/6i‐trained M1 TAM supernatant therapy enhanced the antitumor activity of low‐dose PD‐1 ICB therapy in breast tumors *ex vivo*.

Finally, TCGA overall survival data support the notion that multiple cancer types—including breast cancer, head and neck squamous cell carcinoma, sarcoma, skin cutaneous melanoma, and uterine corpus endometrial carcinoma—may benefit from activated CD8^+^ T cell‐mediated antitumor immunity (Figure S13J–N, Supporting Information).

## 3 Discussion

Herein, we provide mechanistic insights into how CDK4/6i activates CD8^+^ T cell antitumor immunity in breast tumors through the functional reprogramming of TAMs (Figure 7k). Moreover, we show that CDK4/6i‐trained M1 TAM supernatant therapy represents a promising strategy to markedly potentiate the antitumor activity of PD‐1 ICB therapy.

The impact of CDK4/6i treatment in promoting CD8^+^ T cell antitumor immunity has been elucidated recently.^[^ ^5^, ^8^, ^33^ ^]^ In a pioneering study, Goel et al. proposed two key mechanisms by which CDK4/6i overcomes tumor immune evasion—increased tumor cell antigen presentation and reduced immunosuppressive Treg populations.^[^ ^6^ ^]^ Consistently, our scRNA‐seq analysis also revealed attenuated Tregs in 4T1 tumors after abemaciclib treatment. CDK4/6i treatment also enhanced the infiltration of CD8^+^ T cells and B cells, as well as myeloid cell activation.^[^ ^8^, ^16^ ^]^ Our findings further highlight the important role of CDK4/6i in activating TAMs. However, the cell–cell interactions between lymphoid cells and non‐lymphoid cells, especially tumor cells, myeloid cells, and T cell immunoregulatory circuit, remain unclear. The orchestration of TAMs within the tumor cell–macrophage–CD8^+^ T cell loop plays a crucial role in modulating cancer immunotherapy responses.^[^ ^43^ ^]^ Using scRNA‐seq analysis, we confirmed that CDK4/6i‐amplified CD8^+^ T cells are mediated by M1 TAMs. Additionally, abemaciclib treatment in B6 (C2J)/LysM‐GFP mice orthotopically implanted with Py8119 tumors and normal organs specifically confirms the immune‐like macrophage memory. This phenotype clarifies why, after removal of abemaciclib, TAMs still activate CD8^+^ T cell antitumor immunity and explains CDK4/6i‐induced immunological T‐cell memory.^[^ ^44^ ^]^

T cells are recognized as the initial hosts to generate MIF, thus defining the proinflammatory nature of this cytokine for innate and adaptive immunity.^[^ ^45^, ^46^ ^]^ Normal tissues and multiple immune cell types have been found to express MIF.^[^ ^34^, ^35^ ^]^ Notably, MIF is overexpressed by primary tumors and tumor cell lines,^[^ ^47^, ^48^, ^49^ ^]^ and it negatively regulates p53‐controlled cell growth arrest and apoptosis,^[^ ^50^ ^]^ highlighting its significant role in oncogenesis and tumor progression.^[^ ^51^ ^]^ A large body of evidence shows that the use of *Mif* knockdown or knockout in cancer cells improves the M1 TAM‐ and CD8^+^ T cell‐associated antitumor immunity.^[^ ^52^, ^53^ ^]^ The overall immunological outcome therefore, depends on the relative abundance of MIF sources, the receptor landscape (e.g., CD74 versus CXCR4 engagement), and treatment‐induced shifts in the tumor microenvironment. In metastatic melanoma, blockade of the MIF–CD74 axis on macrophages and dendritic cells reprograms TAMs toward an M1 phenotype^[^ ^54^ ^]^, while in cisplatin‐resistant lung cancer, inhibition of the Src/CD155/MIF pathway elicits a similar M1 bias.^[^ ^55^ ^]^ Conversely, in vitro studies have shown that exogenous supplementation of MIF to BMDMs dose‐dependently upregulates M1 markers such as iNOS and TNF‐α, thereby promoting M1 polarization^[^ ^54^ ^]^; additionally, under conditions of high endogenous MIF expression, macrophage‐autonomous MIF enhances iNOS and IL‐18 levels to drive M1 differentiation^[^ ^55^ ^]^; and in an aortic aneurysm model, MIF activates the JNK/c‐Jun pathway via CD74/CXCR2 to markedly increase CD86 and IL‐18 expression, likewise fostering M1 polarization.^[^ ^56^ ^]^ Our data further demonstrate that CDK4/6 inhibition not only downregulates tumor‐cell MIF—effectively recapitulating the effects of MIF knockout—but also promotes MIF accumulation within TAMs, thereby robustly expanding M1 TAMs and activating CD8⁺ T cells. Therefore, the role of MIF in macrophage polarization may be context‐dependent.

In response to extracellular stimuli, cells can rapidly release MIF to increase its circulating concentration.^[^ ^34^, ^35^ ^]^ Stimulation of the proinflammatory cytokines, TNFα and IFNγ, in macrophages, enhances MIF secretion.^[^ ^57^ ^]^ We demonstrate that CDK4/6i‐induced MIF induction in TAMs broadens TNFα release and increases the populations of antitumoral macrophages, characterized by high MPO levels that are positively related to optimal survival.^[^ ^36^ ^]^ Additionally, loss of *Mif* or treatment with the MIF inhibitor 4‐IPP in solid tumors interferes with the immunosuppressive or immunostimulatory phenotype of TAMs.^[^ ^58^ ^]^ In our study, CDK4/6i treatment in tumor cells resulted in low intracellular MIF expression and high extracellular MIF cytokine concentration, promoting MIF uptake by macrophages. This process induced the polarization of TAMs from the M2 to the M1 phenotype. Loss of *Mif* or 4‐IPP treatment in tumor cells, or alternatively loss of *Cd74* in macrophages, halted the CDK4/6i‐induced polarization. Our data provide compelling evidence that CDK4/6i treatment increases the MIF uptake by TAMs from tumor cells. When we used *Mif* deletion in tumor cells or CDK4/6i drug‐resistant cells, abemaciclib remarkably decreased the expression of MIF in TAMs. Potential acquired resistance mechanisms against CDK4/6i reflect both genetic (mutations and amplifications)^[^ ^3^, ^59^, ^60^ ^]^ and adaptive (signaling and metabolic, immune)^[^ ^61^, ^62^ ^]^ changes. MIF signaling intertwines with pathways implicated in CDK4/6i resistance (e.g., PI3K/Akt/mTOR and MAPK/ERK), potentially amplifying compensatory survival mechanisms after inhibitor exposure.^[^ ^3^, ^60^, ^61^ ^]^ Therefore, promoting MIF release by tumor cells and MIF uptake by macrophages is a critical pathway for the functional reprogramming of TAMs.

Inhibition of G_1_/S transition in tumor cells can cause the accumulation of DNA damage and alter cellular metabolism, which blocks lysosomal degradation of HIF‐1α and increases HIF‐1α stabilization.^[^ ^63^, ^64^ ^]^ Our study consistently demonstrates that CDK4/6i invigorates the HIF‐1α pathway in breast tumor cells. HIF‐1α activation facilitates glycolysis, which polarizes macrophages to the M1 phenotype but not to the M2 phenotype.^[^ ^65^ ^]^ A previous study has confirmed that HIF1α, but not HIF2α, drives MIF secretion under hypoxic conditions in acute myeloid leukemia blasts.^[^ ^66^ ^]^ In breast cancer cells, we found that MIF release was also related to the HIF‐1α pathway in response to CDK4/6i treatment.

TAMs predominantly regulate T‐cell functionality via direct antigen presentation or indirectly via soluble factors within the TME. In response to the uptake of phagocytic bodies, antigens, or cytokines released by tumor cells, TAMs rapidly present neoantigens on their cell surface or release molecules out of their membrane.^[^ ^28^ ^]^ For example, TAMs interact with the T‐cell receptor of CD4^+^ T cells via MHC‐II‐mediated antigen presentation.^[^ ^28^ ^]^ Immunosuppressive TAMs produce several molecules, including PD‐L1, PD‐L2, IL‐10, TGF‐β, and arginase, to trigger T‐cell dysfunction.^[^ ^67^, ^68^, ^69^ ^]^ Our study highlights that CDK4/6i treatment triggers the immunostimulatory status of TAMs via the uptake of MIF cytokine, which consequently reinvigorates intratumoral CD8^+^ T cell antitumor activity that is dependent on MHC‐I‐mediated antigen presentation. Thus, strategies to specifically enhance MHC‐I antigen presentation by TAMs can overcome immune evasion in solid tumors. Notably, the CDK4/6i‐amplified MHC‐I expression in TAMs is associated with MIF uptake. This is evidenced by the strong decrease in MHC‐I expression on the surface of TAMs when *Mif*‐deleted tumor cells were used in the coculture model after abemaciclib treatment.

Macrophages are abundant in the TME and effectively penetrate the tumors. This provides a proof‐of‐principle for macrophage immunotherapy for solid tumors, such as breast, lung, and bladder cancers. Indeed, chimeric antigen receptor macrophages (CAR‐M) show better tumor infiltration than CAR‐T and CAR‐NK cells.^[^ ^45^, ^46^ ^]^ Additionally, CAR‐M increases the infiltration of T and NK cells, as well as the activation of dendritic and CD8^+^ T cells. These benefits have led to the initiation of a phase 1 clinical trial of CAR‐M in human solid tumors (ClinicalTrials.gov # NCT04660929). However, several factors limit the efficacy of this novel strategy, such as the loss of antigen presentation on tumors, CAR downregulation, and systemic cytotoxicity,^[^ ^70^ ^]^ similar to that in the CAR‐T and CAR‐NK therapies. CAR‐M‐derived supernatant is very potent in inducing an immunostimulatory pattern from M2 macrophages.^[^ ^71^ ^]^ Therefore, we harnessed CDK4/6i‐trained M1 TAM supernatant to treat murine breast tumors and confirmed the amplified CD8^+^ T cell antitumor immunity and antitumor activity. The supernatant treatment might overcome the limitations of CAR‐M as the antigen expression on tumor cells is not required. Notably, CAR‐M and supernatant therapy alone are insufficient to eliminate tumors, and combining them with PD‐1 ICB therapy, maximizes macrophage immunotherapy.^[^ ^71^ ^]^ Thus, the combination of CDK4/6i‐trained M1 TAM supernatant pretreatment and low‐dose PD‐1 ICB therapy can overcome the limitations of CAR‐M therapy and may avoid the adverse effects of CDK4/6i treatment in conjunction with PD‐1 ICB therapy. Moreover, by using human cell lines instead of patient‐specific donors, the CDK4/6i‐trained M1 TAM supernatant is robust and reproducible.

In this study, we uncovered the mechanisms by which CDK4/6 inhibition triggers CD8^+^ T cell antitumor immunity via the functional reprogramming of TAMs. We also validated that the CDK4/6i treatment promotes MIF release by tumor cells and that this MIF cytokine induces the immunostimulatory status of TAMs, although the details of this regulation warrant further studies. A phase 1, first‐in‐human, open‐label clinical trial is currently underway to investigate the efficacy and safety of CAR‐M in patients with HER2‐overexpressing solid tumors (NCT04660929). The safety of CDK4/6i‐trained TAM supernatant therapy, alone or in combination with low‐dose PD‐1 ICB therapy, remains to be studied, although no deaths have been observed during treatment.

This study had some limitations. The major concern relates to its moderate impact, as we attempted to investigate the effect of CDK4/6i in TNBC, an immunohistotype characterized by high mitotic indices and proliferation rates that are often less dependent on the CDK4/6‐RB pathway. Furthermore, TNBC tumors frequently exhibit *RB* loss or inactivation, which could limit the effectiveness of CDK4/6i. Additionally, we proposed combining CDK4/6i‐trained M1 tumor‐associated macrophage supernatant with the anti‐PD‐1 therapy in TNBC—a novel but currently distant approach from clinical applicability.

## 4 Conclusion

Overall, our findings confirm that M1 TAMs are the primary mediators of CDK4/6in‐amplified CD8^+^ T cell antitumor immunity in breast cancers. CDK4/6i treatment reprograms TAMs to an immunostimulatory status via tumor cell‐derived MIF induction. These immunostimulant macrophages regulate the proliferation and activation of CD8^+^ T cells via MHC‐I‐mediated antigen presentation. The combination of CDK4/6i‐trained M1 TAM supernatant pretreatment and PD‐1 ICB therapy offers a promising treatment paradigm for breast tumors.

## 5 Experimental Section

### Cell Lines

4T1 (CVCL_0125), THP‐1 (CVCL_0006), MDA‐MB‐231 (CVCL_0062), Py8119 (CVCL_AQ09), RAW264.7 (CVCL_0493), and HEK293T (CVCL_0063) cells were obtained from the American type culture collection. The HP5712 cell line, a breast cancer model derived from FVB mice, was developed by Prof. Chu‐xia Deng's laboratory for preclinical oncology research. This non‐commercial resource stably recapitulates mammary tumorigenesis in syngeneic FVB mice, although it currently lacks a Research Resource Identifier (RRID) because of its academic origin. All cell lines were subjected to short tandem repeat analysis and mycoplasma testing to confirm the absence of contamination. These cells were maintained in a humidified incubator at 37 °C with 5% CO_2_. HP5712, HEK293T, Py8119, MDA‐MB‐231, and RAW264.7 cells were cultured in Dulbecco's modified Eagle medium (DMEM; Gibco; 11965092) supplemented with 10% fetal bovine serum (FBS; Gibco; A5256701) and 100 IU mL^−1^ penicillin/streptomycin (Gibco 15140122). 4T1 cells were cultured in RPMI 1640 (Gibco; 11875093) with 10% FBS and 100IU mL^−1^ penicillin/streptomycin. THP‐1 cells were cultured in RPMI 1640 with 10% FBS, 100IU mL^−1^ penicillin/streptomycin, and 0.05 mm β‐mercaptoethanol (Sigma; M3148).

### In Vivo Mouse Models

FVB, Balb/c, nude, and B6 (C2J)/LysM‐GFP mice (5–6‐weeks‐old) were supplied by the Animal Research Core at the Faculty of Health and Sciences, University of Macau. Only female mice were used in the experiments. A total of 3 × 10^5^ tumor cells were orthotopically injected into the fifth pair of mammary glands. All mouse experiments were conducted in accordance with the ethical guidelines of the University of Macau, under approval number UMAEC‐050‐2015.

### Human Breast Cancer Samples

All human studies were approved by the Institutional Review Board (reference number UH/CREC/2023/01) and reviewed by the Scientific Research Ethics Committee of the University Hospital, Macau University of Science and Technology. Two breast tumor samples were included in this study. The clinicopathological information is provided in Table S1 (Supporting Information). Human breast cancer samples were prepared as tumor slices, which were evenly placed into frozen tubes (12 pieces per tube) and cryopreserved in liquid nitrogen using 1.5 mL of cryopreservation medium (90% FBS and 10% dimethyl sulfoxide (DMSO; Sigma; 472301)).

### 3D‐Tumor Slice Culture

Immunocompetent mice with orthotopically implanted with breast tumors or surgically removed samples from patients with breast cancer were harvested for the preparation of 3D‐tumor slice cultures, as described in our previous studies.^[^ ^31^, ^32^ ^]^ Briefly, tumors were cut into uniform shapes using a round pipe instrument or scalpel and then coated with UltraPure low‐melting‐point agarose (ThermoFisher). After solidifying for 10 min at 4 °C, the agarose‐encased uniform tumor was placed into a condensation chamber filled with phosphate‐buffered saline (PBS) containing 2% penicillin/streptomycin. Dozens of thick tumor slices (300 µm) were harvested using a vibratome Leica VT1200 S (Leica Biosystems Nussloch GmbH, Germany). Rat Collagen I (R&D systems 3447‐020‐01), 10× Ham's F‐12 (Gibco 21700075), and C buffer**—**sterile reconstitution buffer, 2.2 g NaHCO_3_ (Sigma; S5761) in 100 mL of 0.05 N NaOH (Sigma; 655104) and 200 mm HEPES (Sigma; H3375), preserved at 4 °C**—**were mixed in an 8:1:1 ratio to prepare the collagen buffer. Tumor slices were coated with a lower and an upper layer of collagen buffer (150 and 50 µL, respectively) in a 3 µm‐diameter insert in a 24‐well plate (BIOFIL; TCS031024). The plate was maintained in a humidified incubator at 37 °C with 5% CO_2_ until the collagen buffer solidified completely. Next, 330 µL of culture medium**—**advanced DMEM/F‐12 (Gibco 11320033) supplemented with 20% FBS, 1% penicillin/streptomycin, and 50 µg mL^−1^ gentamycin (Gibco 15710064)**—**was added outside the insert. The 24‐well plate was maintained in a humidified incubator at 37 °C with 5% CO_2_, and the culture medium was refreshed as per the experimental design until the slices were harvested for subsequent experiments.

### Isolation of Naïve CD8^+^ T cells and Macrophages—Isolation of Naïve CD8^+^ T Cells

The spleens of immunocompetent mice were dissociated into single‐cell suspensions. After incubation with RBC lysis buffer (eBioscience 00‐4333‐57), naïve CD8^+^ T cells were isolated using the EasySep™ Mouse Naïve CD8^+^ T Cell Isolation Kit (Stemcell; 19858). To obtain activated CD8^+^ T cells, 3 µg/mL CD3 primary antibody (eBioscience; 16‐0032‐82) and 5 µg mL^−1^ CD28 primary antibody (eBioscience; 16‐0281‐82) in PBS were added to a 24‐well plate and then incubated overnight at 4 °C. The isolated naïve CD8^+^ T cells were then added to the antibody‐coated plate and incubated at 37 °C for 3 days to achieve activation. The isolated CD8^+^ T cells were cultured in RPMI 1640 supplemented with 10% FBS, 50 µm β‐mercaptoethanol, 1 mm sodium pyruvate (Sigma P5280), 10 mm HEPES, and 100 IU mL^−1^ penicillin/streptomycin.

### Isolation of Macrophages

Bone marrow was collected from the femurs of immunocompetent mice and filtered through a 40 µm cell sieve to obtain a single‐cell suspension. After 3 min of incubation with RBC lysis buffer and a subsequent PBS wash, monocytes were isolated. The monocytes were cultured in RPMI 1640 supplemented with 10% FBS, 100 IU mL^−1^ penicillin/streptomycin, and either 20 ng mL^−1^ GM‐CSF (PeproTech; 315‐03) or 20 ng/mL M‐CSF (PeproTech; 315‐02) for 4–5 days to allow differentiation into M1 or M2 macrophages, respectively. The mature macrophages were then used for downstream applications.

### In Vitro Drug Treatment

Abemaciclib (MCE HY‐16297A) and palbociclib (Selleckchem S1579) were diluted in DMSO as described previously.^[^ ^72^ ^]^ For in vitro cellular experiments, 1 µM abemaciclib was used. For *ex vivo* tumor slice experiments, 3 µM abemaciclib was used. For the synergy assay of CDK4/6 inhibitor‐trained TAM supernatant therapy and PD‐1 ICB therapy, tumor cells and macrophages were pretreated with 1 µM abemaciclib for 48 h in a con‐treatment model to generate conditioned supernatants. These supernatants, containing secreted factors from abemaciclib‐trained TAMs, were subsequently applied together with PD‐1 ICB therapy to mouse model‐ and patient‐derived tumor slices to evaluate potential synergistic antitumor effects. The tumor slices were treated with one‐quarter volume of the supernatant for 3 days; the supernatant culture medium was exchanged daily, followed by 2 days of PD‐1 ICB treatment. Mouse model‐derived tumor slices were treated with 2.5 µg mL^−1^ αPD‐1 antibody (BIOXcell; BE0146), and human breast tumor slices were treated with a specific concentration of pembrolizumab (described in Figure S15, Supporting Information) (Selleckchem; A2005) or isotype control (Sigma; I5154).

### In Vivo Drug Treatment

Abemaciclib (75 mg kg^−1^) was administered daily via oral gavage, starting on day 7, for a total of 10 days, as described previously.^[^ ^72^ ^]^ In vivo CD8^+^ T cell depletion was performed as described previously. Briefly, 200 µg of either αCD8 antibody (BIOXcell; BE0061) or 200 µg of isotype control (BIOXcell; BE0086) were intraperitoneally injected into mice 48 and 24 h before abemaciclib administration and every 4 days thereafter. For macrophage depletion experiments, either 200 µL of clodrosome or 200 µL of encapsome (SKU; CLD‐8901) were intraperitoneally injected on days 7 and 14 after orthotopic implantation of tumor cells. For the supernatant combined with PD‐1 ICB therapy, 250 µL of supernatant was intraperitoneally injected, starting on day 7, and continued daily for one week, followed by three intraperitoneal injections, starting on day 14, every three days of either αPD‐1 antibody (50 µg for FVB mice bearing HP5712 tumors; 100 µg for Balb/c mice bearing 4T1 tumors) or isotype control treatment at the corresponding doses.

### *Ex Vivo* and In Vitro CD8 Depletion, MHC‐I Depletion, and Macrophage Depletion

For *ex vivo* CD8 depletion on tumor slices, 10 µg mL^−1^ αCD8 antibody or isotype control was used for 3 days. For macrophage depletion on tumor slices, 0.25 mg mL^−1^ clodrosome or encapsome was used for 3 days. For in vitro depletion, 10 µg mL^−1^ αCD8 antibody, 10 µg mL^−1^ αMHC‐I antibody (BIOXcell; BE0172 and BE0104, respectively), or isotype control was used for 3 days on isolated CD8^+^ T cells and bone marrow‐derived macrophages, respectively.

### Immunofluorescence (IF)

Patient‐derived tumor slices were fixed overnight in 10% formalin, embedded in paraffin, and sectioned into 4 µm‐thick sections. The sections were analyzed via IF staining using antibodies against F4/80 (ThermoFisher; MA1‐91124) and CD8a (Abcam; ab316778). Cells treated with abemaciclib were fixed with 4% paraformaldehyde (PFA) (Sigma; 441244) for 15 min and then permeabilized with 0.5% Triton X‐100 (Sigma; X100PC). For membrane staining, cells were treated with wheat germ agglutinin (WGA) (ThermoFisher; W11262) before fixation. After blocking with serum, the cells were incubated overnight at 4 °C  in the dark with mouse IF staining antibodies against Ki‐67 (Abcam; ab16667), myeloperoxidase (MPO; ThermoFisher; PA5‐16672), MIF (ThermoFisher; PA5‐27343), CD86 (Abcam; ab220188), CD206 (Cell Signaling Technology; 24595S), or MHC‐I (Novus Biologicals; NBP3‐09017). After washing with PBS‐T, the cells were incubated with secondary antibodies for 1 h at room temperature. Following three PBS‐T washes, nuclei were stained with 0.5 µg mL^−1^ 4′,6‐diamidino‐2‐phenylindole (DAPI; ThermoFisher; 62248) for 10 min at room temperature. Images were captured using a TiE microscope with the NIS Element software and analyzed using a semiautomated in‐house platform based on ImageJ (NIH).

### Western Blotting

Western blotting was performed as described previously^[^ ^73^ ^]^ using antibodies against MIF (ThermoFisher; PA5‐27343), MHC‐I (Novus Biologicals; NBP3‐09017), phospho‐Rb (ThermoFisher; 44‐582G), E2F1 (ThermoFisher; PA5‐116954), NF‐κB (Cell Signaling Technology; 8242), phosphor‐NF‐κB (Cell Signaling Technology; 3033), and β‐actin (Cell Signaling Technology; 4967S). For western blot analysis of MIF in tumor cell supernatant, an equivalent volume of supernatant was harvested and prepared with Laemmli Sample Buffer (Bio‐Rad; 1610747) as described previously.^[^ ^73^ ^]^

### Enzyme‐Linked Immunosorbent Assay (ELISA)

The concentration of MIF in tumor cell‐derived supernatant was measured using the Mouse MIF DuoSet ELISA kit (Bio‐Techne; DY1978), as described previously.^[^ ^74^ ^]^ Briefly, after two days of control or abemaciclib treatment, an equal volume of supernatant was harvested. The concentrations were estimated using a standard curve. If the value exceeded the upper limit of detection (LOD), the samples were re‐evaluated at higher dilutions.

### Flow Cytometry—Isolation of CD8^+^ T Cells and Bone Marrow‐Derived Macrophages

The 1 × 10^6^ cells per tube were stained with appropriate antibodies diluted in FACS buffer (containing 0.2% bovine serum albumin [BSA; Sigma; A2058] and 0.1% sodium azide [NaN_3_; Sigma; S2002] in PBS) for 25–30 min on ice.

### Spleen, Tumor, and Tumor Slices

Single‐cell suspensions were obtained using the tumor tissue enzyme dissociation kit (RWD; DHTE‐5001) with agitation at 37 °C for 30 min after mechanical chopping. The digested mixture was filtered through a 40 µm cell sieve, followed by incubation with RBC lysis buffer for 3 min at room temperature. A total of 1×10^6^ cells per tube was prepared for antibody staining.

### Murine Antibodies

The following antibodies were used: CD45 (Biolegend; 103126), CD3e (Biolegend; 100320), CD8a (Biolegend; 100714), CD4 (Biolegend; 100529), CD28 (Biolegend; 122008), IFNγ (ThermoFisher; 12‐7311‐82), granzyme B (ThermoFisher; 11‐8898‐82), CD11b (Biolegend; 101206), F4/80 (Biolegend; 123116), CD86 (Biolegend; 159204), CD206 (Biolegend; 141720), CD74 (Biolegend; 151006), H‐2Kd/H‐2Dd (ThermoFisher; 11‐5998‐82). Propidium iodide (PI; ThermoFisher; P3566) or DAPI was used to distinguish live/dead cells. For intracellular staining, Fixable Viability Stain 510 (BD Horizon; 564406) was used. Fixable Viability Stain 510 was applied to distinguish live/dead cells in tumor slices. For the proliferation assay, naïve CD8^+^ T cells and bone marrow‐derived macrophages were stained with carboxyfluorescein succinimidyl ester (CFSE; MCE; HY‐D0938) and analyzed via flow cytometry using the FITC channel.

### Cell Cycle Analysis

Tumor cells were fixed with 75% ethanol at 4 °C in the dark for 4 h. The cells were then incubated with a mixture of 400 µL PI (50 µg mL^−1^) and 100 µL RNaseA (100 µg mL^−1^) (Sigma; 10109142001) at 4 °C in the dark for 1 h. The cell cycle distribution was analyzed via flow cytometry using the phycoerythrin channel.

### Tissue Clearing (TC)

As described by Rios et al.,^[^ ^75^ ^]^ tumor slices were incubated overnight in a FUnGI clearing buffer, protected from light at room temperature. Briefly, the FUnGI clearing buffer was prepared by dissolving 100 g of fructose (Sigma; F3510) in 23.3 mL of Tris‐EDTA buffer (100 mm Tris base, pH 8, and 10 mm EDTA [Sigma; 4005]) dissolved in ddH_2_O, and 110 mL of glycerol (Sigma; G5516) at room temperature. Then, 33.1 g urea (Sigma; U5378) was added and stirred until fully homogenized at room temperature. This solution was kept protected from light at 4 °C for use up to 1 month. After TC, the slices were sealed in concave microscope slides for confocal imaging.

### Tumor Slice and Macrophage Coculture Assay

After the lower gel solidified and the tumor slices were laid, 5 × 10^5^ macrophages were mixed with 1 mL of upper gel; 100 µL of the mixed upper gel—containing 5 × 10^4^ macrophages—was then applied to each tumor slice for coculture. After 8 days, tumor slices were carefully extracted from the coated gels and prepared for subsequent experiments.

### 3D immunofluorescence (3D‐IF)—Penetration of Macrophages Into Tumor Slices

5×10^4^ RAW264.7‐GFP or BM‐M1‐CFSE cells were cocultured with tumor slices for 8 days. Thereafter, the tumor slices were dissociated into single‐cell suspensions for FACS analysis or fixed with 95% ethanol on a roller mixer in the dark at 4 °C for 1–2 h. The slices were washed in PBS‐T for 10 min, and then incubated for 30 min in washing buffer (1% Tween‐20, 50 µg mL^−1^ ascorbic acid, 0.05 ng mL^−1^ L‐glutathione in PBS) in the dark at 4 °C. DAPI was used to stain the nuclei overnight. The slices were washed two to three times in washing buffer 2 (0.1% Triton, 0.02% SDS [Sigma; 428015], 0.2% BSA, 50 µg mL^−1^ ascorbic acid [Sigma; 1043003], 0.05 ng mL^−1^ L‐glutathione [Sigma; G4251] in PBS) on a roller mixer in the dark at 4 °C, with the solution replaced every hour.

### 3D‐IF

After fixation with 4% PFA on a roller mixer in the dark at 4 °C for 1 to 2 h, the slices were incubated in washing buffer on a roller mixer in the dark at 4 °C for 1 h. The tissues were further incubated in washing buffer 1 (0.2% Tween‐20 [Sigma; P7949], 0.2% Triton X‐100, 0.02% SDS, 0.2% BSA, 50 µg mL^−1^ ascorbic acid, 0.05 ng mL^−1^ L‐glutathione in PBS) on a roller mixer in the dark at 4 °C for 2 to 3 h. The tumor slices were then incubated overnight in primary antibodies (CD8a [Abcam; ab316778 and ab119857], F4/80 [ThermoFisher; MA1‐91124], CD86 [Abcam; ab220188], CD206 [Cell Signaling Technology; 24595S], HLA‐ABC [Proteintech; 15240‐1‐AP], and granzyme B [Cell Signaling Technology; 17215S]), diluted in washing buffer 2 at appropriate concentrations, on a roller mixer in the dark at 4 °C. After washing with buffer 2 two to three times, the slices were further stained with secondary antibodies and DAPI diluted in washing buffer 2. After two to three washes in washing buffer 2, the slices were ready for TC and imaging using a confocal microscope (A1R, Nikon, Japan).

To investigate the impact of penetrated macrophages on the tumor immune microenvironment, the tumor slices were fixed in 95% ethanol. The tissues were then harvested for 3D‐IF, TC, and confocal imaging. For each region of interest within the confocal images, cell counts and mean minimal distances between cells were quantified using NIS‐Elements AR Analysis 5.30.07. Cell detection was performed by intensity thresholding of the appropriate fluorescent channels, and spatial measurements were calculated based on the segmented objects. Analysis was conducted on three areas per sample with three biological replicates per group.

### Antibodies, Drugs, and Related Reagents

The sources and dilution ratios of the antibodies used for FACS, western blotting, immunofluorescence, and 3D‐immunofluorescence were as follows:

### FACS Antibodies

Pacific Blue anti‐mouse CD45: clone 30‐F11, catalog number 103126, 0.5 µg per 10^6^ cells in 100 µL dilution buffer, Biolegend

PE/Cyanine7 anti‐mouse CD3ε: clone 145‐2C11, catalog number 100320, 0.5 µg 10^−6^ cells in 100 µL dilution buffer, Biolegend

APC/Cyanine7 anti‐mouse CD8a: clone 53–6.7, catalog number 100714, 0.2 µg 10^−6^ cells in 100 µL dilution buffer, Biolegend

Alexa Fluor 488 anti‐mouse CD4: clone RM4‐5, catalog number 100529, 0.5 µg 10^−6^ cells in 100 µL dilution buffer, Biolegend

FITC anti‐mouse CD28: clone E18, catalog number 122008, 0.5 µg 10^−6^ cells in 100 µL dilution buffer, Biolegend

Alexa Fluor 488 anti‐mouse CD74 (CLIP): clone In1/CD74, catalog number 151006, 0.5 µg 10^−6^ cells in 100 µL dilution buffer, Biolegend

FITC‐bound MHC Class I (H‐2Kd/H‐2Dd) monoclonal antibody: clone 34‐1‐2S, catalog number 11‐5998‐82, 0.5 µg 10^−6^ cells in 100 µL dilution buffer, ThermoFisher

APC anti‐mouse F4/80: clone BM8, catalog number 123116, 0.2 µg 10^−6^ cells in 100 µL dilution buffer, Biolegend

FITC anti‐mouse/human CD11b: clone M1/70, catalog number 101206, 0.5 µg 10^−6^ cells in 100 µL dilution buffer, Biolegend

PE anti‐mouse CD86: clone A17199A, catalog number 159204, 0.2 µg 10^−6^ cells in 100 µL dilution buffer, Biolegend

PE/Cyanine7 anti‐mouse CD206 (MMR): clone C068C2, catalog number 141720, 0.2 µg 10^−6^ cells in 100 µL dilution buffer, Biolegend

PE‐bound IFN gamma monoclonal antibody: clone XMG1.2, catalog number 12‐7311‐82, 0.2 µg 10^−6^ cells in 100 µL dilution buffer, ThermoFisher

PE‐bound Granzyme B monoclonal antibody: clone NGZB, catalog number 11‐8898‐82, 0.5 µg 10^−6^ cells in 100 µL dilution buffer, ThermoFisher

PE‐Cyanine7‐bound TNF alpha monoclonal antibody clone MP6‐XT22, catalog number 25‐7321‐82, 0.2 µg 10^−6^ cells in 100 µL dilution buffer, ThermoFisher

### Western Blotting Antibodies

MHC Class I antibody: clone R1‐21.2, catalog number NBP3‐09017, 1:1000 dilution, Novus Biologicals

Phospho‐Rb (Thr821) polyclonal antibody, catalog number 44‐582G, 1:1000 dilution, ThermoFisher

E2F1 polyclonal antibody, catalog number PA5‐116954, 1:1000 dilution, ThermoFisher

MIF polyclonal antibody, catalog number PA5‐27343, 1:1000 dilution, ThermoFisher

NF‐κB p65 antibody, catalog number 8242, 1:1000 dilution, Cell Signaling Technology

Phospho‐NF‐κB p65 (Ser536) antibody, catalog number 3033, 1:1000 dilution, Cell Signaling Technology

β‐Actin antibody, catalog number 4967S, 1:1000 dilution, Cell Signaling Technology

### Immunofluorescence and 3D Immunofluorescence Antibodies

Recombinant CD8a antibody: clone RM1129, catalog number ab316778, 1:100 dilution, Abcam

F4/80 monoclonal antibody: clone CI:A3‐1, catalog number MA1‐91124, 1:100 dilution, ThermoFisher

CD86 Antibody, catalog number ab220188 and ab119857, 1:100 dilution, Abcam

CD206/MRC1 monoclonal antibody: clone E6T5J, catalog number 24595S, 1:400 dilution, Cell Signaling Technology

MIF polyclonal antibody, catalog number PA5‐27343, 1:100 dilution, ThermoFisher

MHC Class I antibody: clone R1‐21.2, catalog number NBP3‐09017, 1:100 dilution, Novus Biologicals

Myeloperoxidase polyclonal antibody, catalog number PA5‐16672, 1:100 dilution, ThermoFisher

HLA class I ABC polyclonal antibody, catalog number 15240‐1‐AP, 1:100 dilution, Proteintech

Granzyme B monoclonal antibody: clone D2H2F, catalog number 17215S, 1:100 dilution, Cell Signaling Technology

Recombinant Anti‐Ki67 antibody: clone SP6, catalog number ab16667, 1:100 dilution, Abcam

### Tumor Cell, Macrophage, and CD8^+^ T Cell Coculture Assay—Macrophage Proliferation and Activation

Three types of coculture models were designed to investigate the proliferation and activation potential of macrophages. For the pretreatment model, 5 × 10^5^ tumor cells were subjected to control or CDK4/6 inhibitor treatment for 2 days in a 6‐well plate. The drug was then removed, and the cells were washed twice with PBS. Thereafter, 5 × 10^5^ macrophages were added to the 6‐well plate for 2 days of coculture. The cells were then used for FACS analysis. For the con‐treatment model, 5 × 10^5^ tumor cells and 5 × 10^5^ million macrophages were cocultured in a 6‐well plate; after 2 days of control or CDK4/6 inhibitor treatment, the cells were harvested for FACS analysis. For the Transwell model, 1 × 10^5^ tumor cells were cultured in a 1 µm pore size insert, and the same quantity of macrophages was cultured in a 24‐well plate (BIOFIL; TCS005006); FACS analysis or IF staining assay was performed after 2 days of drug treatment.

To determine whether the proliferation and activation of macrophages were dependent or independent of tumor cells, macrophage‐alone and supernatant models were also designed. Briefly, macrophages were directly treated with control or CDK4/6 inhibitor for 2 days, followed by FACS analysis. In the supernatant model, tumor cells were treated with control or CDK4/6 inhibitor for 2 days, and the supernatant was harvested. The harvested supernatant was used to treat macrophages directly for 2 days before FACS analysis.

### Proliferation and Activation of CD8^+^ T Cells

Three types of in vitro systems were used—a coculture system, a supernatant model, and a Transwell 2 model—to investigate the proliferation and activation potential of CD8^+^ T cells.

For the coculture system, tumor cells and BM‐M1 or BM‐M2 macrophages (totaling 2 × 10^4^ cells) were cocultured at a relevant ratio in a 24‐well plate with control or CDK4/6 inhibitor treatment for 2 days; thereafter, the drug was completely removed, and 1 × 10^4^ naïve CD8^+^ T cells stained with CFSE or activated CD8^+^ T cells were added to the plate for 4 days of cultivation.

In the supernatant model, after drug removal, the tumor cell and macrophage mixtures were continuously cultivated for 2 days, and the harvested supernatant was used to treat isolated CD8^+^ T cells in a new 24‐well plate for 4 days.

In the Transwell 2 model, 1 × 10^4^ macrophages were cultured in a 24‐well plate, and 1 × 10^4^ tumor cells were added to a 1 µm pore size insert. After 2 days of control or abemaciclib treatment, the drug was completely removed, and 1 × 10^4^ isolated CD8^+^ T cells were directly added to the 24‐well plate for 4 days of coculture.

Finally, these CD8^+^ T cells were harvested for flow cytometry to analyze their proliferation and activation potential.

### Mouse Transcriptome Analysis—Bulk RNA Sequencing

RNA‐seq was performed on six tumor, six spleen, and six tumor cell samples. Quality checks were carried out using MultiQC.^[^ ^76^ ^]^ RNA reads were aligned to the human reference genome, GRCh38, using HISAT2,^[^ ^77^ ^]^ followed by counting of RNA reads with feature Counts,^[^ ^78^ ^]^ a program in the Subread package. Genes with zero read counts across all samples were removed. Differentially expressed genes between control and abemaciclib tumors, control and abemaciclib spleens, and HP5712 and HP5712‐A009 cells were identified using DESeq2.^[^ ^79^ ^]^ The cutoff criteria for DESeq2 were an absolute log2 fold change >1, *p* < 0.05, and a false discovery rate (FDR) <0.05. Multiple hypothesis testing was performed using the Benjamini–Hochberg correction implemented in DESeq2. Gene ontology (GO) analysis was performed using Metascape,^[^ ^80^ ^]^ with results filtered based on a *p* < 0.05 and an FDR <0.05. Gene set enrichment analysis (GSEA) was performed using the GSEA software from the Broad Institute,^[^ ^81^, ^82^ ^]^ with results filtered based on an absolute normalized enrichment score (NES) >1, *p* < 0.05, and an FDR <0.05.

### Single Cell RNA‐Seq

After 10 days of in vivo control or abemaciclib treatment, three tumors from the control or abemaciclib group were combined to prepare cell suspensions. The cell suspensions were loaded onto a 10X Genomics GemCode Single‐cell instrument, which generates single‐cell Gel Bead‐In‐Emulsion (GEMs), followed by cell counting and quality control. Libraries were generated and sequenced from the cDNAs using Chromium Next GEM Single Cell 3′ Reagent Kits v3.1. Upon dissolution of the Gel Bead in a GEM, primers containing i) an Illumina R1 sequence (read 1 sequencing primer), ii) a 16‐nt 10x barcode, iii) a 10‐nt Unique Molecular Identifier (UMI), and iv) a poly‐dT primer sequence were released and mixed with cell lysate and Master Mix. Barcoded, full‐length cDNAs were then reverse‐transcribed from polyadenylated mRNA. After GEM‐RT cleanup using Silane magnetic beads and cDNA amplification via PCR, sufficient mass was obtained to proceed with library construction. Briefly, R1 (read 1 primer sequence) was added to the molecules during GEM incubation. P5, P7, a sample index, and R2 (read 2 primer sequence) were added during library construction via end repair, A‐tailing, adaptor ligation, and PCR. The final libraries contained the P5 and P7 primers used in Illumina bridge amplification. The Single Cell 3′ Protocol produced Illumina‐ready sequencing libraries. A Single Cell 3′ Library comprised standard Illumina paired‐end constructs that started and ended with P5 and P7, respectively. The Single Cell 3′ 16 bp 10x Barcode and 10 bp UMI were encoded in Read 1, whereas Read 2 was used to sequence the cDNA fragment. Sample index sequences were incorporated as the i7 index read. Read 1 and Read 2 were the standard Illumina sequencing primer sites used in paired‐end sequencing.

### Bioinformatic Analysis

The raw BCL files were converted using the 10X Genomics Cell Ranger software (version 3.1.0) into FASTQ files for alignment and gene expression quantification. The cell‐by‐gene matrices for each sample were then individually imported into Scanpy version 1.9.1 for cell type annotation and downstream analyses. In particular, cells with an unusually high number of UMIs (≥8,000) or mitochondrial gene percentage (≥10%) were filtered out. Cells with fewer than 500 or more than 4 000 genes detected were also excluded. Doublet GEMs were removed using the tool Scrublet (v0.2.3). The gene expression measurements for each cell were normalized by the total expression according to the following formula:

(1) $$\mathrm{Gene}\;\mathrm{expression}\;\mathrm{level}=\log\left(1+\mathrm{UM}I_{A}/\mathrm{UM}I_{\mathrm{Total}}\times 10,000\right)$$

To minimize the effects of batch variation and behavioral conditions on clustering, Harmony, an algorithm that projects cells into a shared embedding where cells are grouped by cell type rather than dataset‐specific conditions, was used to aggregate all samples. The Harmony algorithm inputs a principal component (PC) analysis embedding of cells, along with their batch assignments, and returns a batch‐corrected embedding. The integrated expression matrix was then scaled and subjected to principal component analysis for dimensionality reduction. The PCs with the highest contribution were identified using a scree test. Scanpy implements a graph‐based clustering approach. Distances between cells were calculated based on previously identified PCs. Cells were then clustered using the Louvain method to maximize modularity. For visualization of clusters, uniform manifold approximation and projection (UMAP) was generated using the same PCs. For cell annotation, the log‐normalized matrices were loaded into SingleR against the murine ImmGen reference, which is based on correlating gene expression of reference cell types with single‐cell expression. The Python package “CellPhoneDB” was used to analyze cell–cell communications between two cell types based on the expression of a receptor by one cell type and a ligand by another. To identify the most relevant interactions between cell types, cell‐type‐specific interactions between ligands and receptors were identified.

### TCGA Analysis

The public normalized gene expression data based on fragments per kilobase of exon model per million reads mapped (FPKM) for breast cancers were obtained from the TCGA data portal (http://gdac.broadinstitute.org/). Patients were divided into two groups according to their *RB1* and *E2F1* expression levels—those with gene expression above the median value were classified as the high G_1_/S transition activity cohort, and those with gene expression below the median value were classified as the low G_1_/S transition activity cohort. The Pearson correlation coefficient between *CD8A* and *CD86* genes was calculated to assess their linear relationship in the low G_1_/S transition activity cohort. Survival analysis was performed using the R package “survival” in both the high and low G_1_/S transition activity cohorts. In both cohorts, patients were further classified as *CD74*‐high or *CD74*‐low expressers based on whether their *CD74* expression was above or below the median value. Patients with multiple cancer types were divided into *CD8A* ^−^/*IFNG* ^−^ and *CD8A* ^+^/*IFNG* ^+^ groups based on their expression levels of *CD8A* and *IFNG*, similar to the classification of *RB1* and *E2F1*. The Kaplan–Meier survival curve was modeled using the survfit function, and *p*‐values were reported.

### Protein Mass Spectrometry

Protein mass spectrometry was performed using a data‐independent acquisition (DIA) model. Briefly, 10 mL of supernatant from 1 × 10^6^ HP5712 tumor cells cocultured with 1 × 10^6^ FVB mouse BM‐M1 macrophages after 2 days of control or abemaciclib treatment (*n* = 3 samples per group) was harvested to extract proteins, which were quality‐checked using the SDS‐PAGE method. The same quantity of protein from each sample was used for enzymolysis via filter aided sample preparation. LC‐Orbitrap MS was employed to obtain high‐quality direct DIA data from each sample. The directDIA data were preprocessed using the Spectronaut software to determine protein expression levels and to select differentially expressed proteins. R packages “clusterProfiler,” “enrichplot,” and “GOplot” were applied for analysis.

### sgRNA Experiments

The sequences encoding the small‐guide RNA (sgRNA) targeting *Mif*, *Hif‐1α*, or *Cd74* were cloned into the lentiCRISPRv2 construct to create sgMIF or sgCD74 plasmids, respectively. Three different encoding oligonucleotides were used for sg*Mif* (oligo 1, 5′‐GCAAACCTGTGCGGGCTTGC‐3′, 3′‐GCAAGCCCGCACAGGTTTGC‐5′; oligo 2, 5′‐GGCCACCGGCAAGCCCGCAC‐3′, 3′‐GTGCGGGCTTGCCGGTGGCC‐5′; oligo 3, 5′‐CCAGTACATCGCAGTGCACG‐3′, 3′‐CGTGCACTGCGATGTACTGG‐5′), sg*Hif‐1α* (oligo 1, 5′‐CAAGATGTGAGCTCACATTG‐3′, 3′‐CAATGTGAGCTCACATCTTG‐5′; oligo 2, 5′‐TGTTTGCAGTTTGAACTAAC‐3′, 3′‐GTTAGTTCAAACTGCAAACA‐5′; oligo 3, 5′‐GTGATGGTGCTAACAGATGA‐3′, 3′‐TCATCTGTTAGCACCATCAC‐5′), and sg*Cd74* (oligo 1, 5′‐GCTGATGCGTCCAATGTCCA‐3′, 3′‐TGGACATTGGACGCATCAGC‐5′; oligo 2, 5′‐ATTTCGGAAGCTTCATGCGA‐3′, 3′‐TCGCATGAAGCTTCCGAAAT‐5′; oligo 3, 5′‐GAGGTCGCGTTGGTCATCCA‐3′, 3′‐TGGATGACCAACGCGACCTC‐5′). To obtain stable *Mif* or *Hif‐1α* knockout in tumor cells, HEK293T cells were used to transfect sg*Mif* or sg*Hif‐1α* plasmids into HP5712 and 4T1 cells in a 6‐well plate, and puromycin was used to select the stable colonies. The *Mif* or *Hif‐1α* knockout efficiency was validated via western blotting. Lipofectamine 3000 transfection reagent was used to transfect sg*Cd74* into mouse BM‐M1 macrophages in a 6‐well plate for 3 days. The total CD74 expression (including that in membrane and nuclear fractions) in macrophages was investigated via FACS analysis.

### Statistical Analysis

The GraphPad Prism 9 software was used to perform the statistical analysis. The figure legends describe the statistical analysis for each experiment. Two‐tailed unpaired Student's *t*‐test was used to compare datasets between two groups with normal distributions and equal standard deviations, whereas unpaired *t*‐test with Welch's correction was used for samples with significantly different standard deviations. One‐way ANOVA with Tukey's multiple comparisons test was used to compare datasets among multiple groups (≥3) with equal standard deviations, and one‐way ANOVA with Dunnett's multiple comparison test was applied for samples with unequal standard deviations. For two groups with different experimental designs (≥2), two‐way ANOVA with multiple comparisons test was performed. Survival was measured using the Kaplan–Meier method. Pearson's correlation was used to calculate the correlation between two genes. A significant difference between groups was considered at a *p* < 0.05. All data shown represent three or more independent experiments.

## Conflict of Interest

The authors declare no conflict of interest.

## Author Contributions

All authors revised and approved the manuscript. L.H. performed the experiments, analyzed the experiment data, and prepared the figures and manuscript. Y. and J.Z. performed the bioinformatic analysis. L.‐l.L., W.W., Y.Y., and Y.Q. were involved in the human sample collection and transportation. D.T., W.W., X.W., J.h.L., Y.F., X.C., D.M., Q.Z., and T.L. contributed to the methodology. Y.C. contributed to the ethics approval for the use of human samples. C.D. and P.K.T. designed the project, supervised all data, and cowrote the manuscript.

## Supporting information



Supporting Information

## Data Availability

All data supporting the results presented in the paper are present in the paper and/or the Supplementary Materials. The original datasets are also available from the corresponding author upon request. The RNA‐seq data have been deposited in the Sequence Read Archive (SRA) database with the following access number: PRJCA032080 https://www.ncbi.nlm.nih.gov/bioproject/PRJCA032080/. The DIA‐MS proteomics data have been deposited in the PRoteomics IDEntifications Database (PRIDE) with the following access number: PXD057791 (DIA raw data) http://www.ebi.ac.uk/pride/archive/projects/PXD057791. Public data from The Cancer Genomic Atlas (TCGA‐BRCA.sampleMap/HiSeqV2) database were used. Source data are provided with this paper.

## References

[1] C. Swanton , E. Bernard , C. Abbosh , F. André , J. Auwerx , A. Balmain , D. Bar‐Sagi , R. Bernards , S. Bullman , J. DeGregori , C. Elliott , A. Erez , G. Evan , M. A. Febbraio , A. Hidalgo , M. Jamal‐Hanjani , J. A. Joyce , M. Kaiser , K. Lamia , J. W. Locasale , S. Loi , I. Malanchi , M. Merad , K. Musgrave , K. J. Patel , S. Quezada , J. A. Wargo , A. Weeraratna , E. White , F. Winkler , Cell 2024, 187, 1589. 38552609 10.1016/j.cell.2024.02.009 PMC12077170

[2] D. Hanahan , Cancer discovery 2022, 12, 31. 35022204 10.1158/2159-8290.CD-21-1059

[3] J. L. Teh , A. E. Aplin , Clin. Cancer Res. 2019, 25, 921. 30287548 10.1158/1078-0432.CCR-18-1967 PMC6359975

[4] C. J. Sherr , J. M. Roberts , Genes Dev. 1999, 13, 1501. 10385618 10.1101/gad.13.12.1501

[5] A. Y. Lai , J. A. Sorrentino , K. H. Dragnev , J. M. Weiss , T. K. Owonikoko , Immunother Cancer 2020, 8, 000847. 10.1136/jitc-2020-000847 PMC7534680 33004541

[6] S. Goel , M. J. DeCristo , A. C. Watt , H. BrinJones , J. Sceneay , B. B. Li , N. Khan , J. M. Ubellacker , S. Xie , O. Metzger‐Filho , J. Hoog , M. J. Ellis , C. X. Ma , S. Ramm , I. E. Krop , E. P. Winer , T. M. Roberts , H.‐J. Kim , S. S. McAllister , J. J. Zhao , Nature 2017, 548, 471. 28813415 10.1038/nature23465 PMC5570667

[7] X. Gao , G. W. Leone , H. Wang , Adv. Cancer Res. 2020, 148, 147. 32723562 10.1016/bs.acr.2020.02.002

[8] J. Deng , E. S. Wang , R. W. Jenkins , S. Li , R. Dries , K. Yates , S. Chhabra , W. Huang , H. Liu , A. R. Aref , E. Ivanova , C. P. Paweletz , M. Bowden , C. W. Zhou , G. S. Herter‐Sprie , J. A. Sorrentino , J. E. Bisi , P. H. Lizotte , A. A. Merlino , M. M. Quinn , L. E. Bufe , A. Yang , Y. Zhang , H. Zhang , P. Gao , T. Chen , M. E. Cavanaugh , A. J. Rode , E. Haines , Cancer discovery 2018, 8, 216.29101163

[9] D. A. Schaer , R. P. Beckmann , J. A. Dempsey , L. Huber , A. Forest , N. Amaladas , Y. Li , Y. C. Wang , E. R. Rasmussen , D. Chin , A. Capen , C. Carpenito , K. A. Staschke , L. A. Chung , L. M. Litchfield , F. F. Merzoug , X. Gong , P. W. Iversen , S. Buchanan , A. de Dios , R. D. Novosiadly , M. Kalos , Cell Rep. 2018, 22, 2978. 29539425 10.1016/j.celrep.2018.02.053

[10] P. A. Ott , Y.‐J. Bang , S. A. Piha‐Paul , A. R. A Razak , J. Bennouna , J.‐C. Soria , H. S. Rugo , R. B. Cohen , B. H. O'Neil , J. M. Mehnert , J. Lopez , T. Doi , E. M. J. van Brummelen , R. Cristescu , P. Yang , K. Emancipator , K. Stein , M. Ayers , A. K. Joe , J. K. Lunceford , J. Clin. Oncol. 2019, 37, 318. 30557521 10.1200/JCO.2018.78.2276

[11] K. Muro , H. C. Chung , V. Shankaran , R. Geva , D. Catenacci , S. Gupta , J. P. Eder , T. Golan , D. T. Le , B. Burtness , A. J. McRee , C.‐C. Lin , K. Pathiraja , J. Lunceford , K. Emancipator , J. Juco , M. Koshiji , Y.‐J. Bang , Lancet Oncol. 2016, 17, 717. 27157491 10.1016/S1470-2045(16)00175-3

[12] E. B. Garon , N. A. Rizvi , R. Hui , N. Leighl , A. S. Balmanoukian , J. P. Eder , A. Patnaik , C. Aggarwal , M. Gubens , L. Horn , E. Carcereny , M.‐J. Ahn , E. Felip , J.‐S. Lee , M. D. Hellmann , O. Hamid , J. W. Goldman , J.‐C. Soria , M. Dolled‐Filhart , R. Z. Rutledge , J. Zhang , J. K. Lunceford , R. Rangwala , G. M. Lubiniecki , C. Roach , K. Emancipator , L. Gandhi , N. Engl. J. Med. 2015, 372, 2018.25891174

[13] A. B. El‐Khoueiry , B. Sangro , T. Yau , T. S. Crocenzi , M. Kudo , C. Hsu , T.‐Y. Kim , Su‐P Choo , J. Trojan , T. H. Welling , T. Meyer , Y.‐K. Kang , W. Yeo , A. Chopra , J. Anderson , C. dela Cruz , L. Lang , J. Neely , H. Tang , H. B. Dastani , I. Melero , Lancet 2017, 389, 2492. 28434648 10.1016/S0140-6736(17)31046-2 PMC7539326

[14] J. Larkin , V. Chiarion‐Sileni , R. Gonzalez , J. J. Grob , C. L Cowey , C. D. Lao , D. Schadendorf , R. Dummer , M. Smylie , P. Rutkowski , P. F. Ferrucci , A. Hill , J. Wagstaff , M. S. Carlino , J. B. Haanen , M. Maio , I. Marquez‐Rodas , G. A. McArthur , P. A. Ascierto , G. V. Long , M. K. Callahan , M. A. Postow , K. Grossmann , M. Sznol , B. Dreno , L. Bastholt , A. Yang , L. M. Rollin , C. Horak , F. S Hodi , et al., N. Engl. J. Med. 2015, 373, 23. 26027431 10.1056/NEJMoa1504030 PMC5698905

[15] C. Robert , J. Schachter , G. V. Long , A. Arance , J. J. Grob , L. Mortier , A. Daud , M. S. Carlino , C. McNeil , M. Lotem , J. Larkin , P. Lorigan , B. Neyns , C. U. Blank , O. Hamid , C. Mateus , R. Shapira‐Frommer , M. Kosh , H. Zhou , N. Ibrahim , S. Ebbinghaus , A. Ribas , N. Engl. J. Med 2015, 372, 2521.25891173

[16] M. Reck , D. Rodríguez‐Abreu , A. G. Robinson , R. Hui , T. Csoszi , A. Fülöp , M. Gottfried , N. Peled , A. Tafreshi , S. Cuffe , M. O'Brien , S. Rao , K. Hotta , M. A. Leiby , G. M. Lubiniecki , Y. Shentu , R. Rangwala , J. R. Brahmer , N. Engl. J. Med 2016, 375, 1823.27718847

[17] J. Yu , J. Yan , Q. Guo , Z. Chi , B. Tang , B. Zheng , J. Yu , T. Yin , Z. Cheng , X. Wu , H. Yu , J. Dai , X. Sheng , L. Si , C. Cui , X. Bai , L. Mao , B. Lian , X. Wang , X. Yan , S. Li , Li Zhou , K. T. Flaherty , J. Guo , Y. Kong , Clin. Cancer Res. 2019, 25, 6511. 31375512 10.1158/1078-0432.CCR-19-0475

[18] J. L. F. Teh , D. A. Erkes , P. F. Cheng , Cancer Immunol. Res. 2020, 8, 1114. 32661093 10.1158/2326-6066.CIR-19-0743 PMC7484433

[19] Q.‐F. Zhang , J. Li , K. Jiang , R. Wang , J.‐L. Ge , H. Yang , S.‐J. Liu , L.‐T. Jia , L. Wang , Bi‐L Chen , Theranostics 2020, 10, 10619. 32929370 10.7150/thno.44871 PMC7482823

[20] Q. Long , A.‐H Ma , H. Zhang , Z. Cao , R. Xia , T.‐Y. Lin , G. P. Sonpavde , R. de Vere White , J. Guo , C.‐X. Pan , Cancer Immunol., Immunother. : CII 2020, 69, 2305. 32506263 10.1007/s00262-020-02609-5 PMC7572640

[21] A. R. Tan , G. S. Wright , Clin. Cancer Res. 2022, 28, 629. 34887261 10.1158/1078-0432.CCR-21-2272 PMC9377748

[22] D. Daniel , V. Kuchava , I. Bondarenko , O. Ivashchuk , S. Reddy , J. Jaal , I. Kudaba , L. Hart , A. Matitashvili , Y. Pritchett , S. R. Morris , J. A. Sorrentino , J. M. Antal , J. Goldschmidt , Int. J. Cancer 2020, 148, 2557. 10.1002/ijc.33453 PMC8048941 33348420

[23] E. J. Lelliott , G. A. McArthur , J. Oliaro , K. E. Sheppard , Front. Immunol. 2021, 12, 661737. 34025662 10.3389/fimmu.2021.661737 PMC8137893

[24] J. Zhang , X. Bu , H. Wang , Y. Zhu , Y. Geng , N. T. Nihira , Y. Tan , Y. Ci , F. Wu , X. Dai , J. Guo , Yu‐H Huang , C. Fan , S. Ren , Y. Sun , G. J. Freeman , P. Sicinski , W. Wei , Nature 2018, 553, 91. 29160310 10.1038/nature25015 PMC5754234

[25] C. Egelston , W. Guo , S. Yost , J. S. Lee , D. Rose , C. Avalos , J. Ye , P. Frankel , D. Schmolze , J. Waisman , P. Lee , Y. Yuan , J. Immunother. Cancer 2021, 9, 002084. 10.1136/jitc-2020-002084 PMC7993344 33757987

[26] X. Bai , Z. Q. Guo , Nat. Commun. 2023, 14, 1247. 36871040 10.1038/s41467-023-36892-4 PMC9985635

[27] S. P. Nobs , M. Kopf , Trends Immunol. 2021, 42, 495. 33972166 10.1016/j.it.2021.04.007

[28] D. J. Kloosterman , L. Akkari , Cell 2023, 186, 1627. 36924769 10.1016/j.cell.2023.02.020

[29] C. Hernandez , P. Huebener , R. F. Schwabe , Oncogene 2016, 35, 5931. 27086930 10.1038/onc.2016.104 PMC5119456

[30] D. Laoui , E. Van Overmeire , P. De Baetselier , J. A. Van Ginderachter , G. Raes , Front. Immunol. 2014, 5, 489. 25339957 10.3389/fimmu.2014.00489 PMC4188035

[31] F. Xing , Y.‐C Liu , S. Huang , X. Lyu , S. M. Su , U. I. Chan , P.‐C. Wu , Y. Yan , N. Ai , J. Li , M. Zhao , B. K. Rajendran , J. Liu , F. Shao , H. Sun , T. K. Choi , W. Zhu , G. Luo , S. Liu , D. L. Xu , K. L. Chan , Qi Zhao , K. Miao , K. Q. Luo , W. Ge , X. Xu , G. Wang , T.‐M. Liu , C.‐X. Deng , Theranostics 2021, 11, 9415. 34646378 10.7150/thno.59533 PMC8490519

[32] L. He , C. Deng , Int. J. Biol. Sci. 2022, 18, 5885. 36263166 10.7150/ijbs.78997 PMC9576528

[33] J. E. Smith‐Garvin , G. A. Koretzky , M. S. Jordan , Annu. Rev. Immunol. 2009, 27, 591. 19132916 10.1146/annurev.immunol.021908.132706 PMC2740335

[34] Y. Li , Y. He , K. Miao , Y. Zheng , C. Deng , T. M. Liu , Theranostics 2020, 10, 2897. 32194843 10.7150/thno.40495 PMC7053213

[35] J. M. Weiss , L. A. Ridnour , T. Back , S. P Hussain , P. He , A. E. Maciag , L. K. Keefer , W. J. Murphy , C .C. Harris , D. A. Wink , R .H. Wiltrout , J. Exp. Med. 2010, 207, 2455. 20921282 10.1084/jem.20100670 PMC2964582

[36] E. Karimi , M. W. Yu , Nature 2023, 614, 555. 36725935 10.1038/s41586-022-05680-3 PMC9931580

[37] I. Kang , R. Bucala , Nat. Rev. Rheumatol. 2019, 15, 427. 31197253 10.1038/s41584-019-0238-2

[38] M. J. Kim , W. S. Kim , D. O. Kim , J.‐E. Byun , H. Huy , S. Y. Lee , H. Y. Song , Y.‐J. Park , T.‐D. Kim , S. R. Yoon , E.‐J. Choi , H. Ha , H. Jung , I. Choi , Cellular Signall. 2017, 34, 110. 10.1016/j.cellsig.2017.03.007 28323005

[39] J. Li , J. Zhang , F. Xie , J. Peng , X. Wu , Int. J. Mol. Med. 2018, 41, 1062. 29207023 10.3892/ijmm.2017.3277

[40] Z‐Bo Shang , J. Wang , S.‐G. Kuai , Y.‐Y. Zhang , Q.‐F. Ou , H. Pei , Li‐H Huang , Ann. Lab. Med. 2018, 38, 9. 29071813 10.3343/alm.2018.38.1.9 PMC5700157

[41] T. Roger , A. Schneider , M. Weier , F. C. G. J. Sweep , D. Le Roy , J. Bernhagen , T. Calandra , E. Giannoni , Proc. Natl. Acad. Sci. USA 2016, 113, E997. 26858459 10.1073/pnas.1514018113 PMC4776487

[42] H. S. Rugo , P. Kabos , J. T. Beck , G. Jerusalem , H. Wildiers , NPJ Breast Cancer 2022, 8, 118. 36335120 10.1038/s41523-022-00482-2 PMC9637121

[43] L. He , P. K. Tam , C. X. Deng , Int. J. Biol. Sci. 2025, 21, 4098. 40607254 10.7150/ijbs.115932 PMC12210125

[44] E. J. Lelliott , I. Y. Kong , M. Zethoven , Cancer Discovery 2021, 11, 2582.33990344

[45] T. Calandra , T. Roger , Nat. Rev. Immunol. 2003, 3, 791. 14502271 10.1038/nri1200 PMC7097468

[46] K. Sumaiya , D. Langford , K. Natarajaseenivasan , S. Shanmughapriya , Pharmacol. Ther. 2022, 233, 108024. 34673115 10.1016/j.pharmthera.2021.108024

[47] R. A. Mitchell , R. Bucala , Semin. Cancer Biol. 2000, 10, 359. 11100884 10.1006/scbi.2000.0328

[48] J. Fan , Y. Chen , H. M. Chan , P. K. Tam , Y. Ren , Proc. Natl. Acad. Sci. USA 2005, 102, 17751. 16314559 10.1073/pnas.0509175102 PMC1308934

[49] B. Jäger , D. Klatt , L. Plappert , H. Golpon , S. Lienenklaus , P. D. Barbosa , A. Schambach , A. Prasse , Cellular Signall. 2020, 73, 109672. 10.1016/j.cellsig.2020.109672 32428553

[50] J. D. Hudson , M. A. Shoaibi , R. Maestro , A. Carnero , G. J. Hannon , D. H. Beach , J. Exp. Med. 1999, 190, 1375. 10562313 10.1084/jem.190.10.1375 PMC2195698

[51] M. Chen , H. Liu , FASEB Journal 2024, 38, 23696.

[52] K. N. Balogh , D. J. Templeton , J. V. Cross , PLoS One 2018, 13, 0197702. 10.1371/journal.pone.0197702 PMC5986154 29864117

[53] F. H. G. Tessaro , E. Y. Ko , M. De Simone , R. Piras , M. T. Broz , H. S. Goodridge , B. Balzer , S. L. Shiao , J. Guarnerio , Cell Rep. 2022, 39, 110977. 35732118 10.1016/j.celrep.2022.110977 PMC9249098

[54] B. A. Castro , P. Flanigan , A. Jahangiri , D. Hoffman , W. Chen , R. Kuang , M. De Lay , G. Yagnik , J. R. Wagner , S. Mascharak , M. Sidorov , S. Shrivastav , G. Kohanbash , H. Okada , M. K. Aghi , Oncogene 2017, 36, 3749. 28218903 10.1038/onc.2017.1 PMC5491354

[55] P. T. Ferreira , A. C. Oliveira‐Scussel , R. A. Sousa , B. Q. Gomes , J. E. Félix , R. J. Silva , I. B. Millian , T. S. Assunção , S. C. Teixeira , M. D. L. M Gomes , M. V. Silva , B. F. Barbosa , V. Rodrigues Junior , J. R. Mineo , C. J. Oliveira , E. A. Ferro , A. O. Gomes , Immunobiology 2023, 228, 152357. 36857907 10.1016/j.imbio.2023.152357

[56] L. Wang , H. Wang , L. Yu , H. Jiang , L. Xia , bioRxiv 2023, 2023, 10.

[57] T. Calandra , J. Bernhagen , R. A. Mitchell , R. Bucala , J. Exp. Med. 1994, 179, 1895. 8195715 10.1084/jem.179.6.1895 PMC2191507

[58] K. Yaddanapudi , K. Putty , B. E. Rendon , G. J. Lamont , J. D. Faughn , A. Satoskar , A. Lasnik , J. W. Eaton , R. A. Mitchell , J. Immunol. (Baltimore, Md : 1950) 2013, 190, 2984. 10.4049/jimmunol.1201650 PMC3593945 23390297

[59] J. Huang , L. Zheng , Z. Sun , J. Li , Int. J. Mol. Med. 2022, 50, 128. 36043521 10.3892/ijmm.2022.5184 PMC9448295

[60] M. C. Papadimitriou , A. Pazaiti , K. Iliakopoulos , M. Markouli , V. Michalaki , C. A. Papadimitriou , BBA‐Mol. Cell Res. 2022, 1869, 119346. 10.1016/j.bbamcr.2022.119346 36030016

[61] X.‐Q. Xu , X.‐H. Pan , T.‐T. Wang , J. Wang , Bo Yang , Q.‐J. He , L. Ding , Acta Pharmacol. Sin. 2021, 42, 171. 32504067 10.1038/s41401-020-0416-4 PMC8027849

[62] N. M. Kettner , T. N. Bui , J. Navarro‐Yepes , S. Ghotbaldini , B. Quintela , C. K. Luo , N. Lam , X. Rao , A. S. Raghavendra , Y. Wang , N. Azizian , T. Kris Eckols , M. M. Kasembeli , K. Evans , M. Yi , H. Wingate , J. Wang , A. A. Sahin , F. Meric‐Bernstam , K. K. Hunt , S. Damodaran , D. J. Tweardy , D. Tripathy , K. Keyomarsi , NPJ Prec. Oncol. 2025, 9, 260. 10.1038/s41698-025-01041-1 PMC12297654 40715551

[63] S. Hume , G. L. Dianov , K. Ramadan , Nucleic Acids Res. 2020, 48, 12483. 33166394 10.1093/nar/gkaa1002 PMC7736809

[64] M. E. Hubbi , D. M. Gilkes , H. Hu , A. I. Kshitiz , G. L. Semenza , Proc. Natl. Acad. Sci. USA 2014, 111, E3325. 25071185 10.1073/pnas.1412840111 PMC4136593

[65] T. Wang , H. Liu , G. Lian , S. Y. Zhang , X. Wang , Mediators of Inflammation 2017, 2017, 9029327. 29386753 10.1155/2017/9029327 PMC5745720

[66] A. M. Abdul‐Aziz , M. S. Shafat , Yu Sun , C. R. Marlein , R. E. Piddock , S. D. Robinson , D. R. Edwards , Z. Zhou , A. Collins , K. M. Bowles , S. A. Rushworth , Oncogene 2018, 37, 2676. 29487418 10.1038/s41388-018-0151-1

[67] J. K. Tiwari , S. Negi , M. Kashyap , S. Nizamuddin , A. Singh , A. Khattri , Front. Oncol. 2021, 11, 793881. 35096592 10.3389/fonc.2021.793881 PMC8790577

[68] V. Quaranta , M. C. Schmid , Cells 2019, 8, 747.31331034

[69] B. Ruffell , D. Chang‐Strachan , V. Chan , A. Rosenbusch , C. M. T. Ho , N. Pryer , D. Daniel , E. S Hwang , H. S. Rugo , L. M. Coussens , Cancer Cell 2014, 26, 623. 25446896 10.1016/j.ccell.2014.09.006 PMC4254570

[70] M. Klichinsky , M. Ruella , Nat. Biotechnol. 2020, 38, 947. 32361713 10.1038/s41587-020-0462-y PMC7883632

[71] C. Sloas , S. Gill , M. Klichinsky , Front. Immunol. 2021, 12, 783305. 34899748 10.3389/fimmu.2021.783305 PMC8652144

[72] S. Goel , Qi Wang , A. C. Watt , S. M. Tolaney , D. A. Dillon , W. Li , S. Ramm , A. C. Palmer , H. Yuzugullu , V. Varadan , D. Tuck , L. N. Harris , K.‐K. Wong , X. S Liu , P. Sicinski , E. P. Winer , I. E. Krop , J. J. Zhao , Cancer Cell 2016, 29, 255. 26977878 10.1016/j.ccell.2016.02.006 PMC4794996

[73] J. Li , X. Shu , J. Xu , S. M. Su , U. I. Chan , L. Mo , J. Liu , X. Zhang , R. Adhav , Q. Chen , Y. Wang , T. An , Xu Zhang , X. Lyu , X. Li , J. H. Lei , K. Miao , H. Sun , F. Xing , A. Zhang , C. Deng , X. Xu , Nat. Commun. 2022, 13, 1481. 35304461 10.1038/s41467-022-29151-5 PMC8933470

[74] M. Mekhaeil , M. J. Conroy , K. K. Dev , Neurotherapeutics 2023, 20, 1347. 37525026 10.1007/s13311-023-01409-w PMC10480139

[75] A. C. Rios , B. D. Capaldo , F. Vaillant , B. Pal , R. van Ineveld , C. A. Dawson , Y. Chen , E. Nolan , N. Y. Fu , F. C. Jackling , S. Devi , D. Clouston , L. Whitehead , G. K. Smyth , S. N. Mueller , G. J. Lindeman , J. E. Visvader , Cancer Cell 2019, 35, 618. 30930118 10.1016/j.ccell.2019.02.010

[76] P. Ewels , M. Magnusson , S. Lundin , M. Käller , Bioinformatics (Oxford, England) 2016, 32, 3047. 27312411 10.1093/bioinformatics/btw354 PMC5039924

[77] D. Kim , B. Langmead , S. L. Salzberg , Nat. Methods 2015, 12, 357. 25751142 10.1038/nmeth.3317 PMC4655817

[78] Y. Liao , G. K. Smyth , W. Shi , Bioinformatics (Oxford, England) 2014, 30, 923. 24227677 10.1093/bioinformatics/btt656

[79] M. I. Love , W. Huber , S. Anders , Genome Biol. 2014, 15, 550. 25516281 10.1186/s13059-014-0550-8 PMC4302049

[80] Y. Zhou , B. Zhou , L. Pache , M. Chang , Nat. Commun. 2019, 10, 1523. 30944313 10.1038/s41467-019-09234-6 PMC6447622

[81] A. Subramanian , P. Tamayo , V. K. Mootha , S. Mukherjee , B. L. Ebert , M. A. Gillette , A. Paulovich , S. L. Pomeroy , T. R. Golub , E. S. Lander , J. P. Mesirov , Proc. Natl. Acad. Sci. USA 2005, 102, 15545. 16199517 10.1073/pnas.0506580102 PMC1239896

[82] V. K. Mootha , C. M. Lindgren , K.‐F. Eriksson , A. Subramanian , S. Sihag , J. Lehar , P. Puigserver , E. Carlsson , M. Ridderstråle , E. Laurila , N. Houstis , M. J. Daly , N. Patterson , J. P. Mesirov , T. R. Golub , P. Tamayo , B. Spiegelman , E. S. Lander , J. N. Hirschhorn , D. Altshuler , L. C. Groop , Nat. Genet. 2003, 34, 267. 12808457 10.1038/ng1180

