# Supplementary material for: CDK4/6 Inhibition Induces CD8+ T Cell Antitumor Immunity via MIF‐Induced Functional Orchestration of Tumor‐Associated Macrophages
Source: Adv Sci (Weinh). 2025 Oct 13;13(35):e11330. doi: 10.1002/advs.202511330 (PMC13292170; doi:10.1002/advs.202511330)

CDK4/6 Inhibition Induces $CD8^+$ T Cell Antitumor Immunity via MIF-Induced Functional Orchestration of Tumor-Associated Macrophages

Lin He[1,2,3,4,5], Yuzhong Peng[1,2,3], Lat-lun Leong[6], Jingbo Zhou[7], Dongyang Tang[1,2,3], Weilu Wang[4,5], Xiaoran Wu[1,2,3], Josh haipeng Lei[1,2,3], Yongqin Ye[4,5], Yangyang Feng[1,2,3], Yunfeng Qiao[1,2,3], Xiangpeng Chu[1,2,3], Di Mu[1,2,3], Qi Zhao[1,2,3], Tzuming Liu[1,2,3], Yan Chen[4,5], Paul Kwonghang Tam[4,5,*], Chu-Xia Deng[1,2,3,8,*]

1. Cancer Center, Faculty of Health Sciences, University of Macau, Macau SAR, 999078, China

2. Center for Precision Medicine Research and Training, Faculty of Health Sciences, University of Macau, Macau SAR, 999078, China

3. MOE Frontier Science Center for Precision Oncology, University of Macau, Macau SAR, 999078, China

4. Medical Sciences Division, Macau University of Science and Technology, Macau SAR, 999078, China

5. Precision Regenerative Medicine Research Centre, Macau University of Science and Technology, Macau SAR, 999078, China

6. University Hospital, Macau University of Science and Technology, Macau SAR, 999078, China

7. Guangdong Key Laboratory of New Technology in Rice Breeding, Rice Research Institute, Guangdong Academy of Agricultural Sciences, Guangzhou, 510640, China

8. Lead contact

*Correspondence: pkhtam@must.edu.mo, and cxdeng@um.edu.mo.

Supplementary Figures

Figure S1. CDK4/6 inhibition creates an immunostimulatory status in the tumor microenvironment but not in the circulatory system.

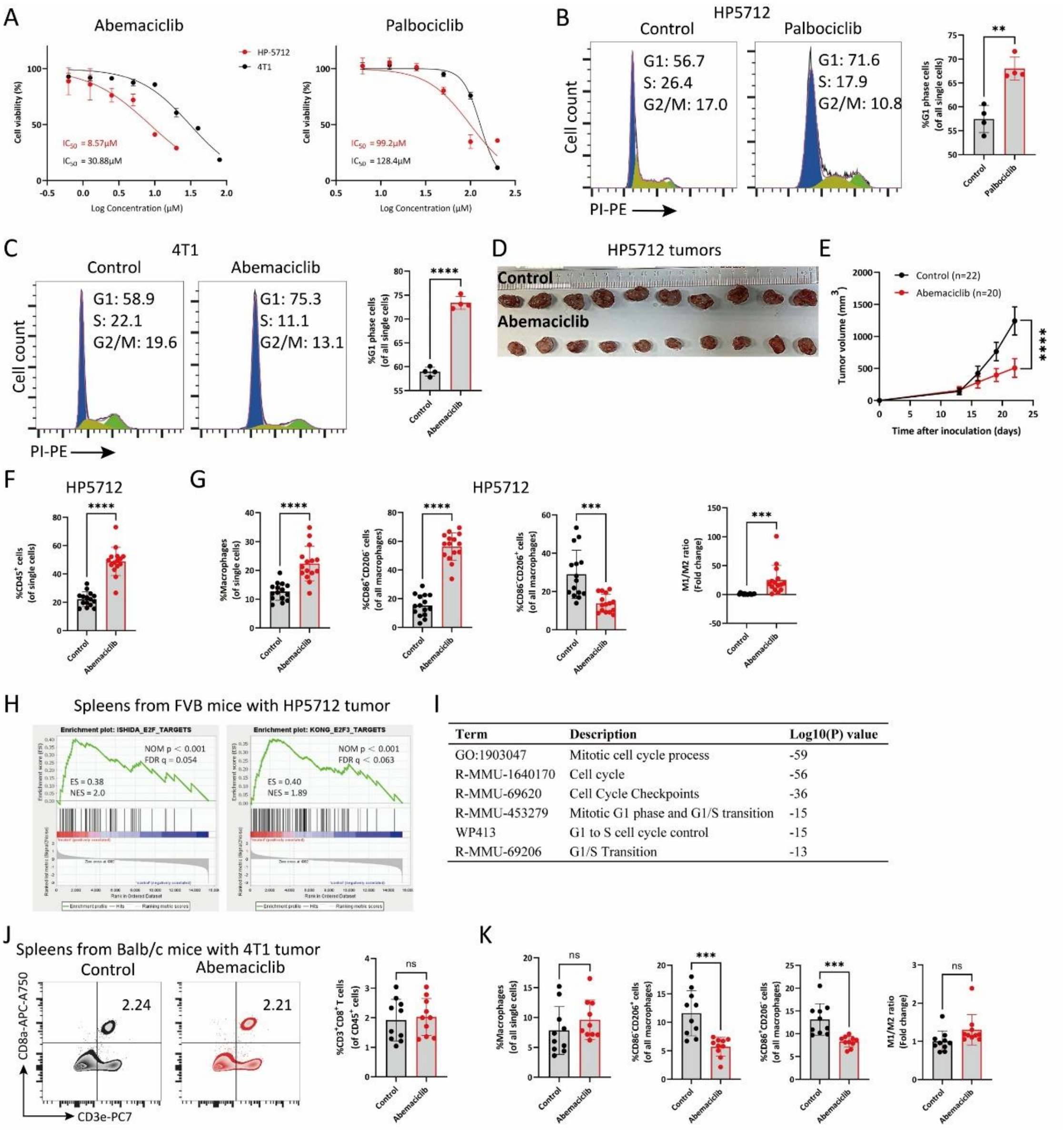

A. IC50 of CDK4/6 inhibitors, Abemaciclib (left) and Palbociclib (right), in HP5712 and 4T1 cell lines.

B-C. Representative FACS cell cycle distribution and comparison of G1 phase cell populations of HP5712 (B) and 4T1 (C) cells after control or Abemaciclib treatment (n=4, respectively).

D. Representative tumor images of FVB mice orthotopically injected with HP5712 cells after 10 days of control or Abemaciclib treatment (n=10, respectively).

E. Tumor volume changes of FVB mice orthotopically injected with HP5712 cells after 10 days of control or Abemaciclib treatment (control, n=22; Abemaciclib, n=20).

F. Intratumoral lymphocytes from FVB mice orthotopically injected with HP5712 cells after 10 days of control or Abemaciclib treatment.

G. Intratumoral macrophages, M1 phenotypes, M2 phenotypes, and fold changes of M1/M2 ratio in FVB mice orthotopically injected with HP5712 cells after 10 days of control or Abemaciclib treatment (n=15, respectively).

H-I. GSEA terms (H) and GO terms (I) significantly upregulated by Abemaciclib compared to control in spleens from FVB mice orthotopically injected with HP5712 cells (n=3, respectively).

J. Representative FACS plot and quantifications of $CD8^+$ T cells in spleens from Balb/c mice orthotopically injected with 4T1 cells after 10 days of control or Abemaciclib treatment (n=10, respectively).

K. Intratumoral macrophages, M1 phenotypes, M2 phenotypes, and fold changes of M1/M2 ratio in spleens from Balb/c mice orthotopically injected with 4T1 cells after 10 days of control or Abemaciclib treatment (n=10, respectively).

P-values are calculated using unpaired two-tailed t-tests (B, C, E-G, J, K). Data presented as mean ± SD. ns, $p > 0.05$; $^{**}p < 0.01$; $^{***}p < 0.001$; $^{****}p < 0.0001$.

Figure S2. Cell type annotation for scRNA-seq of Balb/c mice orthotopically implanted 4T1 tumors after control or Abemaciclib treatment.

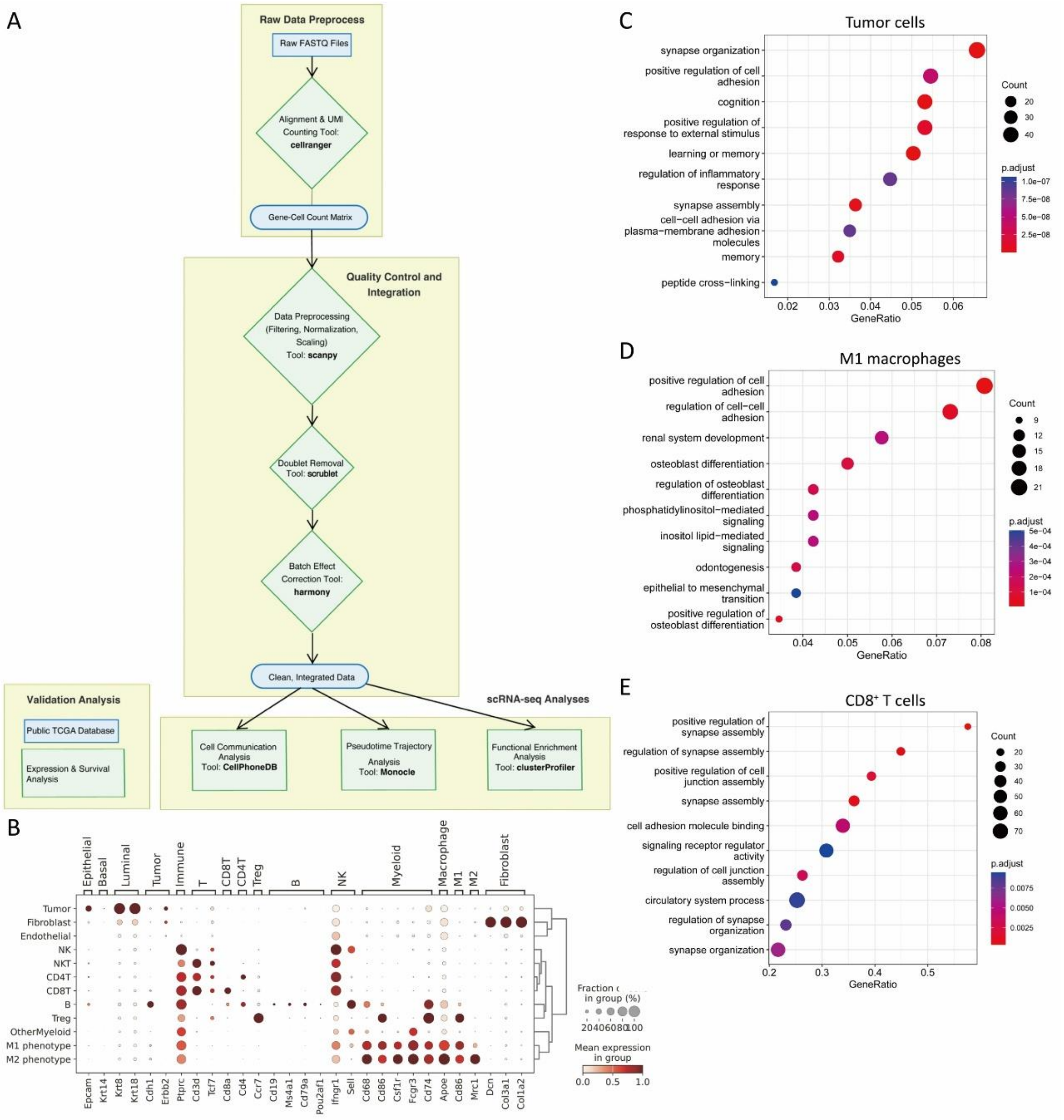

A. Schematic diagram of bioinformatics analysis strategy.

B. Cell type annotation of scRNA-seq data from tumor samples using integrated clustering and reference-based classification methods.

C-E. GO terms of Abemaciclib-upregulated genes in tumor cells (B), M1 macrophages (C), and $CD8^+$ T cells (D) compared to control.

Figure S3. CDK4/6 inhibition increases intratumoral CD8^{+} T cells and macrophages *ex vivo*.

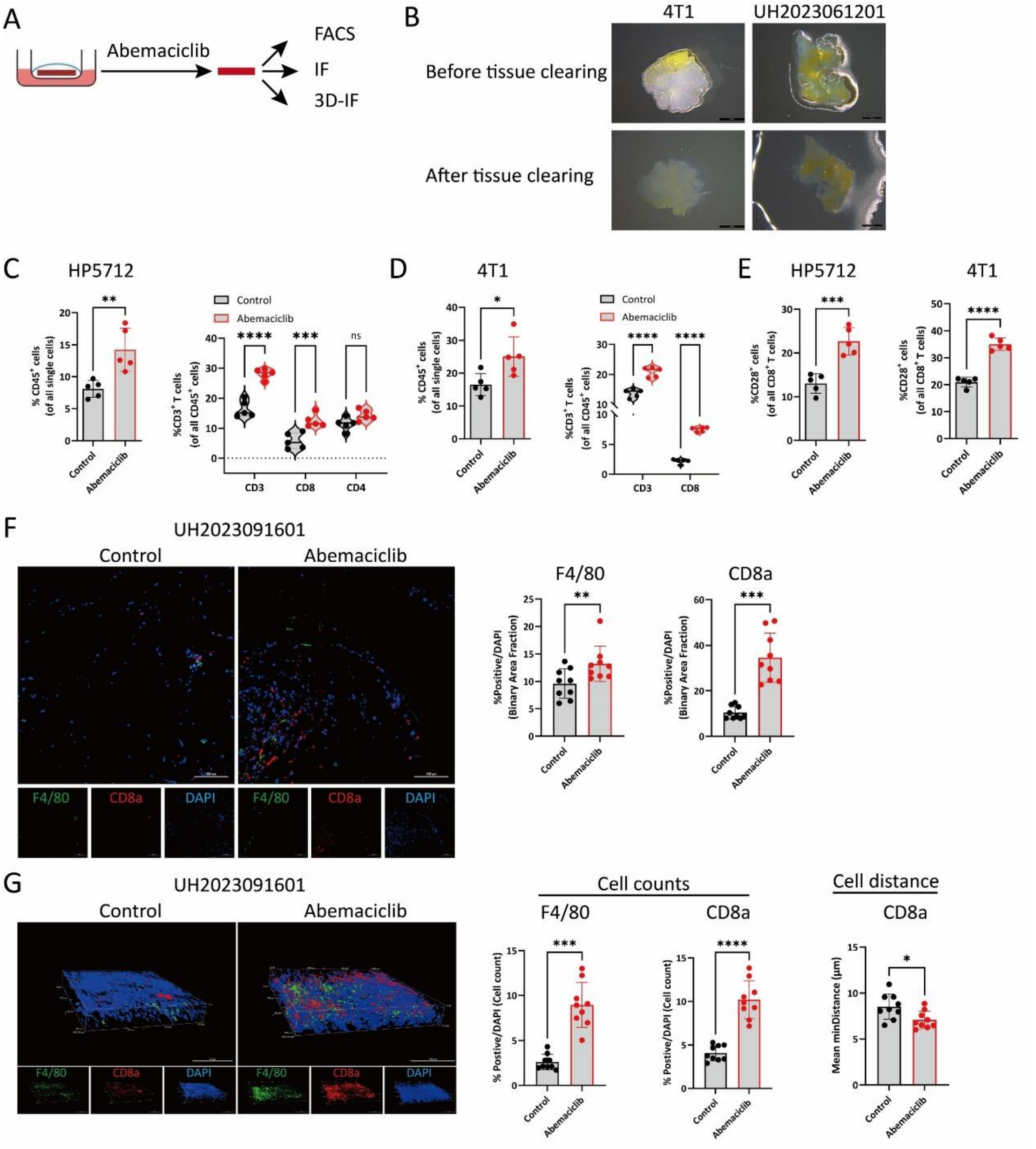

A. Workflow of assessing the tumor immune cell changes of tumor slices after Abemaciclib treatment.

B. Representative images of murine and human tumor slices before and after tissue clearing; scale bar = 2 mm.

C-D. Intratumoral lymphocytes and T cell subpopulations in immunocompetent mice orthotopically injected with HP5712 tumor slices (C) or 4T1 tumor slices (D) after 5 days of control or Abemaciclib *ex vivo* treatment (n=5, respectively).

E. CD28 expression on $CD8^+$ T cells in immunocompetent mice orthotopically injected with HP5712 (left) or 4T1 (right) tumor slices after 5 days of control or Abemaciclib treatment (n=5, respectively).

F. Representative IF images and quantifications of F4/80 (green) and CD8a (red) expression on human Luminal breast tumor slices after 5 days of control or Abemaciclib treatment (3 areas × 3 samples, respectively); scale bar = 100 μm**.**

G. Representative 3D-IF images, quantifications of F4/80-positive (green) and CD8a-positive (red) cells, and analysis of the mean minimal distance of CD8a-positive cells in human Luminal breast tumor slices after 5 days of control or Abemaciclib treatment (3 areas × 3 samples, respectively); scale bar = 100 μm**.**

P-values are calculated using unpaired two-tailed t-tests (C-G), or two-way ANOVA corrected for multiple comparisons (C, D). Data presented as mean ± SD. *$p < 0.05$; **$p < 0.01$; ***$p < 0.001$; ****$p < 0.0001$.

Figure S4. CDK4/6 inhibition induces immune-like macrophage memory in a tumor-dependent manner.

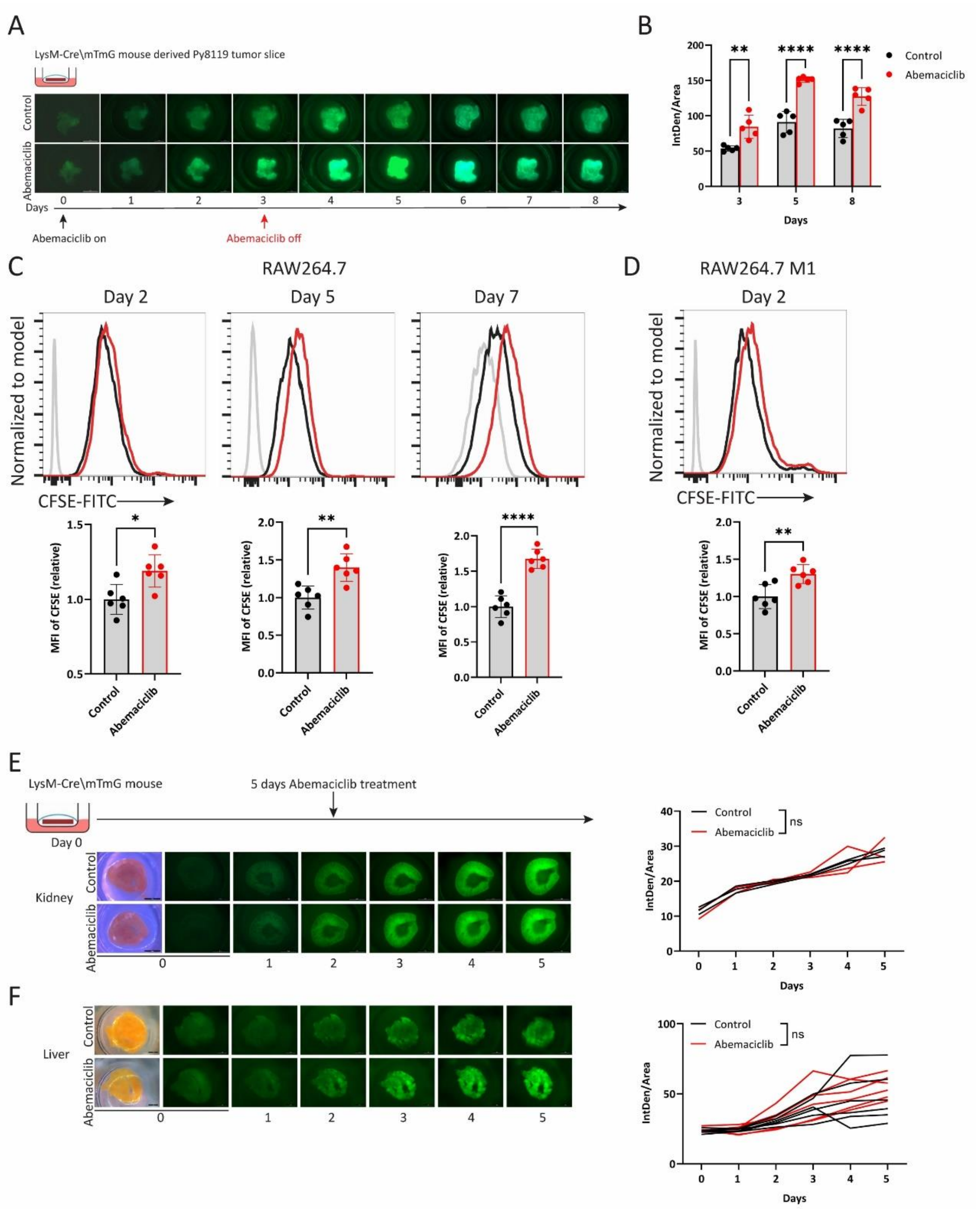

A-B. Representative image (A) and intensity quantification at different days (B) of LysM-Cre\mTmG mice orthotopically injected with Py8119 tumor slices after control or Abemaciclib treatment (n=5, respectively); scale bar = 2 mm.

C. Representative FACS histograms and quantifications of CFSE expression on the surface of RAW264.7 macrophages after 2, 5, and 7 days of control or Abemaciclib treatment (n=6, respectively).

D. Representative FACS histograms and quantifications of CFSE expression on the surface of RAW264.7 (M1) macrophages after 2 days of control or Abemaciclib treatment (n=6, respectively).

E-F. Representative images and dynamic GFP intensity changes of LysM-Cre\mTmG tumor-free mice-derived kidney (E; n=5, respectively) and liver (F; n=5, respectively) slices after 5 days of control or Abemaciclib treatment; scale bar = 2 mm.

P-values are calculated using unpaired two-tailed t-tests (C-F); two-way ANOVA corrected for multiple comparisons (B). Data presented as mean ± SD. ns, $p > 0.05$; *$p < 0.05$; ****$p < 0.0001$.

Figure S5. CDK4/6 inhibition expands intratumoral CD8⁺ T cells dependent on macrophages *ex vivo* but expands macrophages independent of CD8⁺ T cells *ex vivo*.

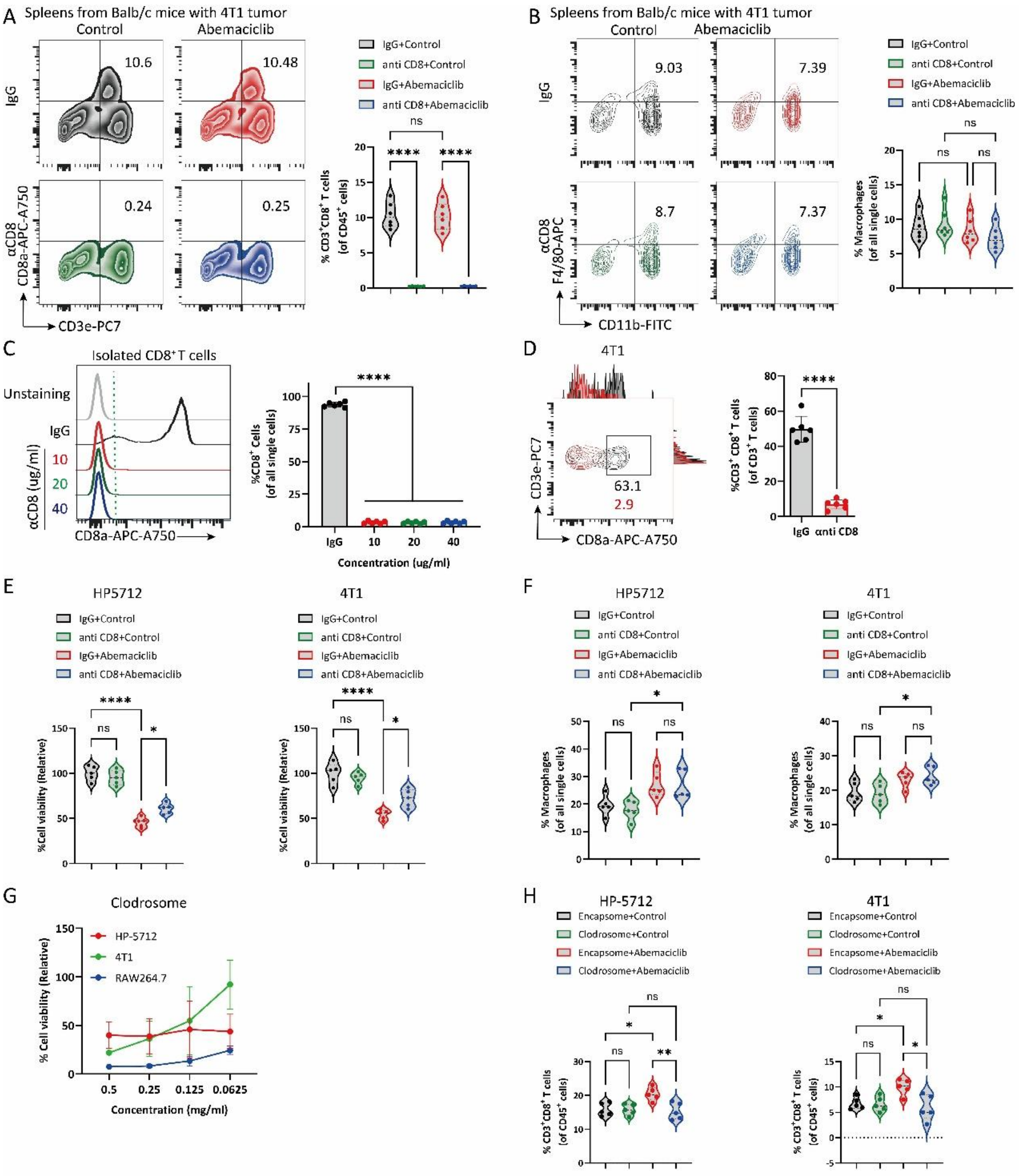

A-B. Representative FACS plots and quantifications of CD8⁺ T cells (A) and macrophages (B) in spleens from Balb/c mice orthotopically injected with 4T1 cells after IgG or anti-CD8 depletion plus control or Abemaciclib treatment (n=6, respectively).

C. CD8a expression on FVB mouse-derived naïve CD8⁺ T cells after IgG or gradient concentration of anti-CD8 depletion treatment (n=6, respectively).

D. Representative FACS plots and quantifications of $CD8^+$ T cells in immunocompetent mice orthotopically injected with 4T1 tumor slices after IgG or anti-CD8 depletion *ex vivo* treatment (n=6, respectively).

E. Quantification of cell viability in immunocompetent mice orthotopically injected with HP5712 (left; n=5, respectively) and 4T1 (right; n=5, respectively) after pre-IgG or pre-anti-CD8 plus control or Abemaciclib *ex vivo* treatment.

F. Intratumoral macrophages in immunocompetent mice orthotopically injected with HP5712 (left) and 4T1 (right) tumor slices after pre-IgG or pre-anti-CD8 plus control or Abemaciclib *ex vivo* treatment (n=5, respectively).

G. Cell viability of cancer cells and macrophages after gradient concentration of Encapsome or Clodrosome treatment (n=3, respectively).

H. Intratumoral $CD8^+$ T cells from immunocompetent mice orthotopically implanted with HP5712 (left) and 4T1 (right) tumor slices after Encapsome or Clodrosome pretreatment plus control or Abemaciclib treatment (n=5, respectively).

P-values are calculated using unpaired two-tailed t-tests (D), or one-way ANOVA corrected for multiple comparisons (A-C, E, F, H). Data presented as mean ± SD. ns, $p > 0.05$; *$p < 0.05$; **$p < 0.01$; ***$p < 0.001$; ****$p < 0.0001$.

Figure S6. CDK4/6 inhibition increases macrophage proliferation and activation and polarizes M2 to M1 macrophages dependent on the tumor microenvironment.

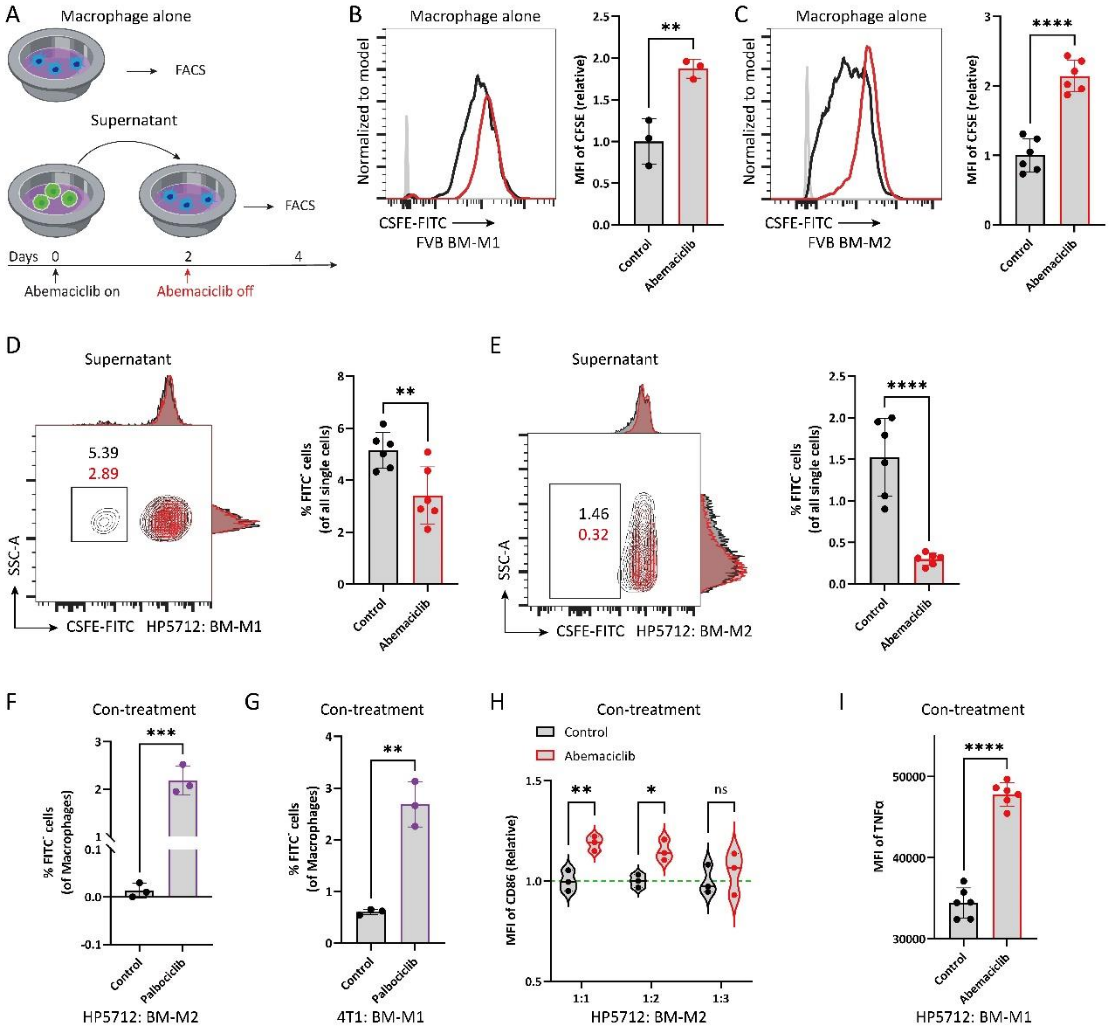

A. Macrophage alone or the supernatant model to detect macrophage proliferation and activation potential.

B-C. Representative FACS histograms and quantifications of the proliferation potential of FVB mouse BM-M1 macrophages (B; n=3, respectively) or BM-M2 macrophages (C; n=6, respectively) alone after control or Abemaciclib treatment.

D-E. Representative FACS plots and quantifications of the proliferation potential of FVB mouse BM-M1 macrophages (D; n=6, respectively) or BM-M2 macrophages (E; n=6, respectively) in the supernatant model after control or Abemaciclib treatment.

F-G. Quantifications of the proliferation potential of FVB mouse BM-M2 macrophages cocultured with HP-5712 (F; n=3, respectively) or Balb/c mouse BM-M1 macrophages

cocultured with 4T1 (G; n=3, respectively) after control or Palbociclib treatment in the con-treatment model.

H. Fold changes of CD86 expression on the surface of FVB mouse BM-M2 macrophages cocultured with HP5712 at different ratios after control or Abemaciclib treatment in the con-treatment model (n=3, respectively).

I. Quantifications of TNFα expression of FVB mouse BM-M1 macrophages cocultured with HP5712 after control or Abemaciclib treatment in the con-treatment model (n=6, respectively).

P-values are calculated using unpaired two-tailed t-tests (B-G, I), two-way ANOVA corrected for multiple comparisons (H). Data presented as mean ± SD. *$p < 0.05$; **$p < 0.01$; ***$p < 0.001$; ****$p < 0.0001$.

Figure S7. CDK4/6 inhibition-trained M1 TAM supernatant fails to stimulate CD8$^+$ T cell antitumor immunity without cell-cell interactions.

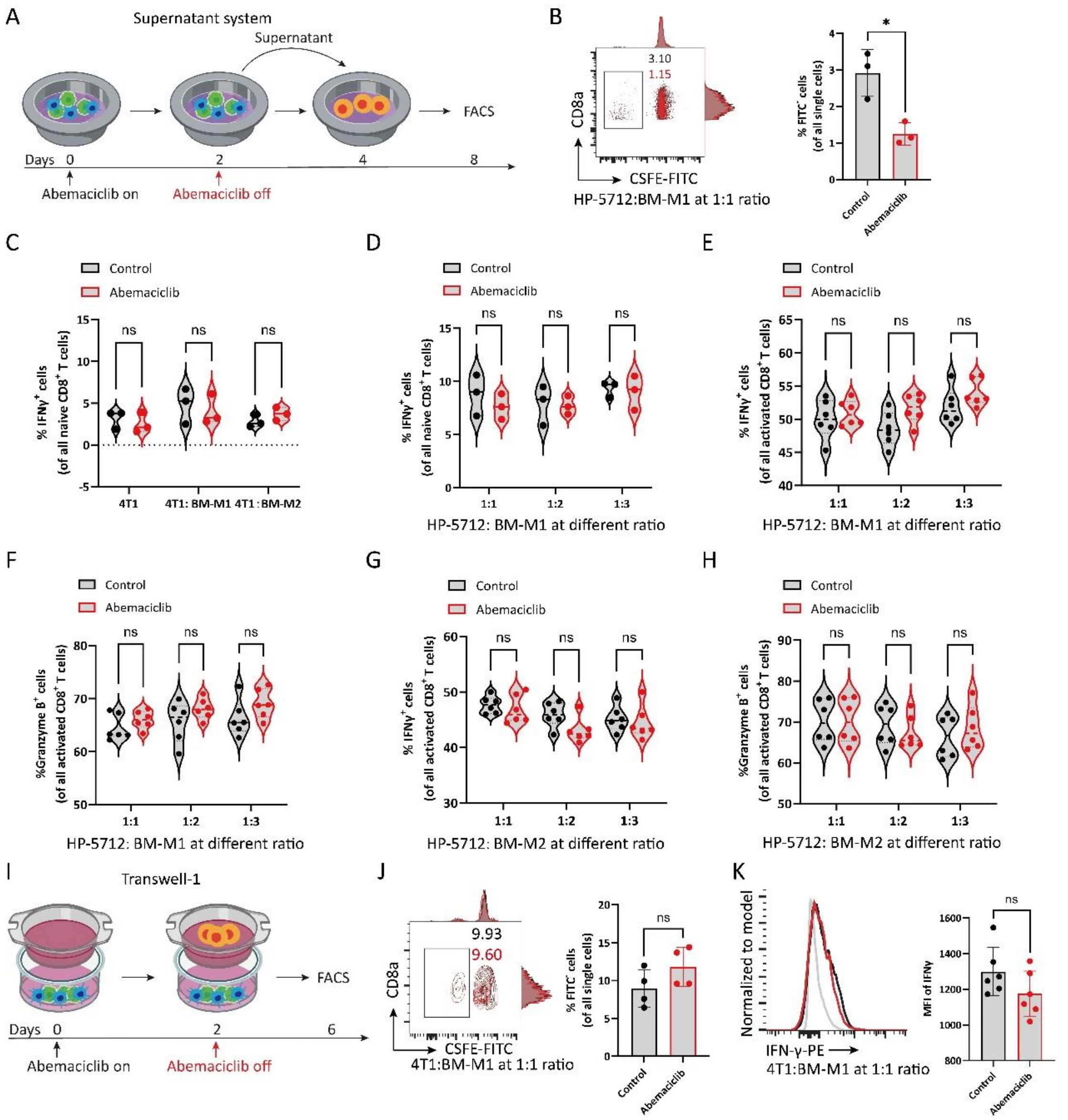

A. The supernatant model to detect CD8$^+$ T cell proliferation and activation.

B. Representative FACS plots and quantifications of the proliferation potential of Balb/c mouse-derived naïve CD8$^+$ T cells in the supernatant model after control or Abemaciclib treatment (n=3, respectively).

C. Quantifications of IFNγ expression on the surface of FVB mouse-derived naïve CD8$^+$ T cells in the supernatant model of HP5712 cocultured with FVB mouse BM-M1 macrophages at different ratios after control or Abemaciclib treatment (n=3, respectively).

D. Quantifications of IFNγ expression on the surface of Balb/c mouse-derived naïve $CD8^+$ T cells in the supernatant model of 4T1 alone, 4T1 cocultured with Balb/c mouse BM-M1 or BM-M2 macrophages at a 1:1 ratio after control or Abemaciclib treatment (n=3, respectively).

E-F. Quantifications of IFNγ (E; n=6, respectively) and granzyme B (F; n=6, respectively) expression on the surface of FVB mouse-derived activated $CD8^+$ T cells in the supernatant model of HP5712 cocultured with FVB mouse BM-M1 macrophages at different ratios after control or Abemaciclib treatment.

G-H. Quantifications of IFNγ (G; n=6, respectively) and granzyme B (H; n=6, respectively) expression on the surface of FVB mouse-derived activated $CD8^+$ T cells in the supernatant model of HP5712 cocultured with FVB mouse BM-M2 macrophages at different ratios after control or Abemaciclib treatment.

I. The Transwell model to detect $CD8^+$ T cell proliferation and activation.

J. Representative FACS plots and quantifications of the proliferation potential of Balb/c mouse-derived naïve $CD8^+$ T cells in the Transwell model after control or Abemaciclib treatment (n=3, respectively).

K. Representative FACS histograms and quantifications of IFNγ expression on the surface of Balb/c mouse-derived activated $CD8^+$ T cells in the supernatant model of 4T1 cocultured with Balb/c mouse BM-M1 macrophages at a 1:1 ratio after control or Abemaciclib treatment.

P-values are calculated using unpaired two-tailed t-tests (B, J, K), or two-way ANOVA corrected for multiple comparisons (C-H). Data presented as mean ± SD. ns, $p > 0.05$; $*p < 0.05$.

Figure S8. CDK4/6 inhibition-trained M1 TAMs penetrate into tumor slices and remodel the tumor immune microenvironment.

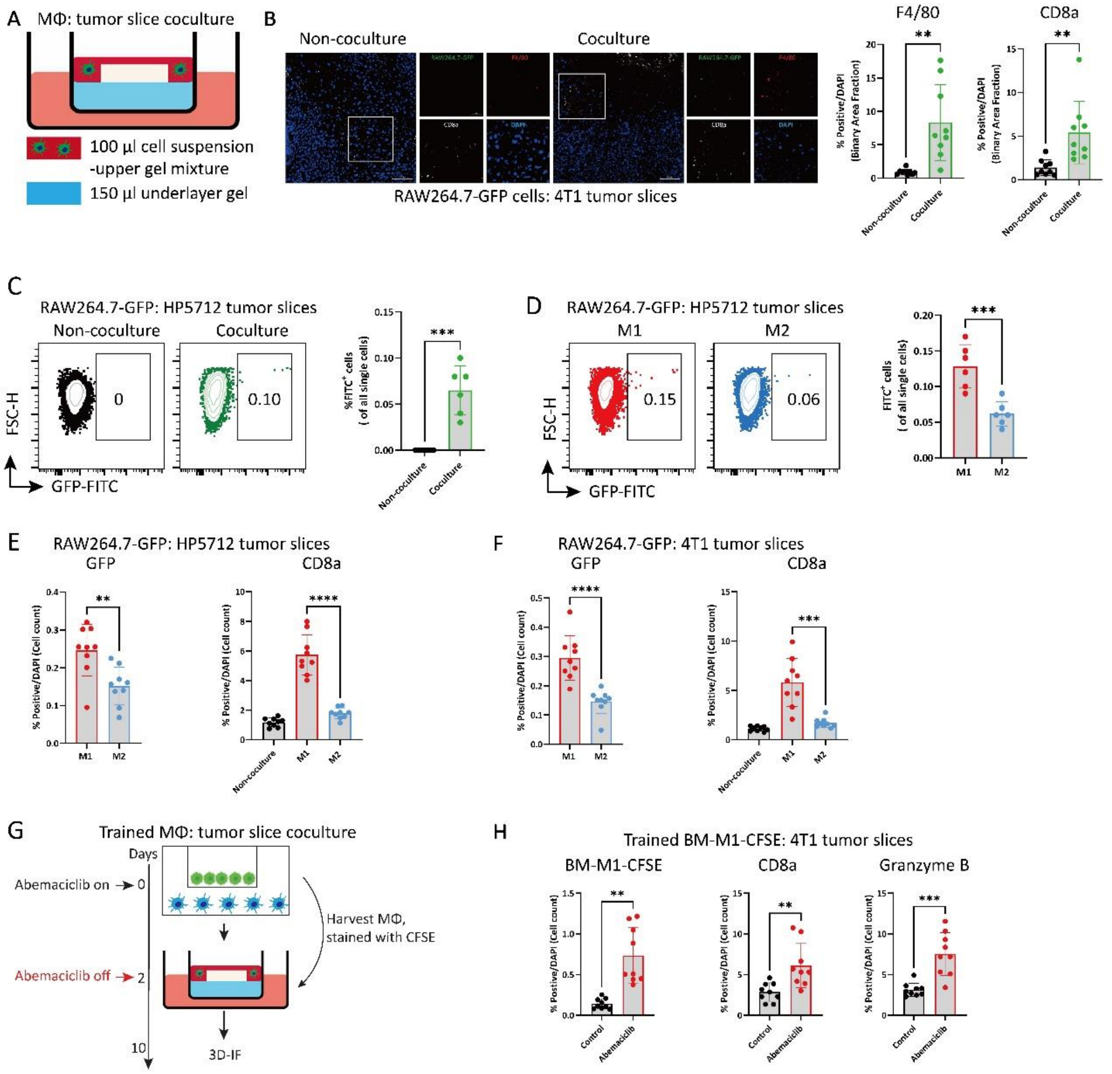

A. Coculture model of macrophages and tumor slices.

B. Representative IF images and quantifications of penetrated RAW264.7-GFP (green) and F4/80 (red) and CD8a (white) expression on the surfaces of Balb/c mice orthotopically injected with 4T1 tumor slices after 8 days of coculture (3 areas × 3 samples, respectively).

C. Representative FACS plots and quantifications of RAW264.7-GFP penetration capacity in FVB mice orthotopically injected with HP5712 tumor slices after 8 days of coculture (n=6, respectively).

D. Penetration capacity of different RAW264.7-GFP subpopulations in FVB mice orthotopically injected with HP5712 tumor slices after 8 days of coculture (n=6, respectively).

E-F. Quantification of penetration capacity of RAW264.7-GFP M1 or M2 macrophages and CD8a expression in immunocompetent mice orthotopically injected with HP5712 (E; 3 areas × 3 samples, respectively) or 4T1 (F; 3 areas × 3 samples, respectively) tumor slices after 8 days of coculture.

G. Workflow to detect penetration capacity of Abemaciclib-trained immunocompetent mouse BM-M1 macrophages in tumor slices and changes in intratumoral immune cell repertoire after coculture.

H. Quantification of BM-M1-CFSE-positive, CD8a-positive, and granzyme B-positive cells in Balb/c mice orthotopically injected with 4T1 tumor slices cocultured with control or Abemaciclib-trained BM-M1-CFSE (3 areas × 3 samples, respectively).

P-values are calculated using unpaired two-tailed t-tests (B-F, H), or one-way ANOVA corrected for multiple comparisons (E, F). Data presented as mean ± SD. *$p < 0.05$; **$p < 0.01$; ***$p < 0.001$; ****$p < 0.0001$.

Figure S9. CDK4/6 inhibition fails to reprogram TAMs and trigger CD8$^+$ T cell antitumor immunity in CDK4/6-resistant tumor cells.

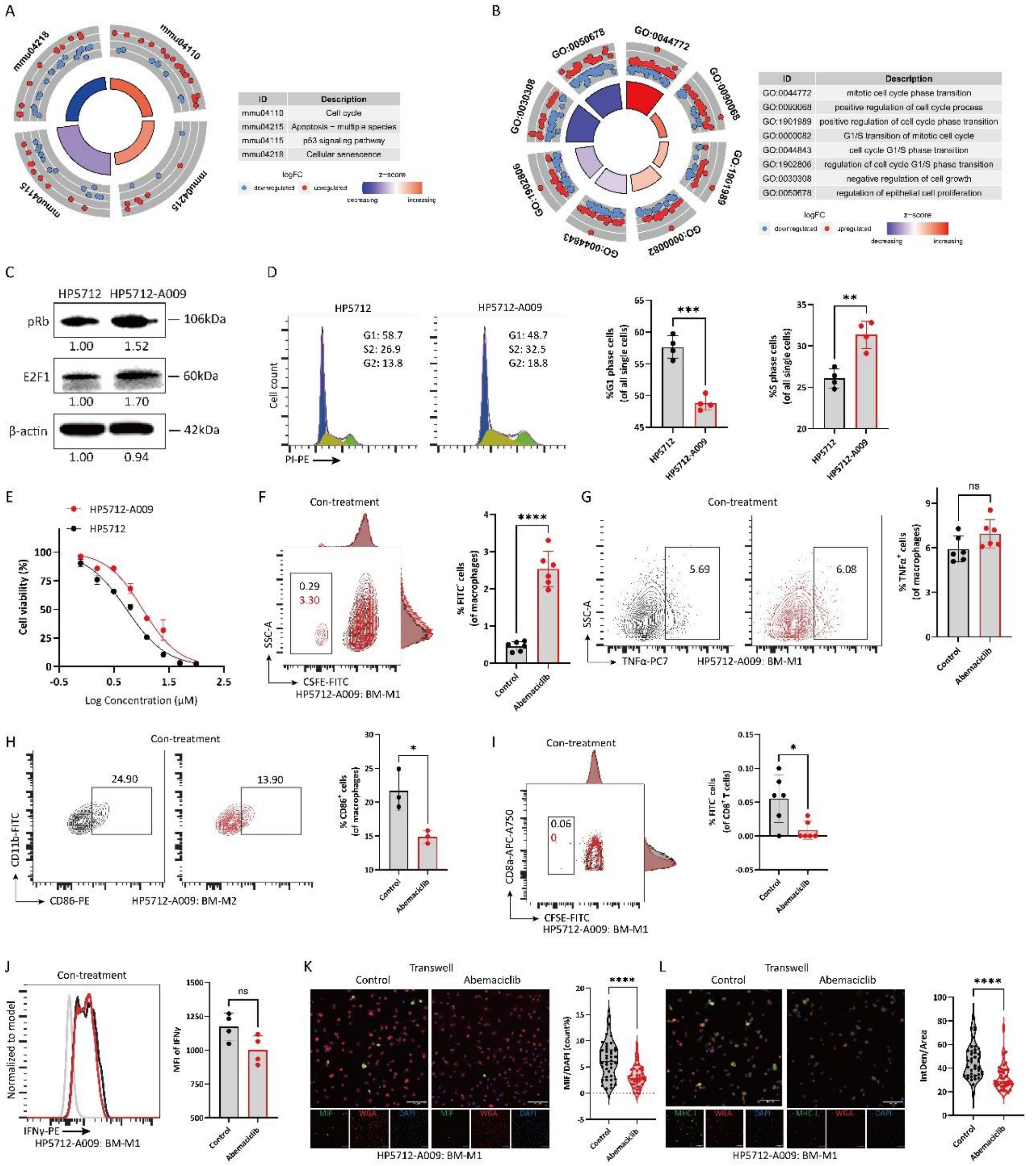

A-B. KEGG terms (A) and GO terms (B) of significantly upregulated and downregulated genes in HP5712-A009 cells compared to HP5712 cells during the log phase (n=3, respectively).

C. Representative immunoblots of pRb and E2F1 expression in HP5712-A009 and HP5712 cells during the log phase.

D. Representative FACS cell cycle distribution and quantifications of G_1 and S phase cell populations between HP5712-A009 and HP5712 during the log phase (n=4, respectively).

E. Cell viability of HP5712-A009 and HP5712 cells at the log phase after gradient concentrations of Abemaciclib treatments (n=3, respectively).

F-H. Representative FACS plots and quantifications of proliferation potential of FVB mouse BM-M1 macrophages (F; n=6, respectively), TNFα expression on the surface of FVB mouse BM-M1 macrophages (G; n=6, respectively), and CD86 expression on the surface of FVB mouse BM-M2 macrophages (H; n=3, respectively) cocultured with HP5712-A009 cells after control or Abemaciclib treatment in the con-treatment model.

I. Representative FACS plots and quantifications of proliferation potential of FVB mouse-derived naïve $CD8^+$ T cells in the coculture system of HP5712-A009: FVB mouse BM-M1 macrophages at a 1:1 ratio after vehicle or Abemaciclib treatment (n=6, respectively).

J. Representative FACS histograms and quantifications of IFNγ expression on FVB mouse-derived activated $CD8^+$ T cells in the coculture system of HP5712-A009: FVB mouse BM-M1 macrophages at a 1:1 ratio after vehicle or Abemaciclib treatment (n=4, respectively).

K. Representative images and quantifications of MIF expression (green) in control- or Abemaciclib-treated FVB mouse BM-M1 macrophage from HP5712-A009: BM-M1 in the Transwell model (10 areas × 4 samples, respectively); scale bar = 100 μm.

L. Representative images and quantifications of MHC-I expression (green) in control- or Abemaciclib-treated FVB mouse BM-M1 macrophages from HP5712-A009: BM-M1 in the Transwell model (10 areas × 4 samples, respectively); scale bar = 100 μm.

P-values are calculated using unpaired two-tailed t-tests (D, F-L). Data presented as mean ± SD. ns, $p > 0.05$; *$p < 0.05$; **$p < 0.01$; ***$p < 0.001$; ****$p < 0.0001$.

Figure S10. Tumor cell-secreted MIF is important to CDK4/6 inhibition reprogramming TAMs.

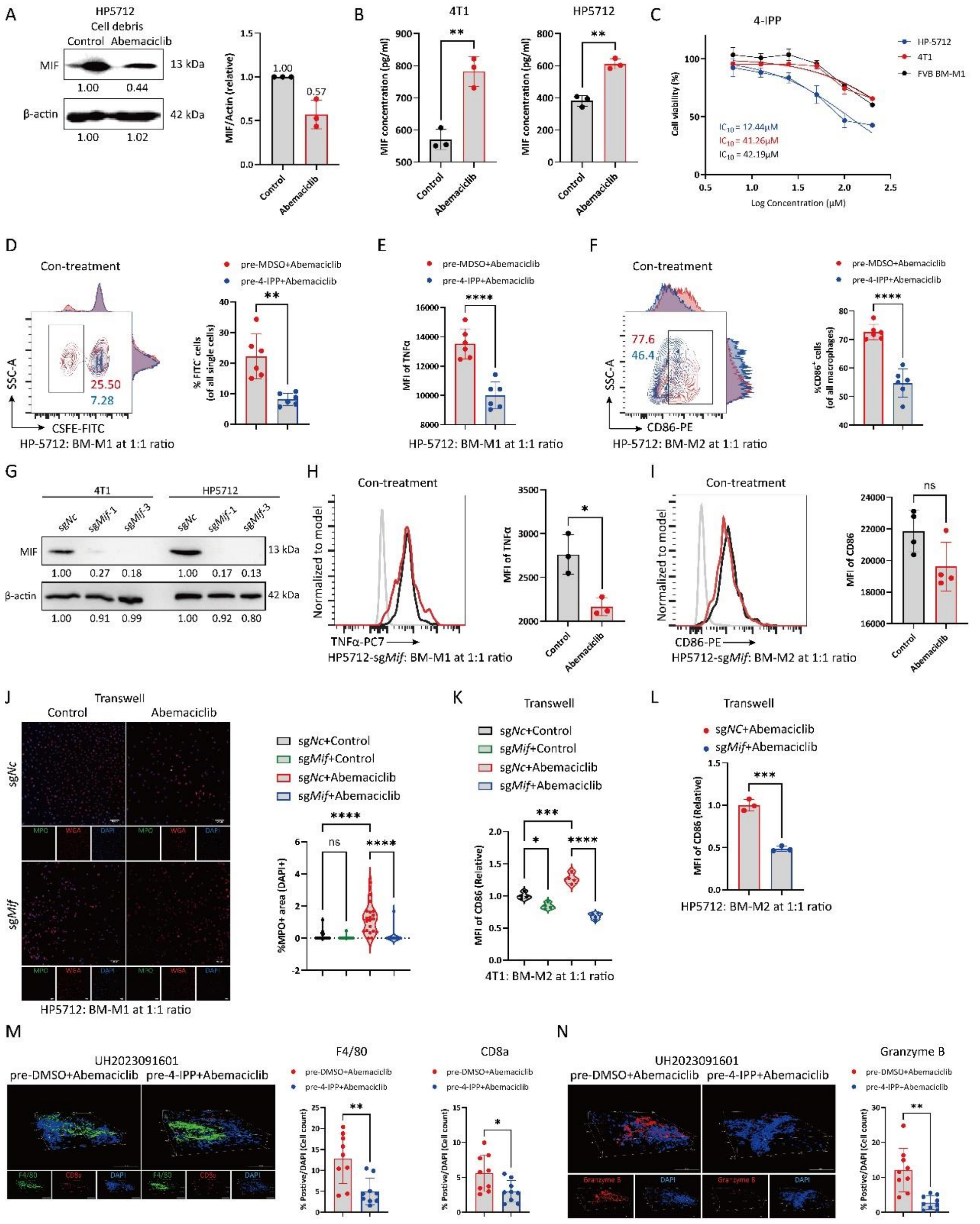

A. Representative immunoblots and fold changes of MIF expression in HP5712 cell debris after control or Abemaciclib treatment (n=3, respectively).

B. Secreted MIF concentration in the supernatant from 4T1 and HP5712 cells after control or Abemaciclib treatment (n=3, respectively).

C. IC10 of 4-IPP on HP5712, 4T1, and FVB mouse BM-M1 macrophages (n=3, respectively).

D. Representative FACS plots and quantifications of the proliferation potential of FVB mouse BM-M1 macrophages cocultured with HP5712 after pre-DMSO plus Abemaciclib or pre-4-IPP plus Abemaciclib treatment in the con-treatment model (n=6, respectively).

E. Quantifications of TNFα expression on the surface of FVB mouse BM-M1 macrophages cocultured with HP5712 after pre-DMSO plus Abemaciclib or pre-4-IPP plus Abemaciclib treatment in the con-treatment model (n=6, respectively).

F. Representative FACS plots and quantifications of CD86 expression on the surface of FVB mouse BM-M1 macrophages cocultured with HP5712 after pre-DMSO plus Abemaciclib or pre-4-IPP plus Abemaciclib treatment in the con-treatment model (n=6, respectively).

G. Knockout efficacy of two different sg*Mif* on MIF expression in 4T1 (left) and HP5712 (right) cell lines.

H. Representative FACS histograms and quantifications of TNFα expression on the surface of FVB mouse BM-M1 macrophages cocultured with HP5712-sg*Mif* after control or Abemaciclib treatment in the con-treatment model (n=3, respectively).

I. Representative FACS histograms and quantifications of CD86 expression on the surface of FVB mouse BM-M2 macrophages cocultured with HP5712-sg*Mif* after control or Abemaciclib treatment in the con-treatment model (n=4, respectively).

J. Representative images and quantifications of MPO expression (green) in FVB mouse BM-M1 macrophages cocultured with HP5712-sg*Nc* or HP5712-sg*Mif* after control or Abemaciclib treatment in the Transwell model (5 areas × 4 samples, respectively); scale bar=100μm.

K. Quantifications of CD86 expression on the surface of Balb/c mouse BM-M2 macrophages cocultured with 4T1-sg*Nc* or 4T1-sg*Mif* after control or Abemaciclib treatment in the Transwell model (n=4, respectively).

L. Quantifications of CD86 expression on the surface of FVB mouse BM-M2 macrophages cocultured with HP5712-sg*Nc* or HP5712-sg*Mif* after Abemaciclib treatment in the Transwell model (n=3, respectively).

M-N. Representative 3D-IF images and quantifications of F4/80-positive (green) and CD8a-positive (red) (M) and granzyme B-positive (red) (N) cells in human Luminal breast tumor slices after pre-DMSO plus Abemaciclib or pre-4-IPP plus Abemaciclib treatment (3 areas × 3 samples, respectively); scale bar = 100μm.

P-values are calculated using unpaired two-tailed t-tests (B, D-F, H, I, L-N), or one-way ANOVA corrected for multiple comparisons (J, K). Data presented as mean ± SD. ns, $p > 0.05$; *$p < 0.05$; **$p < 0.01$; ***$p < 0.001$; ****$p < 0.0001$.

Figure S11. CDK4/6i activates the HIF-1 pathway in tumor cells and CD74 deletion on macrophages blocks the functional reprogramming of TAMs by CDK4/6 inhibition.

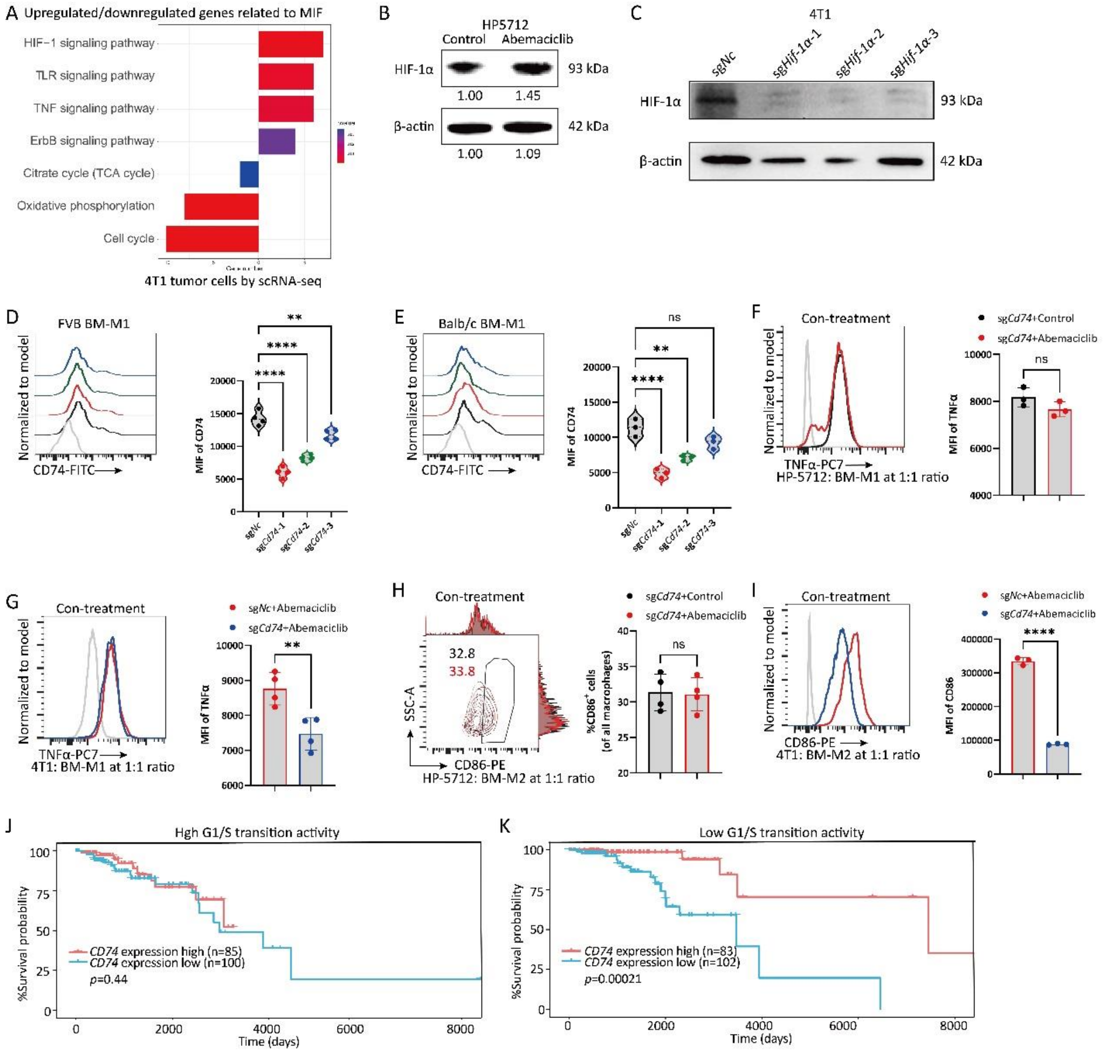

A. GO terms of Abemaciclib-upregulated/downregulated genes positively correlated with MIF in 4T1 tumor cells by scRNA-seq of Balb/c mice orthotopically implanted with 4T1 tumors.

B. Representative immunoblots and quantifications of HIF-1α expression in control- or Abemaciclib-treated HP5712 cell debris.

C. Knockout efficacy of three different sg*Hif-1α* on HIF-1α expression in 4T1 cell lines.

D-E. Knockout efficacy of three different sg*Cd74* on CD74 expression in FVB (A; n=4, respectively) and Balb/c (B; n=3, respectively) mice BM-M1 macrophages.

F. Representative FACS histograms and quantifications of TNFα expression on the surface of FVB mouse BM-M1-sg*Cd74* macrophages cocultured with HP5712 after control or Abemaciclib treatment in the con-treatment model (n=3, respectively).

G. Representative FACS histograms and quantifications of TNFα expression on the surface of Balb/c mouse BM-M1-sg*Nc* or sg*Cd74* macrophages cocultured with 4T1 after Abemaciclib treatment in the con-treatment model (n=4, respectively).

H. Representative FACS plots and quantifications of CD86 expression on the surface of FVB mouse BM-M2-sg*Cd74* macrophages cocultured with HP5712 after control or Abemaciclib treatment in the con-treatment model (n=4, respectively).

I. Representative FACS histograms and quantifications of CD86 expression on the surface of Balb/c mouse BM-M2-sg*Nc* or sg*Cd74* macrophages cocultured with 4T1 after Abemaciclib treatment in the con-treatment model (n=3, respectively).

J-K. TCGA analysis of survival benefits between *CD74*-high and *CD74*-low breast cancer patients in high G_1/S transition activity cohort (G) and low G_1/S transition activity cohort (H), respectively.

P-values are calculated using unpaired two-tailed t-tests (G-I); one-way ANOVA corrected for multiple comparisons (D-E). Data presented as mean ± SD. ns, $p > 0.05$; ** $p < 0.01$; **** $p < 0.0001$.

Figure S12. CDK4/6 inhibition-induced MIF dominates MHC-I antigen presentation machinery on M1 TAMs.

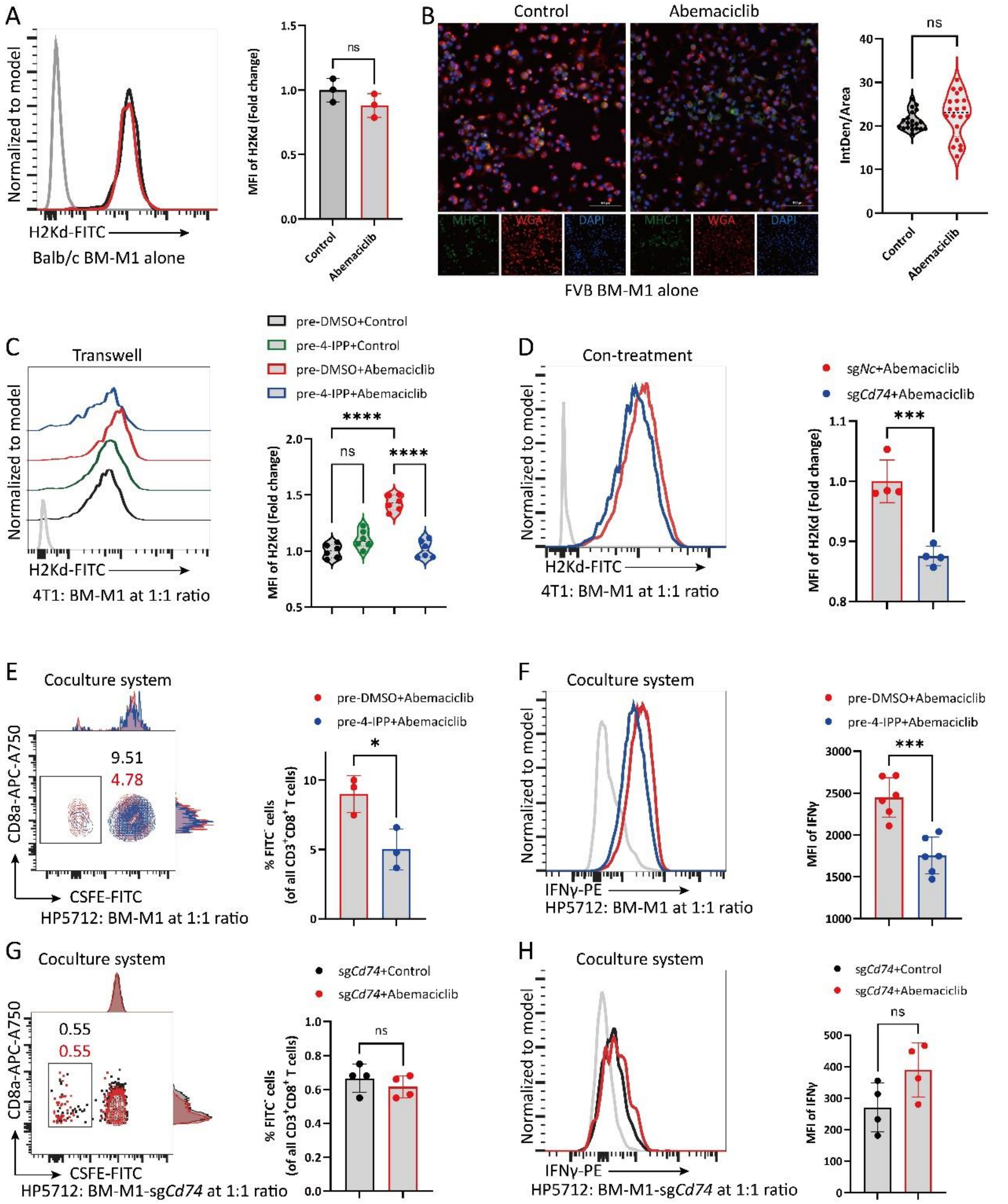

A. Representative FACS histograms and quantifications of H2Kd expression on Balb/c mouse BM-M1 macrophages after control or Abemaciclib treatment (n=3, respectively).

B. Representative images and quantifications of MHC-I expression (green) on FVB mouse BM-M1 macrophages after control or Abemaciclib treatment (5 areas × 4 samples, respectively); scale bar = 100μm.

C. Representative FACS histograms and quantifications of H2Kd expression on Balb/c mouse BM-M1 macrophages cocultured with 4T1 after pre-DMSO or 4-IPP plus control or Abemaciclib treatment in the Transwell model (n=6, respectively).

D. Representative FACS histograms and quantifications of H2Kd expression on Balb/c mouse BM-M1-sg*Nc* or sg*Cd74* macrophages cocultured with 4T1 after Abemaciclib treatment in the con-treatment model (n=4, respectively).

E. Representative FACS plots and quantifications of the proliferation potential of FVB mouse-derived naïve $CD8^+$ T cells in the coculture system of HP5712: FVB mouse BM-M1 macrophages at a 1:1 ratio after pre-DMSO plus Abemaciclib or 4-IPP plus Abemaciclib treatment (n=3, respectively).

F. Representative FACS histograms and quantifications of IFNγ expression on FVB mouse-derived activated $CD8^+$ T cells in the coculture system of HP5712: FVB mouse BM-M1 macrophages at a 1:1 ratio after pre-DMSO plus Abemaciclib or 4-IPP plus Abemaciclib treatment (n=6, respectively).

G. Representative FACS plots and quantifications of the proliferation potential of FVB mouse-derived naïve $CD8^+$ T cells in the coculture system of HP5712: FVB mouse BM-M1-sg*Cd74* macrophages at a 1:1 ratio after control or Abemaciclib treatment (n=4, respectively).

H. Representative FACS histograms and quantifications of IFNγ expression on FVB mouse-derived activated $CD8^+$ T cells in the coculture system of HP5712: FVB mouse BM-M1-sg*Cd74* macrophages at a 1:1 ratio after control or Abemaciclib treatment (n=4, respectively).

P-values are calculated using unpaired two-tailed t-tests (A, B, D-H); one-way ANOVA corrected for multiple comparisons (C). Data presented as mean ± SD. ns, $p > 0.05$; $*p < 0.05$; $**p < 0.01$; $***p < 0.001$; $****p < 0.0001$.

Figure S13. CDK4/6 inhibition-trained M1 TAM supernatant therapy augments tumor response to PD-1 ICB therapy *ex vivo*.

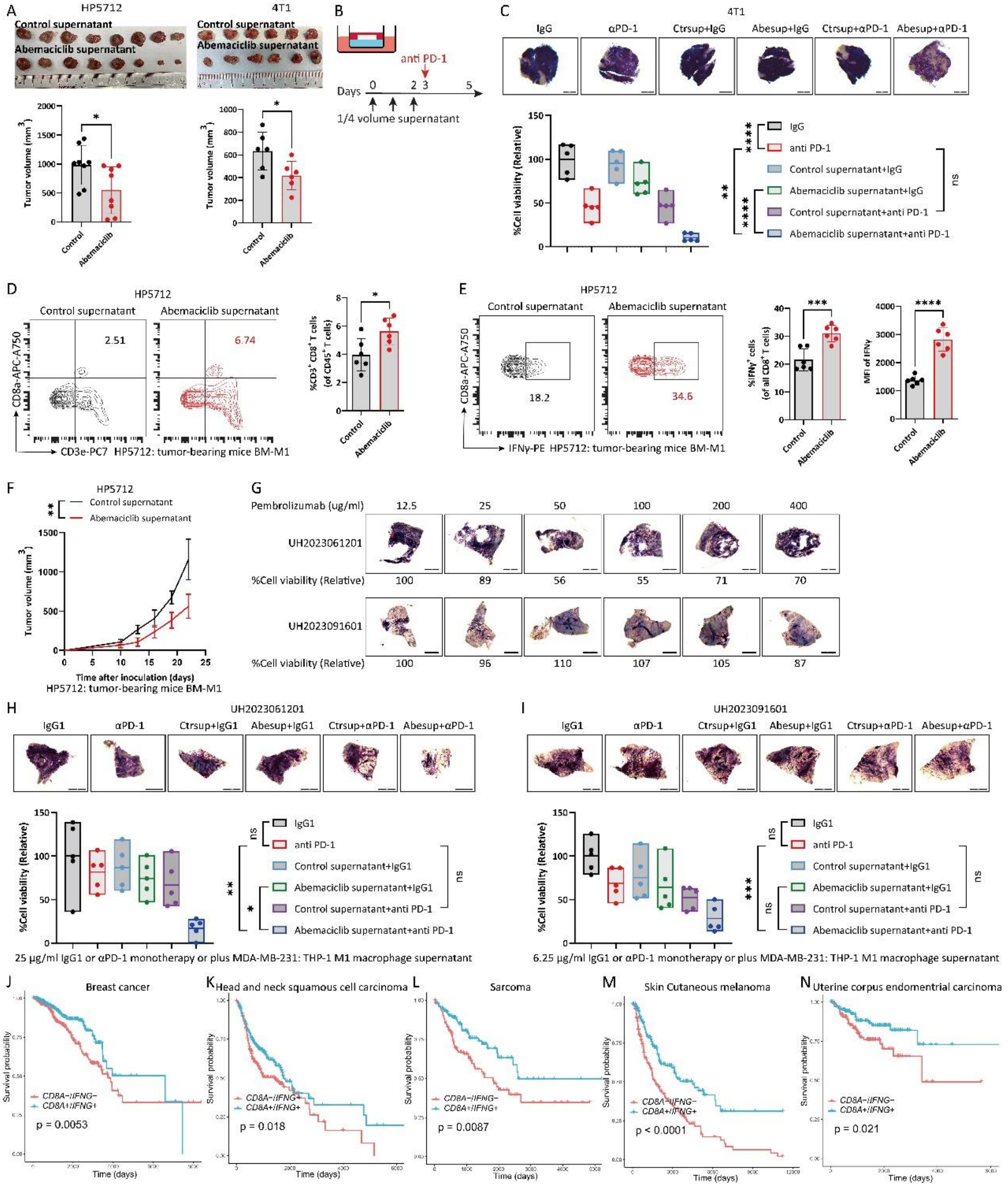

A. Representative tumor images and tumor volume comparison of FVB mice orthotopically injected with HP5712 cells (left; n=8, respectively) and Balb/c mice orthotopically injected with 4T1 cells (right; n=6, respectively) after 7 days of control supernatant or Abemaciclib supernatant therapy.

B. Workflow of CDK4/6 inhibition-trained M1 TAM supernatant therapy combined with PD-1 ICB therapy *ex vivo*.

C. Representative MTT images and quantification of cell viability in Balb/c mice orthotopically injected with 4T1 tumor slices after IgG or αPD-1 alone or combined with control supernatant or Abemaciclib supernatant therapy (n=6, respectively); scale bar=2mm.

D. Representative FACS plot and quantifications of intratumoral $CD8^+$ T cells from FVB mice orthotopically injected with HP5712 cells after control supernatant or Abemaciclib supernatant therapy (n=6, respectively).

E. Representative FACS plot and quantifications of IFNγ expression on the surface of intratumoral $CD8^+$ T cells from FVB mice orthotopically injected with HP5712 cells after control supernatant or Abemaciclib supernatant therapy (n=6, respectively).

F. Tumor volume changes of FVB mice orthotopically injected with HP5712 cells after 7 days of control supernatant or Abemaciclib supernatant therapy (n=6, respectively); D-F, the BM-M1 macrophages were derived from FVB mice orthotopically injected with HP5712 cells at day 7.

G. Tumor response of human TNBC and Luminal breast tumor slices to gradient concentrations of Pembrolizumab; scale bar=2mm.

H. Representative MTT images and quantification of cell viability in human TNBC slices after 25 μg/ml IgG1 or Pembrolizumab alone or combined with control supernatant or Abemaciclib supernatant therapy (n=5, respectively); scale bar = 2mm.

I. Representative MTT images and quantification of cell viability in human Luminal breast tumor slices after 6.25 μg/ml IgG1 or Pembrolizumab alone or combined with control supernatant or Abemaciclib supernatant therapy (n=5, respectively); H-I, the supernatant was collected from MDA-MB-231 cells cocultured with THP1 M1 macrophages at a 1:1 ratio after control or Abemaciclib treatment; scale bar = 2mm.

J-N. TCGA analysis of survival benefits in patients with breast cancer, head and neck squamous cell carcinoma, sarcoma, skin cutaneous melanoma, and uterine corpus endometrial carcinoma based on *CD8A* and *IFNG* expression status (either *CD8A−*/*IFNG−* or *CD8A+*/ *IFNG+*).

P-values are calculated using unpaired two-tailed t-tests (A, D-F); one-way ANOVA corrected for multiple comparisons (C, H-I). Data presented as mean ± SD. ns, $p > 0.05$; *$p < 0.05$; **$p < 0.01$; ***$p < 0.001$; ****$p < 0.0001$.

Supplementary Tables

Table S1. Clinical pathological information of human breast cancer.

ID	Gender	Age	TNM	Location	Surgery	Molecular Phenotype
UH2023061201	Female	68	pT1aN0M0	Left	Mastectomy	TNBC
UH2023091601	Female	37	pT1N0M0	Right	Mastectomy	Luminal-HER2-

Gating strategies for flow cytometry

Supplementary Methods 1. Gating strategy for T cell populations and CD28 or IFNγ expressions on CD8+ T cells.

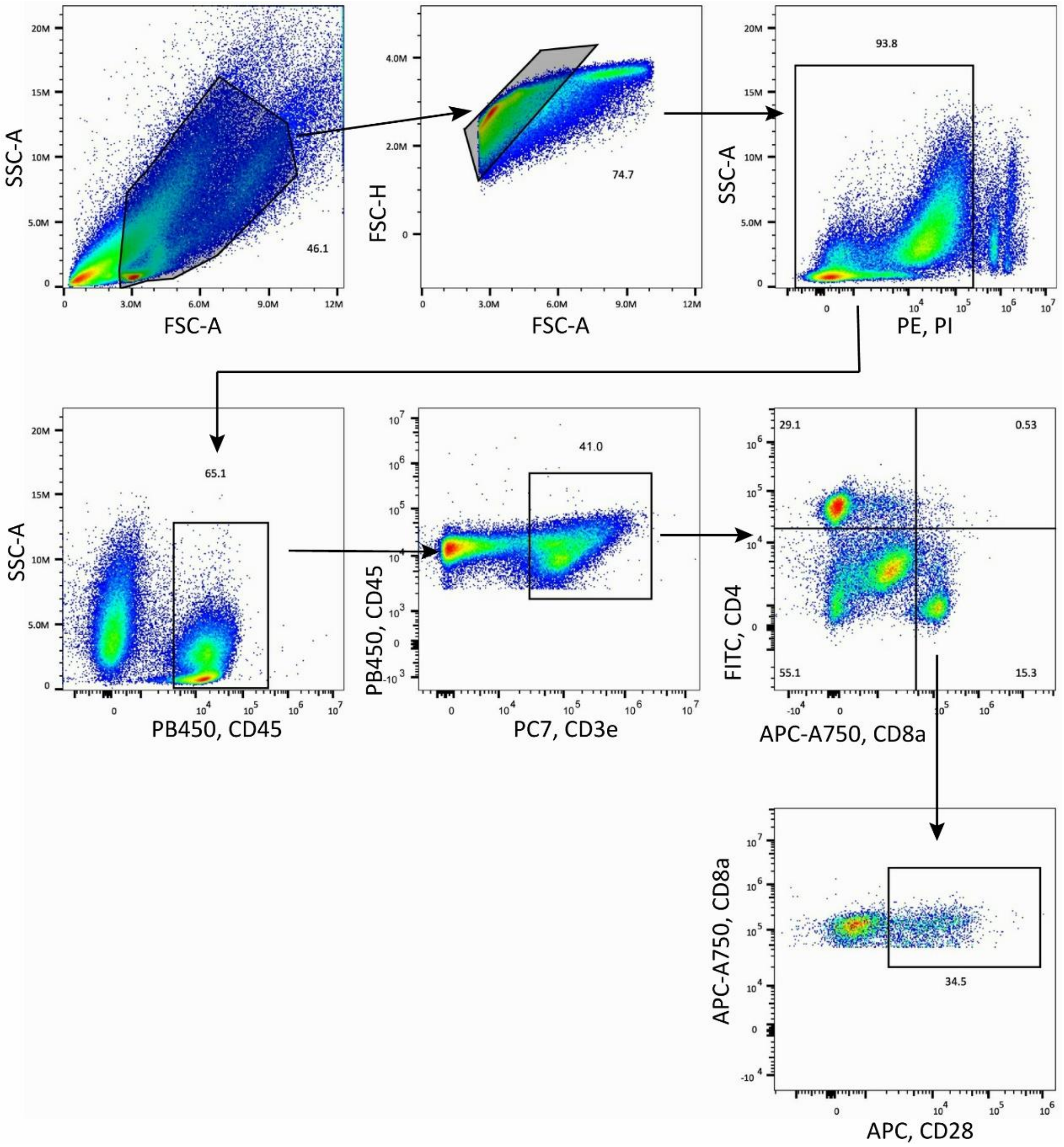

Flow cytometry gating strategy for T cell populations (CD4+ and CD8+ T cells) in spleen, tumors, and tumor slices.

Supplementary Methods 2. Gating strategy for macrophage populations.

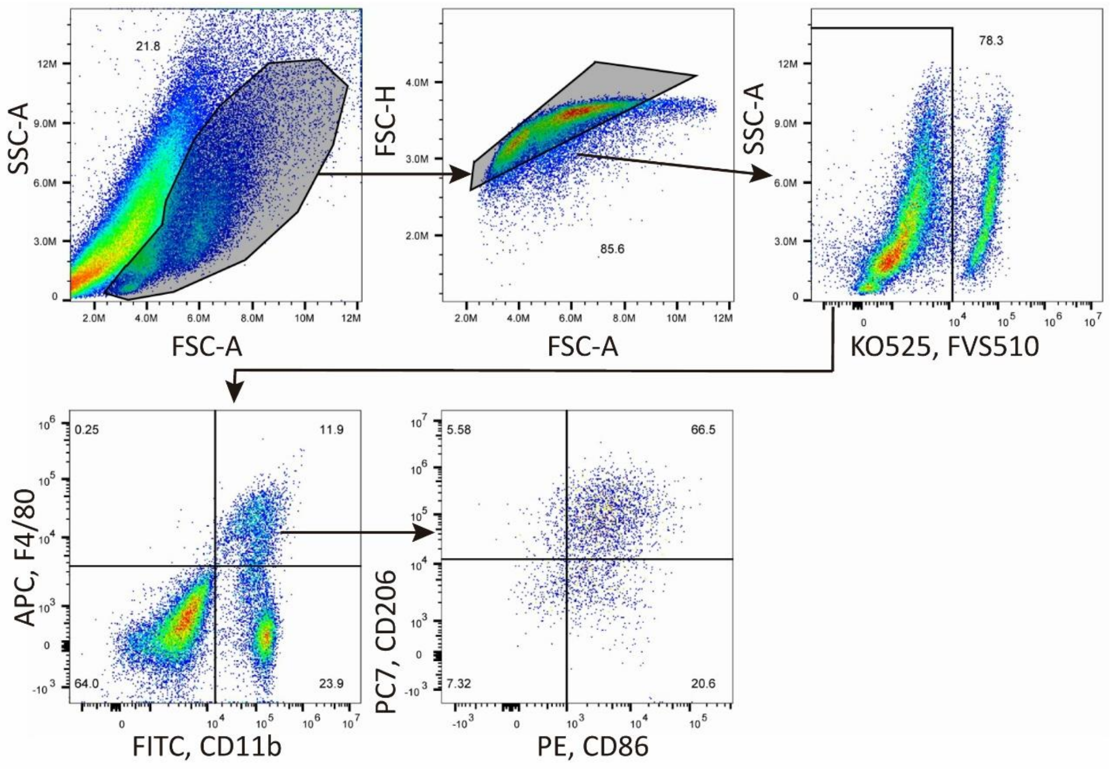

Flow cytometry gating strategy for macrophage populations (M1, M2, and total macrophages) in spleen, tumors, and tumor slices.

Supplementary Methods 3. Gating strategy for proliferation and activation of macrophages in macrophage alone or supernatant model in vitro.

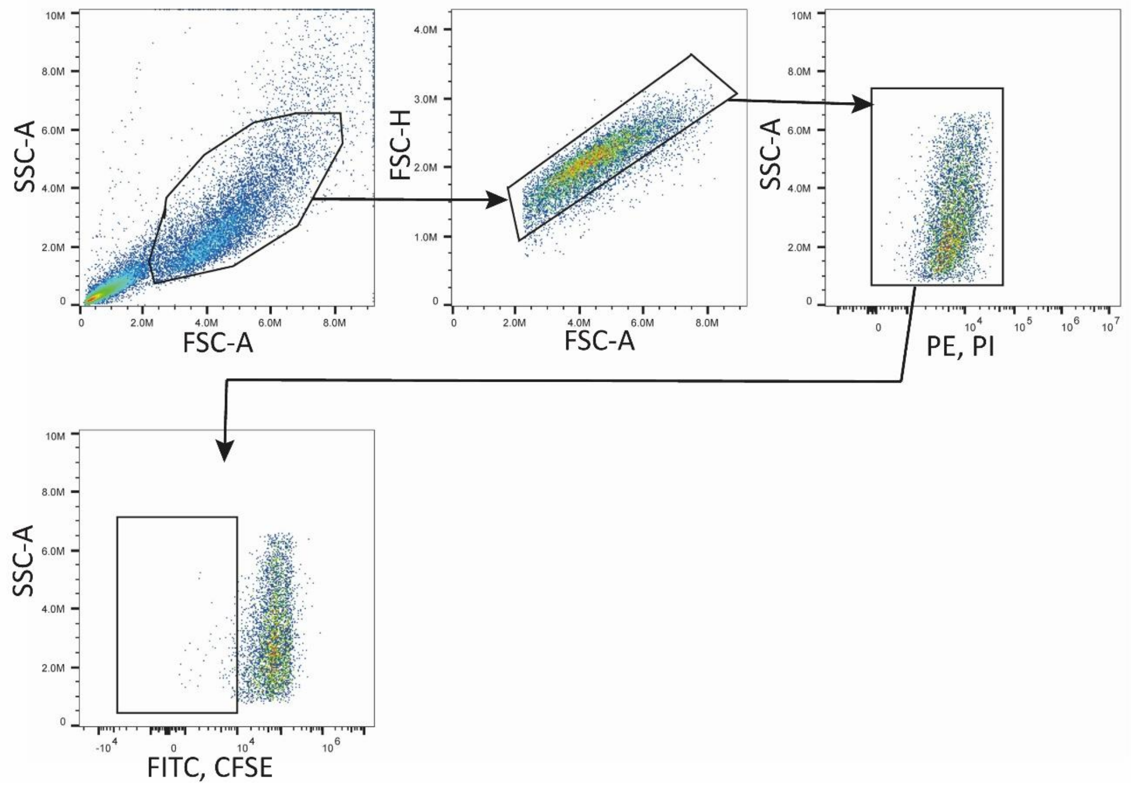

Supplementary Methods 4. Gating strategy for proliferation and activation of macrophages in the pretreatment or con-treatment model in vitro.

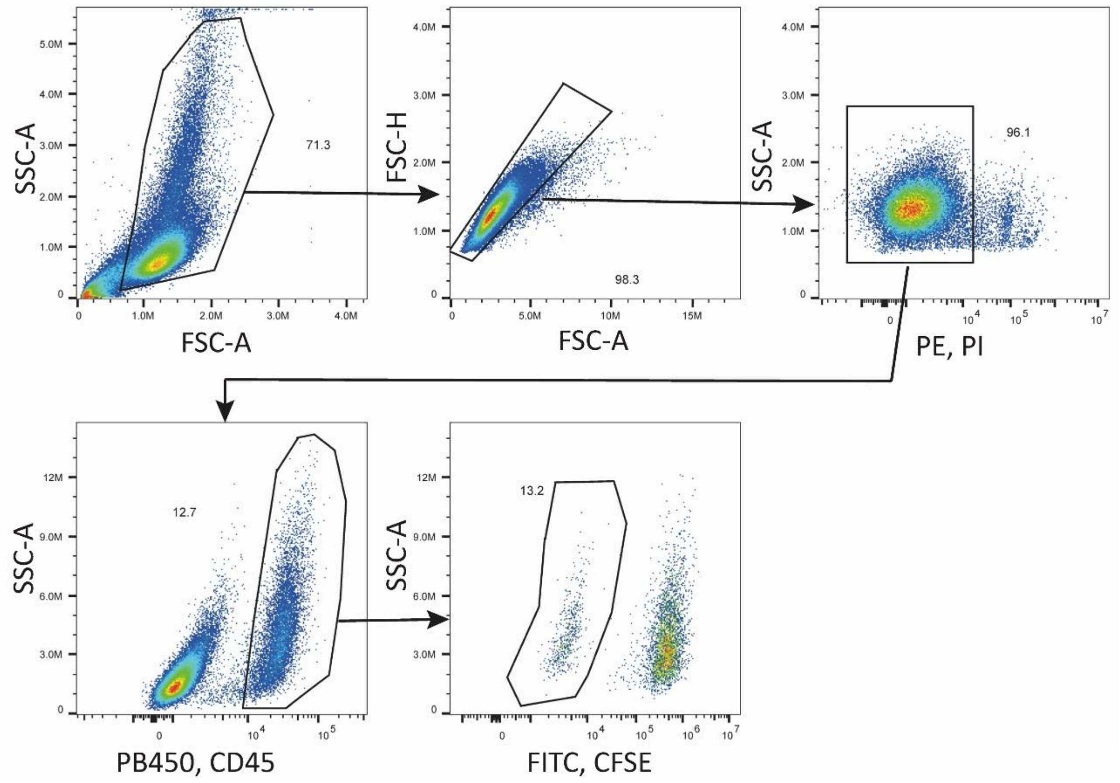

Supplementary Methods 5. Gating strategy for proliferation and activation of CD8+T cells in the coculture system in vitro.

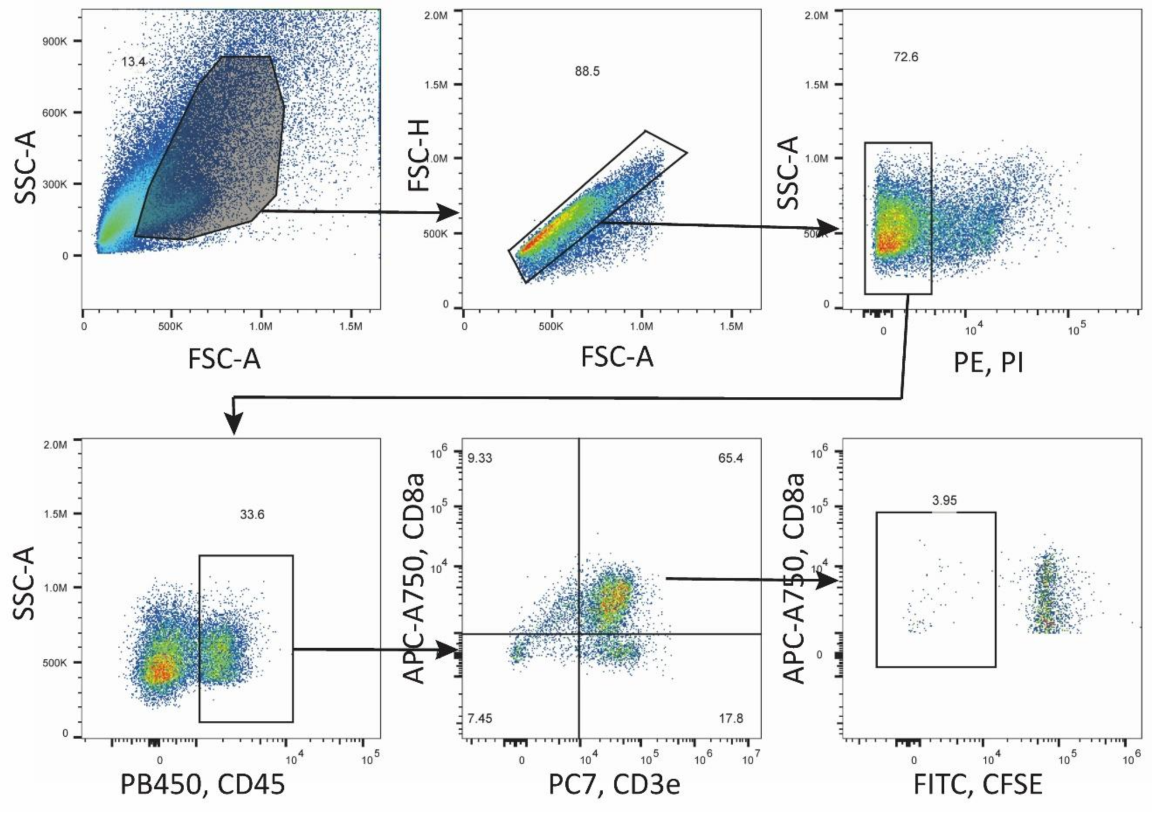

The following file contains uncropped images of scanned immunoblots shown in Fig. 5c, Fig. 5d, Fig. 5l, Fig. 5p, Fig. 6c, supplementary Fig. 9C, supplementary Fig. 10A, supplementary Fig. 10G, supplementary Fig. 11B, and supplementary Fig. 11C.

Fig. 5c

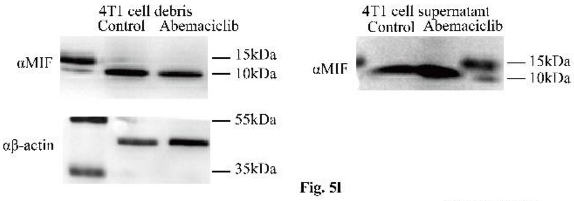

Fig. 5d

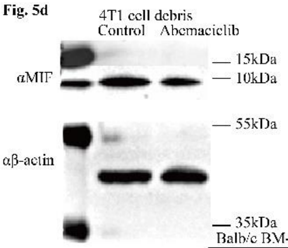

Fig. 5d

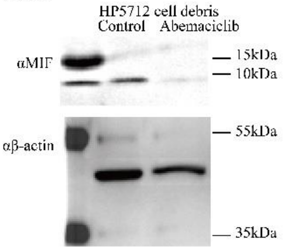

Fig. 5l

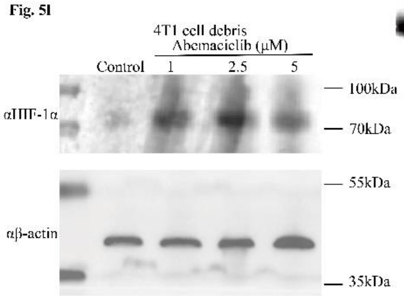

Fig. 5p

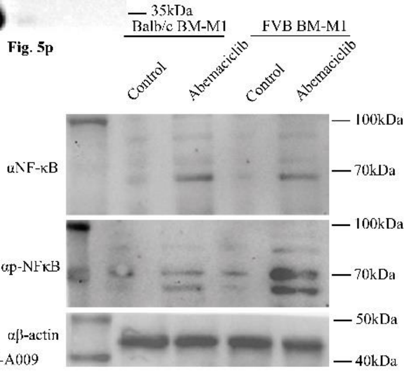

Fig. 6c

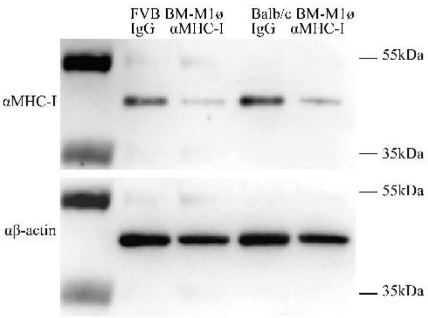

supplementary Fig. 9C

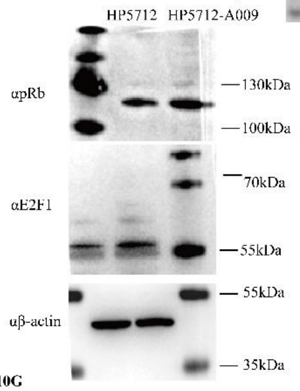

supplementary Fig. 10A

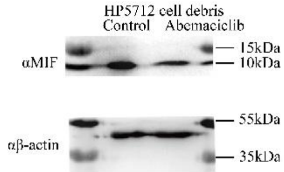

supplementary Fig. 10G

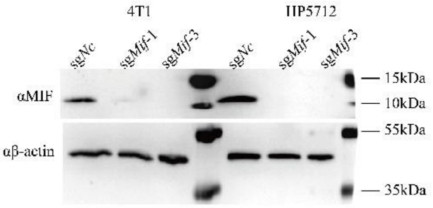

supplementary Fig. 11B

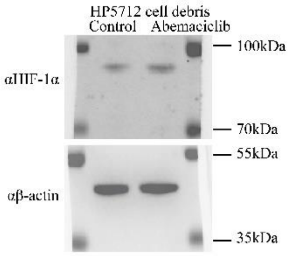

supplementary Fig. 11C

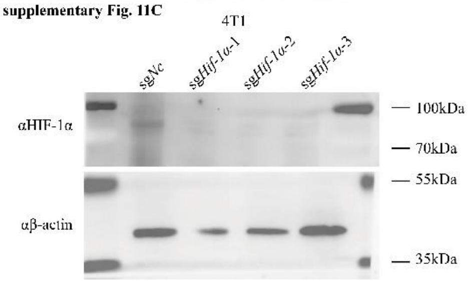

Supplement: Supplementary file 1 — Supporting Information [file ADVS-13-e11330-s001.pdf]
